# Supplementary material for: Exemestane plus everolimus and palbociclib in metastatic breast cancer: clinical response and genomic/transcriptomic determinants of resistance in a phase I/II trial
Source: Nat Commun. 2024 Mar 19;15:2446. doi: 10.1038/s41467-024-45835-6 (PMC10951222; doi:10.1038/s41467-024-45835-6)
Supplement: Supplementary file 2 — Description of Additional Supplementary Files [file 41467_2024_45835_MOESM2_ESM.pdf]

Description of Additional Supplementary Files

Supplementary Data 1. Original study protocol and pharmacokinetic profile of the phase Ib portion of the clinical trial.

Supplementary Data 2. Processed genomic and transcriptomic dataset.

Supplementary Data 3. Patient-level clinical characteristics of cohort. Excel file, 2 tabs.(Tab 1) Clinical trial patient- level metadata. (Tab 2) Additional patient-level metadata.

Supplementary Data 4. Sample-level clinical information of cohort. Excel file, 2 tabs. (Tab 1) Sample-level metadata for samples with exome or transcriptome sequencing data. (Tab 2) Sample-level metadata for samples with targeted panel sequencing data.

Supplementary Data 5. Genomic information (mutation and copy number calls) of cohort. Excel file, 4 tabs. (Tab 1) Single nucleotide variants (SNVs) and Indels from whole-exome sequencing (WES). (Tab 2) Copy number variants (CNVs) from WES. (Tab 3) Mutation calls for tumor samples using a targeted panel. (Tab 4) CNVs for tumor samples using a targeted panel. (Tab 5) Selected oncogenes and tumor suppressor genes for WES CNVs.

Supplementary Data 6. Clinical, genomic, and transcriptomic features associated with resistance to CDK4/6 inhibitors and anti-estrogen treatment used in Figure 4 and Supplementary Figure S3. Excel file, 2 tabs. (Tab 1) Features used in Figure 4. (Tab 2) Features used in Supplementary Figure S3.

Supplementary Data 7. Treatment timeline for the 6 patients included in the tumor evolutionary analysis of Figures 5 and 6.

Supplementary Data 8. Results of comparing the Hallmark signature activity in baseline tumors with or without clinical benefit used in Figure 7.
