# Supplementary material for: Exemestane plus everolimus and palbociclib in metastatic breast cancer: clinical response and genomic/transcriptomic determinants of resistance in a phase I/II trial
Source: Nat Commun. 2024 Mar 19;15:2446. doi: 10.1038/s41467-024-45835-6 (PMC10951222; doi:10.1038/s41467-024-45835-6)
Supplement: Supplementary file 3 — Supplementary Data 1 [file 41467_2024_45835_MOESM3_ESM.zip › Supplementary Data 1/Trial Protocol_Initial.pdf]

NCI Protocol #: N/A.

DF/HCC Protocol #: 16-177

TITLE: A Phase 1b/2a Study Of Palbociclib In Combination With Everolimus And Exemestane In Postmenopausal Women With Estrogen Receptor Positive and HER2 Negative Metastatic Breast Cancer.

Coordinating Center: Dana-Farber Cancer Institute

***Principal Investigator (PI):** Sara Tolaney, MD MPH
Dana-Farber Cancer Institute
450 Brookline Ave. (CLS 11059) Boston, MA 02215
Telephone – 617-632-5743
Fax – 617-632-1930
Sara_Tolaney@dfci.harvard.edu

Other Investigators: Arlindo R. Ferreira, MD MSc
Dana-Farber Cancer Institute
450 Brookline Ave. (CLS 11059) Boston, MA 02215
ArlindoR_Ferreira@dfci.harvard.edu

Nikhil Wagle, MD
Dana-Farber Cancer Institute
450 Brookline Ave. (CLS 11059) Boston, MA 02215
Nikhil_Wagle@dfci.harvard.edu

Statistician:

William Barry, PhD
Department of Biostatistics and Computational Biology
Dana-Farber Cancer Institute
450 Brookline Ave. (CLS 11007)
Boston, MA 02215
Ph: 617-632-2193
Fax: 617-632-5444

Hao Guo, MSc
Department of Biostatistics and Computational Biology
Dana-Farber Cancer Institute
450 Brookline Ave. (CLS 11059) Boston, MA 02215
Tel: 617-632-3626
Fax: 617-632-5444
Email: Hao_Guo@dfci.harvard.edu

Study Coordinator:

Kathryn Josephs
Dana-Farber Cancer Institute
450 Brookline Ave.
Boston, MA 02215
Ph: 617-632-3800
Fax: 617-632-3550
Email: kjosephs@partners.org

Responsible Research Nurse:

TBD

Responsible Data Manager:

N/A

NCI-Supplied Agent(s): N/A.

Other Agent(s):
Palbociclib, Pfizer
Everolimus, Commercial (Novartis)
Exemestane, Pfizer

IND #: TBD.
IND Sponsor: TBD.

Protocol Type / Version # / Version Date: Original / Version 1 / June 24, 2016

SCHEMA

Phase Ib Dose Escalation Trial Part

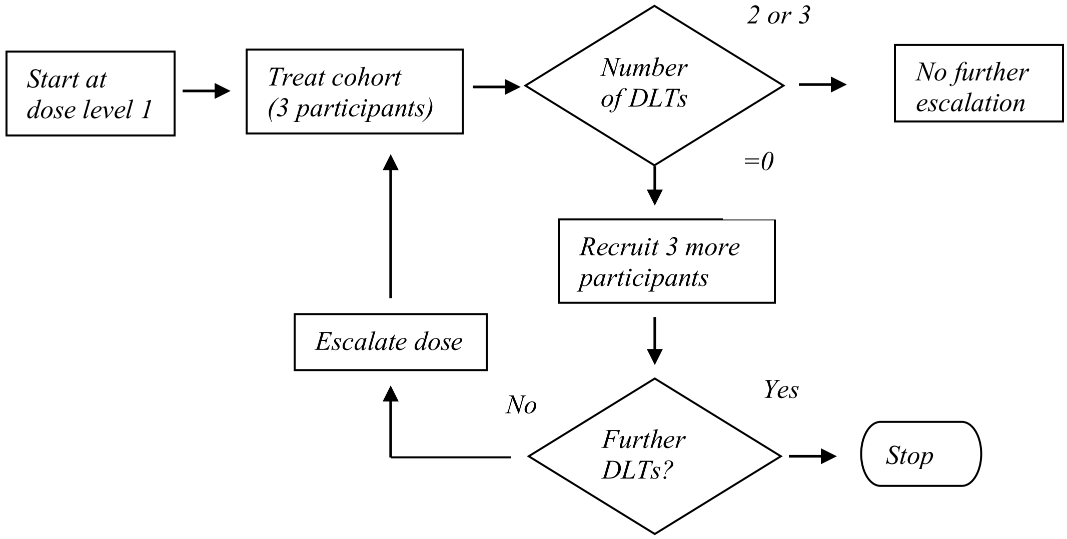

Cycle Length: 28 days

Non-randomized Phase IIa Single Arm Trial Part

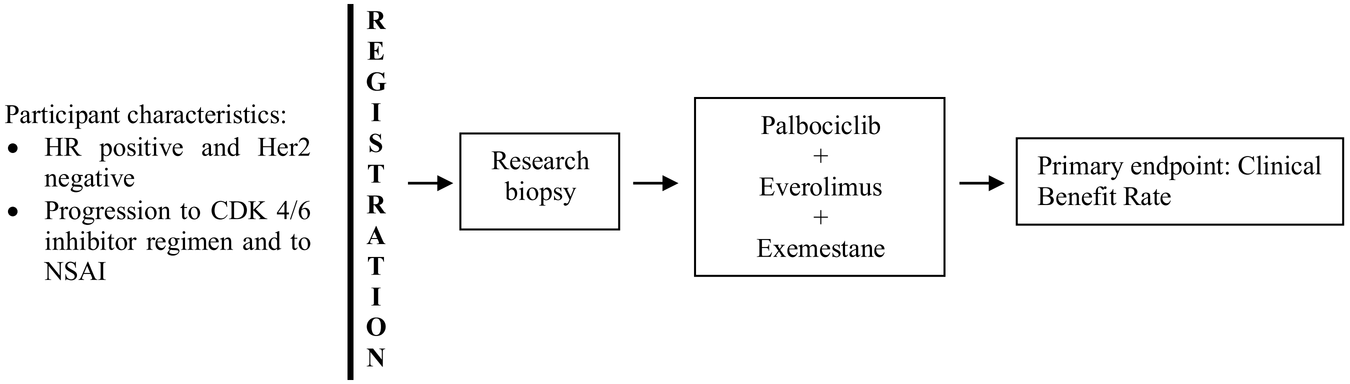

Cycle Length: 28 days

* Abbreviations: CDK 4/6 – cyclin dependent kinase 4/6; NSAI – non-steroidal aromatase inhibitor.

TABLE OF CONTENTS

SCHEMA 3

1. OBJECTIVES 6
    - 1.1 Study Design 6
    - 1.2 Primary Objectives 6
    - 1.3 Secondary Objectives 6

2. BACKGROUND 7
    - 2.1 Breast Cancer Epidemiology 7
    - 2.2 Treatment Of Postmenopausal Women With Hormone-Receptor Positive Her2 Negative Metastatic Breast Cancer 7
    - 2.3 Cyclin-Dependent Kinases In Breast Cancer 8
    - 2.4 IND Agent(s) 9
    - 2.5 Study rationale 17
    - 2.6 Correlative Studies Background 18

3. PARTICIPANT SELECTION 20
    - 3.1 Inclusion Criteria 20
    - 3.2 Exclusion Criteria 22
    - 3.3 Inclusion of Women and Minorities 24

4. REGISTRATION PROCEDURES 24
    - 4.1 General Guidelines for DF/HCC Institutions 24
    - 4.2 Registration Process for DF/HCC Institutions 24
    - 4.3 General Guidelines for Other Investigative Sites 24
    - 4.4 Registration Process for Other Investigative Sites 24

5. TREATMENT PLAN 24
    - 5.1 Treatment Regimen 24
    - 5.2 Definition of Dose-Limiting Toxicity (DLT) 26
    - 5.3 Definition of maximum tolerated dose (MTD) 28
    - 5.4 Intra-participant dose escalation 28
    - 5.5 Doses for Phase II 28
    - 5.6 Pre-Treatment Criteria 29
    - 5.7 Agent Administration 29
    - 5.8 General Concomitant Medication and Supportive Care Guidelines 30
    - 5.9 Criteria for Taking a Participant Off Protocol Therapy 32
    - 5.10 Duration of Follow Up 32
    - 5.11 Criteria for Taking a Participant Off Study 33

6. DOSING DELAYS/DOSE MODIFICATIONS 33

7. ADVERSE EVENTS: LIST AND REPORTING REQUIREMENTS 37
    - 7.1 Expected toxicities 37
    - 7.2 Adverse Event Characteristics 39

7.3 Expedited Adverse Event Reporting 40
7.4 Expedited Reporting to the Food and Drug Administration (FDA) 40
7.5 Expedited Reporting to Pfizer 41
7.6 Expedited Reporting to Hospital Risk Management 42
7.7 Routine Adverse Event Reporting 42

8. PHARMACEUTICAL INFORMATION 42
8.1 Palbociclib 42
8.2 Everolimus 45
8.3 Exemestane 47

9. BIOMARKER, CORRELATIVE, AND SPECIAL STUDIES 49
9.1 cfDNA Collection 49
9.2 Exome Analysis 50
9.3 Pharmacokinetic Studies 50
9.4 Tissue Collection: The molecular underpinnings of CDK 4/6 inhibitors response and resistance 51

10. STUDY CALENDAR 54

11. MEASUREMENT OF EFFECT 57
11.1 Antitumor Effect – Solid Tumors 57

12. DATA REPORTING / REGULATORY REQUIREMENTS 64
12.1 Data Reporting 64
12.2 Data Safety Monitoring 65
12.3 Multicenter Guidelines 65
12.4 Collaborative Agreements Language 65

13. STATISTICAL CONSIDERATIONS 65
13.1 Study Design/Endpoints 65
13.2 Sample Size, Accrual Rate and Study Duration 66
13.3 Stratification Factors 68
13.4 Interim Monitoring Plan 68
13.5 Analysis of Primary Endpoints 68
13.6 Analysis of Secondary Endpoints 68
13.7 Reporting and Exclusions 69

14. PUBLICATION PLAN 69

REFERENCES 70

APPENDIX A PERFORMANCE STATUS CRITERIA 79

APPENDIX B INFORMATION ON POSSIBLE DRUG INTERACTIONS 80

APPENDIX C DRUG DIARY 84

1. OBJECTIVES

We hypothesize that the treatment of participants with estrogen receptor (ER)-positive and Her2-negative metastatic breast cancer with palbociclib plus everolimus and exemestane after progression to a CDK4/6 inhibitor based regimen and to a non-steroidal aromatase inhibitor will be safe, tolerable and lead to improved disease outcomes.

1.1 Study Design

This is a single center, single arm, open-label, phase Ib/ IIa study. Both parts of the trial (Ib and IIa) will be conducted in participants with ER+/Her2− advanced breast cancer that progressed on a CDK 4/6 inhibitor regimen and a NSAI.

The phase Ib part of the study is designed to estimate the maximum tolerated dose (MTD) and/or the recommended phase II dose (RP2D) for the combination of palbociclib with everolimus and exemestane (P-E-E). The phase Ib part of the study will also assess the safety and tolerability of the P-E-E combination, including PK of everolimus and palbociclib in the triple combination. It will be followed by a phase IIa part to assess the clinical safety, tolerability and efficacy of the triple combination of P-E-E in participants with ER+, Her2− advanced breast cancer refractory to a prior CDK 4/6 inhibitor regimen and to a NSAI.

Each cycle will be 28 days with Palbociclib administered orally, once daily for 21 consecutive days followed by a 7-day break; everolimus and exemestane will be administered orally, once daily on a 28 day schedule. Participants will be re-staged every 8 weeks and are permitted to be treated until progression of disease, development of unacceptable toxicity, withdrawal of informed consent or death, whichever occurs first. If clinical benefit rate (CBR) of the combination is equal or superior to 65%, the regimen will be considered worthy of further study.

1.2 Primary Objectives

- Phase Ib: To describe the safety and tolerability and define MTD/RP2D of palbociclib in combination with everolimus and exemestane in participants with ER+ Her2− advanced breast cancer;
- Phase IIa: To estimate the activity of palbociclib in combination with everolimus and exemestane, as defined by clinical benefit rate (CBR), in participants with ER+ Her2− advanced breast cancer.

1.3 Secondary Objectives

- Phase Ib: To describe the PK profile of everolimus and palbociclib in the triple combination and evaluate the DDI potential (effect of palbociclib on the PK profile of everolimus);
- Phase Ib and IIa: To investigate biomarkers of sensitivity and resistance to CDK 4/6

inhibitors at the DNA, RNA and protein levels respectively via whole exome sequencing, RNA Seq and immunohistochemistry of paired tumor biopsies;

- Phase IIa: To estimate the activity of palbociclib in combination with everolimus and exemestane, as defined by overall response rate (ORR), disease control rate (DCR), duration of response (DOR) and progression free-survival (PFS) according to investigator assessment, in participants with ER+ Her2− advanced breast cancer.

2. BACKGROUND

2.1 Breast Cancer Epidemiology

Breast cancer (BC) is the most frequently diagnosed cancer and the leading cause of cancer related deaths in women worldwide, accounting for 23% (1.38 million) of all new cancer cases and 14% of cancer deaths (more than 400,000) each year.[1]

In the US, the estimated incidence of BC is 122.2 cases per 100 000 women per year leading to the diagnosis of approximately 233,000 new cases of invasive BC in 2014, of which about 40,000 women are expected to die from.[2] Furthermore, approximately 40% of those participants treated with curative intent will eventually develop metastatic BC.[3] For women with metastatic disease, treatment is palliative and overall median life expectancy, despite varying between subtypes of BC, ranges from 17 to 54 months.[4]

2.2 Treatment Of Postmenopausal Women With Hormone-Receptor Positive Her2 Negative Metastatic Breast Cancer

Expression of the estrogen receptor (ER) and/or progesterone receptor (PgR) is present in up to 70% of the cases of BC and is a strong prognostic marker.[5] Estrogen deprivation is the backbone of treatment in participants with hormone receptor positive (HR+) Her2 negative advanced BC and ER expression is the strongest predictor of response to such endocrine therapies.[6,7] Postmenopausal women account for more than 80% of the women with BC, and in such group endocrine therapy options for those ER+ advanced BC (locally advanced, recurrent, or metastatic BC) include selective estrogen receptor modulators (SERM; tamoxifen), estrogen receptor antagonists (fulvestrant), selective nonsteroidal aromatase inhibitors (NSAI; anastrozole and letrozole) and steroidal aromatase inhibitors (exemestane).[8] Blocking estrogen signaling with tamoxifen has been the main approach in treatment for ER positive BC for over 30 years. Tamoxifen is indicated for the treatment across the whole continuum of HR+ BC in premenopausal women, ranging from risk reduction in women at high risk of developing BC to treatment of metastatic disease. In postmenopausal women, aromatase inhibitors (AI) reduce peripheral estrogen synthesis by blocking the conversion of androgens to estrogens in non-ovarian tissues; synthesis in these tissues is the primary source of estrogens in postmenopausal women. Aromatase inhibitors are generally used as the first option of therapy for postmenopausal women with ER+ BC, both in the adjuvant and metastatic setting.[9,8,10,11] Despite the broad spectrum of available options for participants with ER+ advanced BC, most eventually develop resistance to hormonal deprivation. One mechanism of endocrine resistance is aberrant signaling via phosphatidylinositol 3-kinase (PI3K)/Akt/mammalian target of rapamycin (mTOR) pathway.[12,13] Hyperactivation of PI3K/mTOR is observed in endocrine-resistant BC cells, and

treatment with mTOR inhibitors, including rapamycin analogs, reverses this resistance.[14] Growing evidence also supports a close interaction of the mTOR pathway with ER signaling. S6 kinase-1 (S6K1), a substrate of mTOR Complex 1 (mTORC1), phosphorylates the activation domain AF-1 of the ER, and stimulates ligand-independent activation of the receptor.15,16 Everolimus is a rapamycin derivative that inhibits mTORC1 through allosteric binding; it does not inhibit mTORC2.[17] Everolimus combined with AIs in preclinical models of ER-positive (ER+), hormone-sensitive and hormone-resistant BC results in G1 arrest and enhanced apoptosis.[18] In the clinical arena, everolimus combined with exemestane is active in participants with advanced ER+ BC resistant to previous hormonal deprivation therapy with nonsteroidal aromatase inhibitor, either in the adjuvant setting or to treat advanced disease (the BOLERO-2 study[19]). Although treatment options with targeted agents has expanded, cytotoxic agents, typically anthracyclines and taxanes and more recently capecitabine, are also used to treat participants with a significant tumor burden and symptomatic visceral disease. Capecitabine monotherapy in participants without previous exposure to taxanes and anthracyclines showed PFS between 4.1 and 7.9 months, and OS between 18.6 and 29.4 months 20–24, and is included in treatment guidelines as an effective option for first line treatment in participants with advanced BC. More recently, CDK 4/6 inhibitors joined the therapeutics portfolio for this subset of participants, as discussed ahead.

2.3 Cyclin-Dependent Kinases In Breast Cancer

The cell cycle includes a set of consecutive phases that ultimately lead to mitosis; in this setting, a group of kinases, referred as cyclin-dependent kinases, and their regulatory counterpart, referred as cyclins, have a central governing role.[25] Cyclin D, in association with CDK4/6, controls the early transition of the cell cycle between G1 and S phases, thus setting an early checkpoint in cell cycle. In specific, the interaction of cyclin D with CDK4/6 leads to the activation of the kinase activity of the latter. A widely recognized target of this complex is the tumor suppressor Rb (and other Rb 'pocket' protein family members, such as p130 and p107) that is inactivated after CDK4/6 mediated phosphorylation (revised in refs[26,27]). As a consequence, downstream E2F family transcription factors are released, and E2F-responsive genes activated. This activation leads to the expression of a plethora of genes essential for DNA synthesis and continuing cell cycle progression. Strong preclinical[28,29] and clinical evidence[30–32] support the relevance of this target for cancer therapy, in specific for ER-positive BC. Data from The Cancer Genome Atlas highlight the importance of the Cyclin/CDK/Rb pathway in luminal BC.[33] Abnormalities that result in CDK activation are highly enriched in the luminal A and B molecularly defined subgroups, ~85% of which were ER+/Her2−. Cyclin D1 amplifications were observed in 29% and 58% of the luminal A and B subtypes, respectively, and CDK4 amplifications were observed in 14% and 25% of luminal A and B subtypes, respectively. Luminal A subtype tumors also have loss of CDKN2. The luminal subtypes maintain expression of Rb[33], which would be essential for clinical benefit from treatment targeting this pathway, as a CDK4/6 inhibitor. A recent phase II trial led to the conditional approval by the US Food and Drug Administration of palbociclib, an oral, potent, CDK4/6 inhibitor, in association with letrozole (a NSAI) as first-line treatment for metastatic ER-positive, HER2-negative BC.[31] Unfortunately, despite the initial benefit derived from the addition of palbociclib for the overall sample of luminal BC participants enrolled in this trial (median progression free-survival of approximately 20 months compared to 10 months in the letrozole monotherapy arm), after 3

years of study enrollment more than 70% of the participants had a disease progression event (secondary resistance). Achieving improved disease control with endocrine therapy-based regimens is still an unmet medical need for this participant population. The co-administration of agents targeting the PI3K/mTOR pathway and agents targeting the CDK4/6 pathway is still a relatively unexplored concept in the context of progression to first line ET in combination with palbociclib (study rationale detailed in Section 2.5).

2.4 IND Agent(s)

2.4.1 *Palbociclib*

Palbociclib (PD-0332991), an orally active pyridopyrimidine, is a potent first-in-class, highly selective reversible inhibitor of CDK4 and CDK6 (IC50 = 11 nM; Ki = 2 nM) with a molecular weight of 447.53. Data from nonclinical studies indicate that palbociclib may have cytoreductive as well as cytostatic effects on tumor cells. The compound prevents cellular DNA synthesis by prohibiting progression of the cell cycle from G1 into the S phase, as demonstrated both in laboratory models and in early clinical trials. CDK4 and CDK6 control G1 to S phase transit by binding to D-type cyclins.[34–36] The CDK4/6/Cyclin D1 complex phosphorylates the retinoblastoma susceptibility (RB1) gene product (Rb), releasing the E2F and DP transcription factors that drive expression of genes required for S-phase entry. CDK activity and G1 progression is negatively regulated by Cip-Kip (P27, P21) and INK4 family, typified by p16.[37–41] Overexpression of p16 in cells with normal Rb inhibits both CDK4 and CDK6-associated kinase activity and Rb phosphorylation, with subsequent cell cycle arrest.[42,43] There is a strong link between the actions of estradiol and the G1-S phase transition, where the estradiol effector is the cyclin D1-CDK4/6-Rb complex.[44] Cyclin D1 is a direct transcriptional target of ER[45,46] and microinjection of antibodies to cyclin D1 inhibits estrogen-induced S-phase entry. In addition, anti-estrogen induced growth arrest of ER+ breast cancer cells is accompanied by decreased cyclin D1 expression[47], while endocrine resistance is associated with persistent cyclin D1 expression and Rb phosphorylation.[48] Consistent with the notion that the main function of cyclin D1 is to activate CDK4/6, its oncogenic activity is dependent on CDK4/6-associated kinase activity[49] and CDK4/6 inhibitors are most effective in tumors with gene amplification and overexpression of cyclin D1[28,50,51], which is common in ER+ breast cancer. Genetic aberrations leading to hyperactivation of cyclin D1-CDK4/6 is particularly common in ER+ breast cancer[29], consistent with its critical role in the tumorigenesis of this cancer subtype, making CDK4/6 inhibitors particularly attractive agents for ER+ breast cancer.

2.4.1.1 Palbociclib Preclinical Data

Palbociclib preclinical data indicate that it may be expected to have direct effect on growth arrest as well as potential secondary cytoreductive activity. Single agent palbociclib has shown antiproliferative effects (selective G1 arrest) on Rb-positive cancer cells in vitro and in vivo[28] where palbociclib activity was associated with reduced Rb-phosphorylation and decreased expression of the cell proliferation marker Ki67. Palbociclib showed no activity in Rb-negative tumor cell xenografts, consistent with CDK4/6 inhibition as the sole mode of action.[28] Treatment of cultured tumor cells with palbociclib causes growth arrest that is accompanied by the inhibition of specific Rb phosphorylation by CDK4 or CDK6 on residues serine -780 and -795 of Rb. The IC50 values for reduction of Rb phosphorylation at serine -780 and -795 in MDA-MB-

435 breast carcinoma cells were 0.066 and 0.063 μM, respectively. The IC50 values for reduction of Rb phosphorylation are similar to the IC50 values of inhibition of thymidine incorporation across a range of cultured tumor and normal cells. Palbociclib was tested in vitro on molecularly characterized human breast cancer cell lines. Results from these experiments indicate that those cell lines that are more sensitive to palbociclib (IC50 < 150 nM) have low levels of CDKN2A (p16) and high levels of Rb1, while resistant cell lines show the opposite characteristics. In this study, ER+ breast cancer seems to be particularly appropriate for treatment with palbociclib; sensitive cell lines in this panel represent mostly the luminal ER+ subtype. The combination of palbociclib with tamoxifen has been tested in vitro in ER+ breast cancer cell lines indicating a synergistic interaction and provided a biologic rationale for evaluating the combination of palbociclib with anti-hormonal therapy in the clinic. In nonclinical studies, palbociclib and its active lactam metabolite, PF-05089326, demonstrated little or no inhibition of cytochrome P450 (CYP) 1A2, CYP2B6, CYP2C8, CYP2C9, CYP2C19, or CYP2D6 enzyme activities and thus, showed low potential for PK DDI with drugs that are substrates for these CYPs. However, palbociclib and its metabolite, PF-05089326, caused time-dependent inhibition of CYP3A-mediated midazolam 1'-hydroxylase and testosterone 6β-hydroxylase activities and, therefore, may have the potential for PK DDI with drugs for which CYP3A-mediated metabolism constitutes the primary mechanism of clearance. Palbociclib did not cause induction of CYP1A2, CYP2B6, CYP2C8, or CYP3A4 messenger ribonucleic acid expression and/or enzyme activity in vitro in human hepatocytes at concentrations exceeding 50-fold of the palbociclib unbound steady-state plasma Cmax determined at therapeutic doses in humans; thus, the potential for palbociclib to induce these enzymes is considered to be low at clinically relevant concentrations. The potential for palbociclib to inhibit the activities of selected uridine diphosphate glucuronosyltransferase (UGT) enzymes (UGT1A1, UGT1A4, UGT1A6, UGT1A9, and UGT2B7) was also assessed, and the likelihood of DDI associated with inhibition of these Phase 2 metabolizing enzymes at clinically relevant concentrations is considered low. Inhibition of efflux transporters (p-glycoprotein [P-gp] and breast cancer resistant protein [BCRP]), hepatic uptake transporters (organic anion-transporting polypeptide (OATP) 1B1 and OATP1B3), hepatic efflux transporter (bile salt export pump [BSEP]), and renal transporters (organic anion transporter [OAT] 1, OAT3, and organic cation transporter [OCT] 2) by palbociclib was assessed in vitro and was considered to be unlikely at clinically relevant palbociclib concentrations. In vitro, palbociclib is metabolized mainly by CYP3A and sulfotransferase 2A1 (SULT2A1) enzymes. Drugs that are known to induce or inhibit the activities of these enzymes may alter the clearance and systemic exposure of palbociclib.

2.4.1.2 Palbociclib Pharmacokinetic (PK) Data

Pharmacokinetic data have been collected in at least 8 clinical studies for a total of over 250 advanced cancer participants and 30 healthy volunteers (A5481001, A5481002, A5481003, A5481004, A5481008, A5481009, A5481010, and A5481011). In the FIP trial (A5481001) the exposure increased in a dose proportional manner over the dose range of 25 to 225 mg QD following palbociclib administration on Days 1 and 8 of Cycle 1, although some variability (low to moderate) around these doses was observed particularly at the 150 mg QD dose level. Following repeated daily dosing to steady state, palbociclib was absorbed with a median Tmax of ~4 hours. The mean palbociclib Vz/F was 2583 L, which is significantly greater than total body water (42 L), indicating that palbociclib extensively penetrates into peripheral tissues.

Palbociclib was eliminated slowly; the mean elimination half-life (t1⁄2) was 28.8 hours and the mean CL/F was 63.1 L/hour. Palbociclib accumulated following repeated dosing with a median Rac of 2.4, which is consistent with the terminal half-life. The effect of food on the bioavailability of palbociclib, when administered as the commercial free base capsule, was investigated in Study A5481021. The administration of the free base formulation of palbociclib with food (including a high fat or a low fat meal given together with palbociclib, or moderate fat meals given 1 hour before and 2 hours after palbociclib) resulted in more uniform drug absorption and significantly reduced the intersubject variability in drug exposure when compared to the administration of free base formulation of palbociclib in a fasted state. The relative bioavailability of the commercial free base capsule administered with food and the isethionate capsule administered under overnight and minimal fasting conditions was investigated in Study A5481036. The two fasting conditions for administration of isethionate capsules represent the 2 extreme scenarios for compliant palbociclib dosing with regard to food intake in the pivotal Phase 1/2 efficacy trial, Study A5481003, in which participants were instructed to fast from 1 hour before until 2 hours after palbociclib dosing. The administration of palbociclib free base capsule formulation with food was found to be bioequivalent to palbociclib isethionate capsule formulation given under both the overnight and minimal fasting conditions. As a result of these findings, it is recommended that free base formulations of palbociclib be administered with food. Pharmacokinetic data are available from an itraconazole DDI study where the effect of multiple dosing of a potent CYP3A4 inhibitor, itraconazole (200 mg QD), on the single-dose PK of palbociclib (125 mg) was evaluated in 12 healthy fasted subjects (Study A5481016). Median palbociclib plasma concentrations were higher in the presence of itraconazole than those in the absence of itraconazole. Palbociclib mean plasma AUC from time 0 to infinity (AUC_{inf}) and C_{max} values increased approximately 87% and 34%, respectively, when administered in combination with itraconazole compared to when administered alone. Therefore, concomitant administration of agents known to be strong inhibitors of CYP3A isoenzymes (such as ketoconazole, miconazole, itraconazole, posaconazole, clarithromycin, erythromycin, telithromycin, nefazodone, diltiazem, verapamil, indinavir, saquinavir, ritonavir, nelfinavir, lopinavir, atazanavir, amprenavir, fosamprenavir, and grapefruit juice) should be avoided. Pharmacokinetic data are available from a rifampin DDI study where the effect of multiple dosing of a potent CYP3A4 inducer, rifampin (600 mg QD), on the single-dose PK of palbociclib (125 mg) was evaluated in 15 healthy fasted subjects (Study A5481017). Median palbociclib plasma concentrations were substantially lower in the presence of rifampin than those in the absence of rifampin. Palbociclib mean plasma AUC from time 0 to infinity (AUC_{inf}) and C_{max} values decreased approximately 85% and 70%, respectively, when administered in combination with rifampin compared to when administered alone. Therefore, co-administration of palbociclib with strong CYP3A inducers (such as phenobarbital, rifampin, phenytoin, carbamazepine, rifabutin, rifapentin, clevidipine, and St. John's Wort) should be avoided. Palbociclib and PF-05089326 caused time-dependent inhibition of CYP3A in vitro assays. Pharmacokinetic data are available from a midazolam DDI study where the effect of multiple dosing of palbociclib (125 mg QD) on the single-dose PK of a sensitive CYP3A4/5 probe substrate, oral midazolam (2 mg), was evaluated in 26 healthy women of non-childbearing potential (Study A5481012). When midazolam was administered with palbociclib 125 mg QD at steady-state, the mean midazolam plasma C_{max} and AUC_{inf} values increased approximately 37% and 61%, respectively, as compared with those determined after administration of midazolam alone. These results indicate that palbociclib is a weak time-dependent inhibitor of CYP3A.

2.4.1.3 Palbociclib Dose Rationale

Palbociclib has been used in a Phase 1 dose escalation Study (A5481001) in 74 participants with advanced cancer. Two dosing schedules were evaluated: a 3/1 (3 weeks on treatment/1 week off treatment) schedule and a 2/1 (2 weeks on treatment/1 week off treatment) schedule. All dose limiting toxicities (DLTs) observed in this study were related to myelosuppression and mainly consisted of Grade 3 neutropenia lasting more than 7 days after the end of the treatment cycle. However, the observed neutropenia was reversible and non-cumulative. The most common nonhematological adverse events included fatigue, anemia, diarrhea, constipation, vomiting and dyspnea, all with mild to moderate severity. A greater proportion of participants on the 2/1 schedule had treatment emergent adverse events (TEAEs) than participants on the 3/1 schedule although the proportion of participants with treatment related neutropenia was similar with respect to the 2 dosing schedules both. On this trial (A5481001) a total of 13/37 participants treated with Schedule 3/1 evaluable for efficacy experienced stable disease (SD), including 6 participants with SD lasting 40 weeks or longer. One of these participants was a woman with ER+ breast cancer who had previously received 7 lines of treatment for her disease. This participant remained on treatment for 80 weeks (7 cycles at 50 mg/d and 13 cycles at 75 mg/d) and eventually discontinued treatment due to disease progression. Additionally, one partial response (PR) was reported in a participant with testicular cancer. Based on the relatively improved safety profile of the 3/1 schedule, and the efficacy results from this study, the 3/1 schedule has been selected for further clinical development. The RP2D for this schedule was determined to be 125 mg/day and was further explored in combination with letrozole in the Phase I/II study in participants with advanced breast cancer described below.

2.4.1.4 Palbociclib Clinical Data

A phase II study with single agent palbociclib in 36 women with advanced breast cancer was reported at the American Society of Clinical Oncology (ASCO) 2013 Annual Meeting from 28 women who have completed cycle 1.[52] Palbociclib is given at 125 mg orally, days 1- 21 of a 28-day cycle. Of the 28 women, 18 (64%) women are HR+/HER2-, 2 (7%) are HR+/HER2+ and 8 (29%) HR-/HER2-negative. 90% had prior chemotherapy for metastatic disease (median 3 lines); 78% had prior hormonal therapy (median 2 lines). Grade 3/4 toxicities were limited to transient neutropenia (n=14; 50%) and thrombocytopenia (n=6; 21%). One episode of febrile neutropenia occurred in a participant with six previous chemotherapy regimens. All other toxicities were grade 1/2. Treatment was interrupted in 7 (25%) and dose reduced in 13 (46%) participants for cytopenias; 27/28 participants discontinued study for disease progression. The (PR + SD) >6 months was as follows: 4 participants (23%) in HR+/HER2-negative (n=18), 1 (50%) in HR+/HER2+ (n=2), 1 (13%) in HR-negative/HER2-negative (n=8). In conclusion, therapy with palbociclib alone is well-tolerated, and demonstrates clinical benefit in participants with all subtypes of breast cancer and despite progression on prior hormonal- and chemotherapy. Translational studies examining molecular predictors of response are in progress. A randomized, multicenter active-controlled Phase I/II Study (A5481003) was designed to assess the efficacy, safety and PK of letrozole 2.5 mg QD in combination with palbociclib 125 mg QD (schedule 3/1) versus single agent letrozole 2.5 mg QD for the first-line treatment of ER+/HER2-negative advanced breast cancer in postmenopausal women. Letrozole was selected as the active control based on its worldwide approval and use as standard of care for the first-line hormonal treatment

of postmenopausal women with ER+ advanced breast cancer. Study A5481003 included a limited Phase I portion, aimed at confirming the safety and tolerability of the combination and excluding a PK interaction with the combination, and a randomized Phase II portion aimed at evaluating the efficacy and safety of letrozole in combination with palbociclib when compared to letrozole alone in the first-line treatment of postmenopausal participants with ER+/HER2-negative advanced breast cancer. The Phase II portion, also called PALOMA-1, consisted of 2 parts. In Part 1, participant selection was based only on ER/HER2 status. In Part 2, participants were prospectively selected also taking into account tumor CCND1 amplification and/or p16 loss. A total of 177 participants were enrolled in the study. Twelve (12) were enrolled in the Phase 1 portion and 165 (66 and 99 in Part 1 and 2, respectively) were enrolled in the Phase 2 portion. Results from the Phase 1 portion indicated no PK interaction between palbociclib and letrozole. The RP2D was determined to be 125 mg QD on Schedule 3/1 (3 weeks continuous treatment followed by 1week off treatment) in combination with letrozole 2.5 mg QD continuously. PRs were reported for 3 (33%) out of 9 participants with measurable disease (3 had bone-only disease). Another 5 participants (42%) had stable disease for ≥ 6 months and the clinical benefit rate (PR + SD ≥ 6 months) was 67%. Eight (8) participants discontinued from the study due to disease progression, including 2 participants with clinical progression, 1 participant withdrew consent, and 3 participants were still being followed. Two interim analyses for the Phase 2 portion of the study have been conducted. The results of the interim analyses showed consistent trend of clinically meaningful improvements in PFS (primary endpoint). In the first interim analysis (Part 1; N=66), the median PFS for the palbociclib plus letrozole arm was 18.2 months versus 5.7 months for the letrozole alone arm (HR=0.35; 95% CI: 0.17, 0.72; p=0.006). The second interim analysis (N=165) continued to demonstrate a statistically significant improvement in PFS (26.1 vs. 7.5 months, respectively; HR=0.37; 95% CI: 0.21, 0.63; p <0.001).[53] More recently, the final analysis demonstrated a statistically significant improvement in PFS for the palbociclib plus letrozole arm (20.2 months) versus the letrozole arm (10.2 months) with hazard ratio (HR=0.488; 95% CI: 0.319, 0.748, p=0.0004).[31] These results indicate that the combination of palbociclib with letrozole is well tolerated with a safety profile similar to that seen with either palbociclib or letrozole when administered alone. The most frequently reported treatment related adverse events included neutropenia, leukopenia, anemia, and fatigue. There were no cases of febrile neutropenia reported to date in this study. Overall, 8 participants in the combination arm were discontinued from the study treatment due to an adverse event, of which 5 were considered treatment-related (grade 3 neutropenia [n=4] and ischemic colitis) and 1 participant from the letrozole alone arm. Additionally, the combination demonstrated antitumor activity, which was consistent with the sensitivity of ER+ breast cancer observed in the preclinical models.

Please refer to the Full Prescribing Information for palbociclib for complete safety information: http://www.accessdata.fda.gov/scripts/cder/drugsatfda/.

2.4.2 *Everolimus*

Everolimus (RAD001) has been in clinical development since 1996 as an immunosuppressant in solid organ transplantation. Everolimus is approved in the United States for the prevention of organ rejection of kidney transplantation. In 2012 everolimus in combination with exemestane

was approved for advanced breast cancer in the US, EU and other countries. Everolimus is a derivative of rapamycin. It is a selective mTOR inhibitor, specifically targeting the mTOR-raptor (regulatory-associated protein of mTOR, Raptor) signal transduction complex 1 (mTORC1). Everolimus potently inhibits proliferation of endothelial cells[54,55] and has antiangiogenic activity in vivo.[55–58]

2.4.2.1 Everolimus Preclinical Data

Everolimus inhibits the proliferation of a wide range of human tumor cell lines *in vitro* at IC50s ranging from sub/low nM to µM. The antitumor efficacy of everolimus was compared to other compounds in a panel of six breast cancer xenograft models established after direct transplantation of participants' tumors onto nude mice [Report RD-2011-50492]. This panel included an ER+ model, HBCx-3 (XTS-181).[59] Everolimus given daily by oral gavage for 21 to 35 days at 20 mg/kg was well tolerated with no significant mean body weight loss. In all breast cancer models tested, tumor growth was significantly inhibited, and was particularly evident in the HBCx-3 (XTS-181) model with nine partial regressions in ten mice tested (-13.5% mean tumor volume regression, $p<0.001$). Based on data generated using human liver microsomes and microsomes from cells expressing single human cytochrome P450s enzymes, CYP3A4 was identified as the major enzyme involved in the microsomal biotransformation of everolimus. Everolimus inhibited competitively the metabolism of the CYP3A4 substrate cyclosporine with a Ki value of 2.3µmol/L (2204 ng/mL) under *in vitro* conditions.

2.4.2.2 Everolimus Pharmacokinetic (PK) Data

Everolimus is rapidly absorbed with a median t_{max} of one to two hours. The steady-state $AUC_{0\text{-}\tau}$ is dose-proportional over the dose ranges between 5 to 70 mg when given weekly and 5 and 10 mg when given daily. Steady-state was achieved within two weeks with the daily dosing regimen. C_{max} is dose-proportional between 5 and 10 mg for both the weekly and daily regimens. In healthy subjects, high fat meals reduced systemic exposure to a 10 mg dose of everolimus (as measured by AUC) by 22% and the peak plasma concentration C_{max} by 54%. Light fat meals reduced AUC by 32% and C_{max} by 42%. Food had no apparent effect on the post absorption phase concentration-time profile (Study RAD001C2120). The blood-to-plasma ratio of everolimus, which is concentration-dependent over the range of 5 to 5000 ng/mL, is 17% to 73%. The amount of everolimus confined to the plasma is approximately 20% at blood concentrations observed in cancer participants given everolimus 10 mg/day (DMPK R303044). Plasma protein binding is similar in healthy participants and in subjects with moderate hepatic impairment (approximately 74%, [Study RAD001A2303]). The major and nearly exclusive enzyme responsible for the metabolism of everolimus in man was CYP3A4 (DMPK (US) 1998/005; DMPK (CH) R99-2448).[60] Other CYP isoenzymes either do not metabolize everolimus or do so at very low rates. Everolimus is a moderate inhibitor of P-glycoprotein-like mediated efflux systems, although the compound has a high intrinsic permeability when P-glycoprotein is inhibited (DMPK (CH) 1997/417).[61,62] Following oral administration, everolimus is the main circulating component in human blood and contributes the majority of the overall pharmacologic activity (Study W107). Everolimus was also shown to increase exposure of exemestane: In [Study RAD001Y2301], average exemestane C_{min} and C_{2h} were 45% and 71% higher, respectively, when co-administered with everolimus. No specific excretion studies have been undertaken in cancer participants; however, data available from the transplantation setting

found the drug to be mainly eliminated through the feces.

2.4.2.3 Everolimus Clinical Data

Everolimus as single agent

In a multicenter, randomized phase II study, a daily dose of everolimus (10 mg) was evaluated in participants with advanced breast cancer, most commonly with ER+ disease previously treated with hormonal deprivation therapy. In the subgroup of 19 participants with ER-positive/human epidermal growth factor receptor 2 (Her2)-negative in this trial, one complete response, 2 partial responses, 3 stable disease for longer than 6 months, and 6 stable disease for less than 6 months were observed. Median progression-free survival (PFS) in this subset of 19 participants was 3.5 months (95% C.I.: 1.9 – 5.5 months, data source: NCI-Canada). An additional partial response was reported in a participant with ER-positive Her2−unknown disease.[63]

Everolimus in combination with endocrine therapy in ER+ breast cancer

The combination of everolimus with hormonal therapy has been assessed in different disease settings. In newly diagnosed participants with ER+ breast cancer, a neoadjuvant randomized 270-participant phase II study compared the combination of everolimus 10 mg QD and letrozole 2.5 mg QD to letrozole alone for 16 weeks of therapy prior to surgery. The overall response rate in the investigational everolimus + letrozole arm was higher than that with letrozole alone: 68% vs. 59% based on palpation (p = 0.062) and 58% vs. 47% based on ultrasound (p= 0.021) respectively, meeting the predetermined endpoint for efficacy. Additionally, there was a greater antiproliferative response in the investigational arm, with a decrease of the Ki67 proliferation index to <1 in 57% of participants in the everolimus arm compared to 30% of participants in the placebo arm (p<0.01).[64] A randomized phase III, double-blind, placebo-controlled study (the BOLERO-2 Study) demonstrated very significant improvements in treatment of HR+ breast cancer that had recurred or progressed on letrozole or anastrozole. Response rate, PFS, and clinical benefit rate were all significantly improved relative to exemestane monotherapy. The median progression-free survival (PFS) by local assessment was 7.8 months for everolimus + exemestane versus 3.2 months for exemestane (HR = 0.45; 95% CI: 0.38-0.54; P<.0001). Overall response rate (12.6 % vs 1.7%; P<.0001) and clinical benefit rate (51.3% vs 26.4%; P<.0001) were superior in the everolimus + exemestane arm versus exemestane + placebo. Analyses by central assessment showed a median progression-free survival of 11 months with everolimus versus 4.1 months with placebo (HR = 0.38; 95% CI: 0.31 – 0.48; P <.0001) confirming the results of the primary PFS analysis.[19]

Safety profile

The following adverse events are considered to be class-effects of mTOR inhibitors: stomatitis/oral mucositis/ulcers, infections and infestations, rash and similar events, cytopenia, hemorrhages, non-infectious pneumonitis, hyperglycemia/new-onset diabetes mellitus, renal events, and thromboembolism. The more common metabolic side effects reported with mTOR inhibitors result from inhibitory effects on mTOR-regulated lipid and glucose pathways, while infections stem from the immunosuppressive properties of these agents. Virtually all of the side effects associated with mTOR inhibitors can be managed effectively with dose modification and/or supportive intervention. The safety profile of everolimus observed in the phase III BOLERO-2 study is consistent with prior experience in the oncology setting; events are

predominantly low grade (grade 1 or 2). An increased risk of non-infectious pneumonitis, infection, and stomatitis in the everolimus plus exemestane arm relative to the control arm [exemestane + placebo] was observed, although each of these events can be effectively managed in this setting. The most common adverse events (AEs) suspected to be related to treatment, with an incidence ≥10%, reported in association with everolimus plus exemestane therapy were consistent with what was previously reported: stomatitis, rash, fatigue, decreased appetite, diarrhea, dysgeusia, nausea, pneumonitis, weight loss, anemia, epistaxis, hyperglycemia, thrombocytopenia, and pruritus. The most common grade 3-4 AEs suspected to be related to treatment with an incidence of ≥2% were: stomatitis, hyperglycemia, anemia, pneumonitis, fatigue, elevated alanine and aspartate transaminase concentrations, elevated γ-glutamyltransferase concentrations, dyspnea, neutropenia, and thrombocytopenia. No new safety concerns have emerged compared to previous experience with everolimus monotherapy or combination therapy.

Please refer to the Full Prescribing Information for everolimus for complete safety information: http://www.accessdata.fda.gov/scripts/cder/drugsatfda/.

2.4.3 Exemestane

Exemestane is an irreversible steroidal aromatase inhibitor that has demonstrated efficacy in the treatment of postmenopausal participants with advanced breast cancer. It is indicated for adjuvant treatment of postmenopausal women with ER+ early breast cancer who have received two to three years of tamoxifen and are switched to exemestane for completion of a total of five consecutive years of adjuvant hormonal therapy. As an off-label indication, it is also commonly given as adjuvant endocrine therapy in monotherapy for a total period of 5 years. It is further indicated for the treatment of advanced breast cancer in postmenopausal women whose disease has progressed following tamoxifen therapy. Exemestane is initially recognized by the aromatase enzyme as a false substrate and is then transformed through an NADPH-dependent mechanism to an intermediate that binds irreversibly to the enzyme, causing inactivation. Exemestane significantly lowers circulating estrogen concentrations (estradiol, estrone and estrone sulfate), but has no detectable effect on adrenal biosynthesis of corticosteroids or aldosterone (Aromasin prescribing information Pfizer-Pharmacia 2005). The recommended daily dose of exemestane is 25 mg via oral administration. Exemestane is rapidly absorbed from the gastrointestinal tract. Its bioavailability is limited by first-pass metabolism, but is increased when taken with food. Exemestane is widely distributed, and is extensively bound to plasma proteins. The terminal half-life for exemestane is 18-24 hours. The time needed to reach maximal E2 suppression is 7 days.[65–67] Exemestane is metabolized by CYP3A4 and aldoketoreductases. It does not inhibit any of the major CYP isoenzymes, including CYP 1A2, 2C9, 2D6, 2E1 and 3A4. Although no formal drug-drug interaction studies have been conducted, significant effects on exemestane clearance by CYP isoenzyme inhibitors appear unlikely (Aromasin prescribing information Pfizer-Pharmacia 2011).[67,68] As noted in Section 2.4.2.2, everolimus increases exposure of exemestane. The most frequently reported adverse effects for exemestane are gastrointestinal disturbances, hot flashes, arthralgia, myalgia, sweating, fatigue, and dizziness. Other reported effects include headache, insomnia, somnolence, depression, skin rashes, alopecia, asthenia, and peripheral and leg edema. Thrombocytopenia and leukopenia have been reported occasionally.

Reductions in bone mineral density can occur with long-term use of exemestane. A total of 1058 participants were treated with exemestane 25 mg once daily in the clinical trials program. Exemestane was generally well tolerated, and adverse events were usually mild to moderate. Adverse events occurring in greater than 10% of participants include hot flashes (14%), nausea (11.9%), insomnia, headache, increased sweating, joint and musculoskeletal pain, and fatigue (USPI Aromasin SmPC August 2008 [UK as RMS for EU MRP]). Androgenic effects were reported in a limited number of participants (4.3%).[67]

Please refer to the Full Prescribing Information for exemestane for complete safety information: http://www.accessdata.fda.gov/scripts/cder/drugsatfda/.

2.5 Study rationale

Endocrine therapy is the backbone therapy of ER-positive advanced breast cancer. In postmenopausal women, the use of NSAI is a common, effective and well established treatment option, being associated with good tolerability and clinical benefit.[69,70]

A recent phase II trial (PALOMA-2) further showed that the clinical benefit of NSAI monotherapy for metastatic ER-positive, HER2-negative BC can be additionally enhanced with the association of palbociclib, an oral, potent, CDK4/6 inhibitor.[31] With a median follow-up of almost 30 months, median progression free-survival (PFS) of the combination arm was of 20.2 month compared to 10.2 months in the letrozole monotherapy arm. Subsequently, a combination of palbociclib with fulvestrant further demonstrated improved outcomes in participants showing clinical resistance to an NSAI (PALOMA-3; either relapsing during or within 12 months after adjuvant endocrine therapy or progression on NSAI in the metastatic setting). At a preplanned interim analysis after 195 events of disease progression or death, median PFS was of 9.2 months with palbociclib in association with fulvestrant and 3.8 months with fulvestrant plus placebo. As a matter of fact, abnormalities that result in CDK activation and thus Cyclin D-CDK 4/6-Rb-E2F pathway activation are frequent in the luminal A and B molecularly defined breast cancer.[33] Unfortunately, despite the unprecedented benefit derived from the addition of palbociclib to letrozole in PALOMA-1, after 3 years of study enrollment, more than 70% of the participants had a disease progression event (either due to primary or secondary resistance). Similarly, in PALOMA-3, after 1 year of study enrollment more than 65% of the participants had a disease progression event. Therefore, improving disease control with endocrine therapy-based regimens is still an unmet medical need.

PI3K-AKT-mTOR pathway aberrations are a common molecular finding in breast cancer, being present in up to half of these tumors (the majority involving the PIK3CA gene, but also PTEN and AKT).[71] These aberrations have been identified as a mechanism of resistance to endocrine therapy.[15,16,72] In this context, everolimus, an agent from a class of drugs targeting mTOR (a downstream target of PI3K), demonstrated clinical benefit in those participants with HR-positive advanced breast cancer who were refractory to a NSAI (BOLERO-2).[19] In that study, the combination of everolimus plus exemestane was associated with a median progression-free survival of 10.6 months, which compared to a median PFS of 4.1 months with single agent exemestane (HR 0.36; 95% CI: 0.27 to 0.47; P<0.001).

The co-administration of agents targeting the PI3K/mTOR pathway and agents targeting the CDK4/6 pathway is still a relatively unexplored avenue, especially in the setting of progression to a previous CDK 4/6 inhibitor regimen and a prior NSAI in participants with advanced ER-positive Her2-negative BC. Remarkably, preclinical data showed that the addition of PI3K/mTOR pathway inhibitors to CDK4/6 inhibitors seems a promising strategy, both by increasing CDK 4/6 inhibitors lethality[73] or overcoming PI3K/mTOR pathway inhibitors resistance[74]. These data constitutes the clinical and preclinical motivation for this trial. Therefore, the purpose of this study is to evaluate the safety and tolerability of the investigational combination of palbociclib plus everolimus plus exemestane in postmenopausal women with ER+ Her2- metastatic breast cancer progressing to previous CDK 4/6 inhibitor and to an NSAI. Furthermore, this study will also evaluate the efficacy of this investigational combination as measured by its CBR.

2.6 Correlative Studies Background

In this study we plan to perform a correlative sub-study as detailed bellow. Technical details are further discussed in Section 9.

<u>The molecular underpinnings of CDK 4/6 inhibitors response and resistance</u>

Cyclin D is an essential element in the governance of the p16-cyclin D/CDK-Rb-E2F pathway, thus being an appealing target for cancer therapy.[75] As a matter of fact, strong preclinical[28,29] and clinical evidence[30–32,76] support the relevance of this target for cancer therapy, especially for luminal BC. Unfortunately, primary and secondary resistance to CDK4/6 inhibitors significantly hampers sustained treatment benefit, as demonstrated in two recent clinical trials: in PALOMA-2 (palbociclib plus letrozole as first line metastatic therapy), after 3 years of study enrollment, more than 70% of the participants had a disease progression event; similarly, in PALOMA-3 (palbociclib plus fulvestrant in participants refractory to NSAI), after 1 year of study enrollment more than 65% of the participants had a disease progression event.

To appropriately target CDK 4/6 inhibitors therapy and obviate the limitations of primary and secondary resistance, Finn and colleagues performed preclinical evaluations of palbociclib in a group of 44 human BC cell lines. This seminal study identified promising biomarkers to tailor palbociclib therapy, such as the ER.[29] Curiously, while palbociclib acted mostly as a cytostatic in ER+ cellular models, it had a synergistic cytotoxic activity when associated to tamoxifen. Furthermore, palbociclib was also able to partially revert tamoxifen resistance. In the same work, high levels of cyclin D1 and Rb and low levels of p16 were also associated with higher sensitivity to palbociclib. Also, while sensitive cell lines responded to palbociclib with an increase of phosphorylated Rb (“switching” Rb off), resistant cell lines did not reveal a change in Rb phosphorylation status. This fact constituted a strong proof of concept of the dependence of palbociclib action from the p16-cyclin D/CDK-Rb-E2F pathway. Such failure of palbociclib to dephosphorylate Rb in resistance cell lines could be due to several reasons, as mutations of CDK4/6, other mechanisms driving Rb hyperphosphorylation (as greater dependence on CDK1/2-cyclin E interactions) and/or loss of negative regulators of p16-cyclin D/CDK-Rb-E2F pathway.[29]

Building on the available preclinical evidence of efficacy and biomarkers, the palbociclib pivotal phase II trial presented a novel design.[31] In that trial, two independent cohorts accrued sequentially: cohort 1 composed of participants with ER+/HER2- breast cancers, and cohort 2 equally composed of participants with ER+/HER2- tumors, but also positive for cyclin D1 amplification, p16 loss, or both. Despite the selection of participants restricted to those with ER+ tumors (not allowing the comparison with other subtypes of breast cancer), the unprecedented benefit from palbociclib plus letrozole when compared to letrozole alone favors an especially high sensitivity of this BC subtype to CDK4/6 manipulation. Of note, the preclinical analysis from Finn and colleagues also suggested that ER+/HER2+ tumors might benefit from cyclin D-CDK4/6 inhibition with palbociclib, a hypothesis still being explored. Disappointingly, the enrichment in cohort 2 for participants with markers of anticipated benefit from palbociclib did not improve outcomes in the arm receiving palbociclib. Intriguingly, the arm of cohort 2 receiving letrozole as monotherapy presented remarkable outcomes.

On the other side of the coin are a group of BC features that render tumors resistant to palbociclib. Still on the basis of the seminal work by Finn and colleagues, non-luminal/basal intrinsic subtype breast cancer seem to be resistant to palbociclib[29], a feature that might be explained by the frequent loss of Rb in basal subtype BC.[77] As a matter of fact, G1/S phase transitions do not seem to be controlled by cyclin D1 in the absence of a functional Rb.[78] Dean and colleagues further extended this work body using a panel of Rb knockdown immortalized human breast epithelial cells and several BC cell lines.[79] In this cellular model, while Rb proficient cells treated with palbociclib had a strong decline of cell division, acute response to palbociclib was greatly impaired by the absence of Rb, which supports the relevance of Rb integrity for palbociclib response. Of note, palbociclib still exerted a partial cytostatic effect in cell lines deprived of Rb (even though lower than in Rb-proficient cells), challenging the idea that Rb is the only mediator of response to palbociclib. In this setting, an elevation of p107 levels was noted, and the authors propose that it may constitute a route for partial repression of E2F-target genes. Furthermore, cell lines resistance to palbociclib after chronic treatment with CDK4/6 inhibition, frequently presented increased levels of p107 and CDK2 protein and/or loss of p21 and p27.[79] Curiously, in this setting, loss of p21/p27 was due to a post-transcriptional mechanism (not at transcriptional level), a finding already documented in certain breast cancers, in which loss of p27 is associated with poor prognosis. As a consequence, the authors suggest that p27 loss could serve as a marker of both BC with poor prognosis and resistance to CDK 4/6 inhibition. Moreover, E2F overexpression led to a complete resistance to palbociclib, a phenomenon independent from Rb status.[79] Interestingly, in Rb proficient cells, forcing E2F overexpression led to Rb phosphorylation. Finally, palbociclib treatment might lead to cyclin D1 stabilization and accumulation (probably due to delayed turnover).[79] As a consequence, limiting doses of palbociclib could result in a rapid activation of CDK4/6. Finally, p53 may also play a relevant role as a marker of response to CDK4/6 inhibitors. Remarkably, p53 is a known regulator of CDK4[80,81], and interesting findings from ovarian and brainstem glioma models found that p53 mutation is correlated with CDK 4/6 inhibitors resistance.[82,83]

In this sub-study we aim to characterize the molecular underpinnings of CDK4/6 resistance using paired biopsies at baseline and disease progression, as successfully performed in other settings.[84–87] The evaluation will focus on the p16-cyclin D/CDK-Rb-E2F and p53 pathways to seek exploratory correlations with clinical response.

3. PARTICIPANT SELECTION

Participants must meet the following criteria on screening examination to be eligible to participate in the study. Laboratory tests required for eligibility must be completed within 14 days prior to the date of registration. Diagnostic tests, such as MRIs and CT scans, must be performed within 30 days of registration and baseline measurements must be documented within 14 days of the date of registration.

3.1 Inclusion Criteria

3.1.1 Participants with histologically or cytologically confirmed hormone receptor (HR)-positive, Her2-negative metastatic breast cancer. Central confirmation of HR positivity is not required

3.1.2 Postmenopausal women as defined as:

- Age >60 years

  or
- Age $\geq$45 with intact uterus and amenorrhea for $\geq$12 consecutive months or Follicle stimulating hormone (FSH) levels within postmenopausal range according to the ranges established by the testing facility

  or
- Premenopausal women who have been on a GnRH agonist for at least 6 weeks prior to study entry. Women in this group MUST remain on the GnRH agonist for the duration of protocol treatment

  or
- Status post bilateral oophorectomy, after adequate healing post surgery;

3.1.3 Men are eligible, as long as on a GnRH agonist for at least 6 weeks prior to study entry. Men MUST remain on the GnRH agonist for the **duration of protocol treatment.**

3.1.4 Participants must have measurable disease as per RECIST 1.1.

3.1.5 Prior Treatment Specifics:

- Participants must have radiological or objective evidence of progression to a CDK4/6 inhibitor regimen in the metastatic setting AND relapse/progression on an NSAI (defined as either relapsed ≤ 12 months after completing adjuvant NSAI or progressed through an NSAI for metastatic or locally advanced breast cancer)
- Participants may have received any number of previous endocrine/hormonal lines of therapy in the metastatic setting, as long none of them were exemestane-based and the last dose is ≥ 14 days prior to registration;
- Participants may have received up to one prior chemotherapy line for advanced breast cancer as long as the last dose is ≥ 21 days prior to registration;
- Participants may have received prior biologic treatments or investigational drugs as long as the last dose is ≥ 21 days prior to registration;
- Participants may have received radiotherapy for palliative purposes but must not be experiencing > grade 1 treatment related toxicies and have completed treatment ≥ 14 days prior to registration

3.1.6 Age ≥18 years. Age restriction applies given that no dosing or adverse event data are currently available on the use of palbociclib or exemestane in participants <18 years of age.

3.1.7 ECOG performance status ≤2 (Karnofsky ≥60%, see Appendix A)

3.1.8 For participants enrolling the phase IIa part of the study, accessible tumor lesion(s) for the purpose of research biopsy and willingness to undergo a research biopsy before treatment initiation and at the time of disease progression, as well as a single research blood sample before initiation of therapy. Participants who undergo an attempted on-treatment research biopsy and in whom inadequate tissue is obtained are still eligible to receive protocol therapy. They will not be required to undergo a repeat research biopsy attempt.

3.1.9 For participants enrolling the phase Ib part of the study, willingness to provide archival tumor samples when available.

3.1.10 Participants must have normal organ and marrow function, as defined below:

– absolute neutrophil count	≥1,5x10^9/L
– platelets	≥100 x10^9/L
– total hemoglobin	≥ 9 g/dL (which may be post transfusion)
– total bilirubin	≤1.5 x institutional upper limit of normal
– AST(SGOT)/ALT(SGPT)	≤2.5 × institutional upper limit of normal or ≤5 × institutional upper limit of normal for participants with liver ~~metastization~~metastases
– creatinine	≤1.5 x above institutional normal or ≥ 60 ml/min/1.73m^2 for subjects with creatinine levels above institutional normal.
– baseline QTc	≤ 500 ms

– fasting plasma glucose <140 mg/dL / 7.8 mmol/L

3.1.11 The effects of the combination of palbociclib, everolimus and exemestane on the developing human fetus are unknown. Given that women in this study will be post-menopausal by eligibility criteria (*de facto* or pharmacologically induced), it is expected that there will be no women of child-bearing potential in this study. If, for any reason, a woman should become pregnant or suspect that she is pregnant while participating in this study, she should inform her treating physician immediately. Of note, premenopausal women and men are only eligible if they have been on a GnRH agonist for at least 6 weeks prior to study entry. These participants MUST remain on the GnRH agonist for the duration of protocol treatment. Such participants should be counseled prior to study entry that GnRH agonists alone may not be adequate contraception and that adequate contraception (barrier method of birth control; abstinence) should be employed for the duration of study participation.

3.1.12 Ability to understand and the willingness to sign a written informed consent document.

3.2 Exclusion Criteria

3.2.1 Participants who have demonstrated intolerance to 125mg of Palbociclib are ineligible for the Phase I portion.

3.2.2 Participants who are receiving any other investigational agents.

3.2.3 Partcipants who have received previous treatment with an mTOR inhibitor or exemestane.

3.2.4 Participants with a history of allergic reactions attributed to compounds of similar chemical or biologic composition to palbociclib, everolimus or exemestane.

3.2.5 Participants with known brain metastases may be enrolled in this study if radiation therapy and/or surgery have been completed with a minimum of 3 months of stable disease demonstrated on serial evaluation by CT (with contrast enhancement) or MRI. Such participants must no longer require treatment with corticosteroids or enzyme inducing anti-epileptic medications for their CNS disease.

3.2.6 Participants with bilateral diffuse lymphangitic carcinomatosis.

3.2.7 Participants with significant symptomatic deterioration of lung function. If clinically indicated, pulmonary function tests including measures of predicted lung volumes, DLco, O_2 saturation at rest on room air should be considered to exclude restrictive pulmonary disease, pneumonitis or pulmonary infiltrates.

3.2.8 Evidence of current pneumonitis

3.2.9 Subjects with organ allograft requiring immunosuppression.

3.2.10 Participants with uncontrolled intercurrent illness including, but not limited to:

- Ongoing or active infection, symptomatic congestive heart failure, unstable angina pectoris, symptomatic cardiac arrhythmia, or psychiatric illness/social situations that would limit compliance with study requirements. Ability to comply with study requirements is to be assessed by each investigator at the time of screening for study participation;
- Impairment of gastrointestinal function or who have gastrointestinal disease that may significantly alter the absorption of study drugs (e.g., ulcerative disease, uncontrolled nausea, vomiting, diarrhea or malabsorption syndrome).

3.2.11 Participants receiving any medications or substances that are moderate or strong inhibitors or inducers of CYP3A within 7 days of registration. Lists including medications and substances known or with the potential to interact with the CYP3A isoenzymes are provided in Appendix B, and can also be found within Sections 2.4.1.2 and 5.8. Because the lists of these agents are constantly changing, it is important to regularly consult a frequently-updated list such as: http://medicine.iupui.edu/clinpharm/ddis/table.aspx; medical reference texts such as the Physicians' Desk Reference may also provide this information. As part of the enrollment/informed consent procedures, the participant will be counseled on the risk of interactions with other agents, and what to do if new medications need to be prescribed or if the participant is considering a new over-the-counter medicine or herbal product.

Proton pump inhibitors(PPI) may be taken while on study, however it is recommended that the PPI is taken 12 hours from the time of palbociclib administration. If needed, alternative antacid therapies may be used including H2-receptor antagonists and locally acting antacids. H2-receptor antagonists should be administered with a staggered dosing regimen (twice daily). The dosing of palbociclib should occur at least 10 hours after H2-receptor antagonist evening dose and 2 hours before the H2-receptor antagonist morning dose.

3.2.12 Pregnant women are excluded.

3.2.13 Individuals with a history of a different malignancy are ineligible except for the following circumstances: a) if they have been disease-free for at least 5 years and are deemed by the investigator to be at low risk for recurrence of that malignancy; b) if diagnosed with the following cancers and treated within the past 5 years: ductal carcinoma *in situ* of the breast, cervical cancer *in situ*, and basal cell or squamous cell carcinoma of the skin.

3.2.14 Participants known to be HIV-positive on combination antiretroviral therapy are ineligible because of the potential for pharmacokinetic interactions with palbociclib and everolimus. In addition, these individuals are at increased risk of lethal infections when treated with marrow-suppressive therapy. Appropriate studies will be undertaken in participants receiving combination antiretroviral therapy when indicated. Screening for HIV infection at baseline is not required.

3.3 Inclusion of Women and Minorities

Women are eligible for this protocol and expected to comprise the majority of participants enrolled. Every effort will be made to include participants from minority populations.

4. REGISTRATION PROCEDURES

4.1 General Guidelines for DF/HCC Institutions

Institutions will register eligible participants in the Clinical Trials Management System (CTMS) OnCore. Registrations must occur prior to the initiation of protocol therapy. Any participant not registered to the protocol before protocol therapy begins will be considered ineligible and registration will be denied.

An investigator will confirm eligibility criteria and a member of the study team will complete the protocol-specific eligibility checklist.

Following registration, participants may begin protocol therapy. Issues that would cause treatment delays should be discussed with the Overall Principal Investigator (PI). If a participant does not receive protocol therapy following registration, the participant’s registration on the study must be canceled. Registration cancellations must be made in OnCore as soon as possible.

4.2 Registration Process for DF/HCC Institutions

DF/HCC Standard Operating Procedure for Human Subject Research Titled *Subject Protocol Registration* (SOP #: REGIST-101) must be followed.

4.3 General Guidelines for Other Investigative Sites

N/A

4.4 Registration Process for Other Investigative Sites

N/A

5. TREATMENT PLAN

5.1 Treatment Regimen

This is a single center, single arm, open label, phase Ib/IIa study of the combination therapy with palbociclib, everolimus and exemestane in participants with metastatic HR-positive Her2-negative breast cancer. Each subject will be assigned a unique identification number during screening, which will be used on all case report forms (CRFs) and correspondence regarding the subject.

Palbociclib will be administered orally, once daily for 21 consecutive days followed by a 7-day rest (28-day cycle); everolimus and exemestane will be administered orally, once daily on a 28 day schedule (Table 5-1). Treatment will be administered on an outpatient basis. Reported adverse events and potential risks are described in Section 7. Appropriate dose modifications are described in Section 6. No investigational or commercial agents or therapies other than those described below may be administered with the intent to treat the participant's malignancy.

Table 5-1 – Regimen description

Agent	Premedication Precautions	Dose	Route	Schedule	Cycle Length
Palbociclib	Not routinely necessary	**	PO	Days 1 to 21 followed by a 7-day rest	28 days (4 weeks)
Everolimus	Not routinely necessary	**	PO	Days 1 to 28	
Exemestane	Not routinely necessary	25 mg	PO	Days 1 to 28	
***Doses as appropriate for assigned dose level*					

Participants will be treated with increasing/decreasing doses of palbociclib and everolimus to establish MTD(s)/RP2D for both drugs in the setting of the triple combination of palbociclib, everolimus and exemestane. The starting dose level will be palbociclib 100 mg (PO, days 1 – 21 with 7 days of rest), everolimus 5 mg (PO, daily) and exemestane 25 mg (PO, daily). Only one of the two study drugs in the palbociclib-everolimus-exemestane combination can be escalated at a time. If participants develop toxicity early in the study, the everolimus dose can be de-escalated below the proposed starting dose. If subsequent cohorts of participants tolerate therapy, doses for participants on lower dose-levels can then be escalated. However, palbociclib starting doses below 100 mg will not be explored in this study (Table 5-2).

Table 5-2 - Provisional dose levels for palbociclib and everolimus for phase Ib

Dose Escalation Schedule			
Dose Level	Dose		
	Palbociclib (mg)	Everolimus (mg)	Exemestane (mg)
Level -1	100	2.5	25

**Level 0 – Starting dose**	**100**	**5**	**25**
Level 1	125	5	25
Level 2	125	10	25

The dose for subsequent dose levels will be determined according to the treatment-related adverse events observed in the first cycle, as detailed in Table 5-3. Participants must complete a minimum of ≥16 doses of palbociclib during within the minimum safety evaluation period (1 cycle, i.e. 28 days) or have had a DLT within the first cycle of treatment to be considered evaluable for dose escalation decisions. The participant will be requested to maintain a medication diary of each dose of medication. The medication diary will be returned to clinic staff at the end of each cycle.

Table 5-3 – Escalation decision rules

**Number of Participants with DLT at a Given Dose Level**	**Escalation Decision Rule**
0 out of 3	Enter 3 participants at the next dose level.
1 out of 3	Enter at least 3 more participants at this dose level.<br>• If 0 of these 3 additional participants experience DLT, proceed to the next dose level.<br>• If 1 or more of these additional 3 participants suffer DLT, then dose escalation is stopped. This dose is declared the maximally administered dose. Three (3) additional participants will be entered at the next lowest dose level if only 3 participants were treated previously at that dose.
≥2 out of 3	Dose escalation will be stopped. This dose level will be declared the maximally administered dose. Three (3) additional participants will be entered at the next lowest dose level if only 3 participants were treated previously at that dose.
≤1 out of 6 at highest dose level below the maximally administered dose	This is generally the recommended phase 2 dose. At least 6 participants must be treated at the recommended phase 2 dose.

Dose-limiting toxicity (DLT) for this trial are defined below. Dose escalation will continue until identification of the maximum tolerated doses (MTD) or a suitable combination of lower doses for Phase II recommended dose (RP2D).

5.2 Definition of Dose-Limiting Toxicity (DLT)

A dose-limiting toxicity (DLT) is defined as an adverse event or abnormal laboratory value assessed as having a reasonably possible relationship to the study medication(s) and is unrelated to disease, disease progression, inter-current illness, or concomitant medications that occurs

within the first 28 days of treatment (cycle 1) with palbociclib + everolimus + exemestane and meets any of the criteria included in Table 5-4. The National Cancer Institute Common Terminology Criteria for Adverse events (NCI CTCAE) version 4.0 will be used for all grading. Whenever a participant experiences toxicity that fulfills the criteria for a DLT, treatment with the study drug combination will be interrupted and the toxicity will be followed as described in Section 6.

For the purposes of dose escalation and determination of the MTD(s), DLTs that occur during the first cycle will be necessarily considered, including those in which the event started in Cycle 1 but confirmation of the DLT occurs in a subsequent cycle. The investigator must notify Pfizer immediately of any unexpected CTCAE grade ≥3 adverse events or laboratory abnormalities during the DLT period (first 28 days of treatment). Prior to enrolling participants into a higher dose level, CTCAE grade ≥2 adverse events will be reviewed for all participants at the current dose level.
Recommended guidelines for prophylactic or supportive treatment for expected toxicities, including management of study drug induced adverse events, are provided in Section 6. For participants who do not tolerate the protocol-specified dosing schedule, dose adjustments are permitted in order to allow the participant to continue the study treatment. These changes must be recorded on the CRF.

Table 5-4 – Study DLT description using the National Cancer Institute Common Terminology Criteria for Adverse events (NCI CTCAE) version 4.03

**Toxicity**	**DLT criteria**
Hematology	o CTCAE grade 3 thrombocytopenia with bleeding<br>o CTCAE Grade 3 or 4 neutropenia with fever (temperature ≥38.5°C)<br>o CTCAE grade 4 neutropenia lasting more than 7 consecutive days<br>o CTCAE grade 4 thrombocytopenia<br>o CTCAE grade 4 lymphopenia lasting more than 7 consecutive days
Gastro-intestinal	o ≥CTCAE grade 3 nausea or vomiting ≥48 hrs despite optimal anti-emetic therapy<br>≥CTCAE grade 3 diarrhea ≥48 hrs despite optimal anti-diarrhea treatment
Hepato-biliary	o ≥CTCAE grade 3 total bilirubin<br>≥CTCAE grade 3 ALT (isolated increases in AST without concomitant increases in ALT will not be considered dose-limiting, because of the non-specific nature of AST) <u>or</u> >7.5 x ULN for >72 hours in a participant with liver

	metastases<br>o ≥CTCAE grade 3 ALT and total bilirubin ≥2x ULN (Hy's Law case) where this is felt to be drug-related: discontinue study treatment
ECG QT Interval	o QTcF interval ≥501 ms on at least two separate ECGs
Renal	o CTCAE grade ≥3 serum creatinine
Other events not described above or listed as exceptions to DLT criteria	o ≥CTCAE grade 3 events
Exceptions to DLT criteria	o < 5 days of CTCAE grade 3 fatigue<br>o < 48 hours of CTCAE grade 3 edema<br>o Grade 3 laboratory abnormalities that are responsive to oral supplementation or deemed by the investigator to be clinically insignificant

5.3 Definition of maximum tolerated dose (MTD)

The MTD is defined as the highest combination drug dosage not causing medically unacceptable, dose-limiting toxicity (DLT) in more than 33% of the treated participants in the first cycle of treatment. AEs and laboratory abnormalities considered to be DLTs are defined in Table 5-4. Since several combinations may correspond to this definition, more than one MTD may be identified with different doses of the combination partners. One of these MTDs or a lower dose combination will then be selected as the recommended phase II dose (RP2D). Only one RP2D will be tested in the phase II part of the study.

5.4 Intra-participant dose escalation

Intra-participant dose escalation of palbociclib prior to declaration of MTD(s)/RP2D is not permitted with the following exception:

- Once the MTD(s)/RP2D are/is declared for the palbociclib-everolimus-exemestane combination, individual participants may be considered for escalation to treatment at the RP2D. In order for a participant to be treated at the RP2D, the participant must have tolerated the lower dose combination for at least two cycles of therapy, i.e., the participant must not have experienced at the lower dose combination originally assigned a toxicity of CTCAE Grade ≥2 for which relationship to study drug cannot be ruled out.

These changes must be recorded on the Dosage Administration Record CRF.

5.5 Doses for Phase II

The doses of palbociclib-everolimus-exemestane combination are fixed as recommended by the Phase Ib part of this study. Only one RP2D will be tested in Phase II.

5.6 Pre-Treatment Criteria

5.6.1 Day -21 to Day 1: Screening Visit

Archival tumor samples (when available) will also be requested prior to registration.

Phase II: A baseline tumor biopsy, obtained within prior to starting protocol therapy is also required for participants with accessible disease. Further details about collection and handling of tumor biopsy specimen can be found in Section 9.1.

5.6.2 Cycle 1, Day 1 and subsequent cycles

Participants do not need to re-meet eligibility criteria on Cycle 1 Day 1.
Treatment criteria for Cycle 1 Day1, subsequent cycles Day 1, and post-treatment interruptions for treatment-related toxicity includes:

- Platelet count ≥ 100,000/mm^3; and
- ANC ≥ 1000/mm^3 and no fever; and
- To resume palbociclib, any grade 3 or higher treatment-related non-hematologic adverse events (AEs) considered related to palbociclib have recovered to Grade ≤ 1 or baseline. To resume everolimus, any grade 3 or higher treatment-related non-hematologic adverse events (AEs) considered related to everolimus have recovered to Grade ≤ 1 or baseline. AE grading criteria can be found online at: http://ctep.cancer.gov/protocolDevelopment/electronic_applications/ctc.htm.

If the retreatment parameters are met within 3 weeks of treatment interruption or cycle delay, palbociclib and/or everolimus may be resumed. Please refer to Dose Reductions Section (Section 6) for detailed adverse event and dose modifications information

5.7 Agent Administration

5.7.1 Palbociclib

Palbociclib should be taken orally, once per day within 1 hour of a meal for 21 consecutive days followed by a 7-day rest (28-day cycle). If a dose is vomited, a replacement dose should NOT be taken. If a dose is missed (defined as 4hours from regularly scheduled dose time), it should be skipped and NOT retaken. Participants who inadvertently take 1 extra dose during a day must skip the next day's dose. Participants should be instructed to swallow the palbociclib capsule whole and not to chew, crush or open them. Participants must avoid consumption of grapefruit. Participants should be instructed to record daily administration of the study drugs in a drug diary (Appendix C). Treatment is continuous daily for 21 days, and then 7 days off, to complete a 28 day cycle.

In addition, participants will bring pill bottles to visits, and pill counts will be performed as follows:

- Participants will be required to return all bottles of palbociclib as well as the completed drug diary at each study visit for drug accountability. Drug accountability will be performed at each study visit. The number of remaining capsules/tablets will be documented and recorded.
- To be considered evaluable, each study participant must have received at least ≥16 doses of palbociclib during the cycle one.

5.7.2 Everolimus

Everolimus should be taken orally, once per day within 1 hour of a meal. If a dose is vomited, a replacement dose should NOT be taken. If a dose is missed (defined as 4hours from regularly scheduled dose time), it should be skipped and NOT made-up. Participants who inadvertently take 1 extra dose during a day must skip the next day's dose. Participants should be instructed to swallow the everolimus tablet whole and not to chew or crush it. Participants must avoid consumption of grapefruit. Participants should be instructed to record daily administration of the study drugs in a drug diary (Appendix C). Treatment is continuous daily to complete a 28 day cycle.

- Participants will be required to return the completed drug diary at each study visit to assess drug adherence.

5.7.3 Exemestane

Exemestane should be taken orally, once per day within 1 hour of a meal. If a dose is vomited, a replacement dose should NOT be taken. If a dose is missed (defined as 4hours from regularly scheduled dose time), it should be skipped and NOT made-up. Participants who inadvertently take 1 extra dose during a day must skip the next day's dose. Participants should be instructed to swallow the exemestane tablet whole and not to chew or crush it. Participants should be instructed to record daily administration of the study drugs in a drug diary (Appendix C). Treatment is continuous daily to complete a 28 day cycle.

In addition, participants will bring pill bottles to visits, and pill counts will be performed as follows:

- Participants will be required to return all bottles of exemestane as well as the completed drug diary at each study visit for drug accountability. Drug accountability will be performed at each study visit prior to dispensing drug supply for the next cycle. The number of remaining capsules/tablets will be documented and recorded.
- To be considered compliant, each study participant must have received at least 80% of the planned number of doses of primary therapy based on the number of days of actual dose administration.

5.8 General Concomitant Medication and Supportive Care Guidelines

All prior treatment or medication administered during the 30 days preceding the first dose of study treatment and any concomitant therapy administered to the subject throughout the study until 30 days after the final dose of study treatment must be documented in the participant's

medical record or research chart.

Supportive care medications are allowed at any time on trial. Specifically, the following agents are permitted:

- Antiemetics;
- Antidiarrheal therapy and topical treatments for stomatitis;
- Antiallergic measures such as corticosteroids and antihistamines. Chronic immunosuppressive therapies should be avoided, including systemic corticosteroids.
- Bisphosphonates: Subjects being treated with bisphosphonates when they enter the study may continue the medication as long as the dose is stable. Subjects may also initiate bisphosphonate therapy while on protocol therapy if it is thought to be medically necessary. The reason for bisphosphonates initiation during after study enrollment must be clearly specified;
- Agents to assist in management of AI-induced side effects (NSAIDs, gabapentin, duloxetine, venlafaxine, citalopram, etc);
- Pain killers;
- Packed red blood cells (with the exception of cycle 1);
- Concurrent metformin is allowable;
- The use of concurrent investigational or other antitumor therapies, other than everolimus and exemestane, is not permitted;
- The use of any other potential new concomitant medications may be discussed between the investigator and the sponsor on a case by case basis.

Prophylactic myeloid growth factor administration is not permitted for the prevention of neutropenia, but is only permitted if clinically indicated in response to a clinically significant episode of neutropenia

Participants will be required to tell their treating physican/research nurse about any new medications he/she takes after the start of the study drug combination.

Participants taking concomitant medication chronically should be maintained on the same dose and dose schedule throughout the study period, as medically feasible. The days of full PK blood sampling should be representative of the other study days with regard to the use of the chronically administered concomitant medications. However, if a concomitant medication is used intermittently during the study, this medication should be avoided on the days of full PK sampling, if medically feasible. The use of herbal medicine is not recommended during protocol treatment.

Moderate and strong CYP3A inhibitors/inducers are not allowed on study. Palbociclib is metabolized to multiple metabolites in a qualitatively similar manner in rat, dog and human liver microsomes. In vitro, Palbociclib is primarily metabolized by CYP3A4 enzymes. Co-administration with drugs that are CYP3A inhibitors and inducers may change the plasma concentrations of palbociclib in humans. The concurrent use of CYP3A inhibitors, including amprenavir, aprepitant, atazanavir, boceprevir, clarithromycin, conivaptan, delavirdine, diltiazem, erythromycin, fluconzole, fosamprenavir, indinavir, itraconazole, ketoconazole, lopinavir, mibefradil, miconazole, nefazodone, nelfinavir, posaconazole, ritonavir, saquinavir,

telaprevir, telithromycin, verapamil, voriconazole, and grapefruit, grapefruit juice or any product containing grapefruit, are not allowed in the study. The concurrent use of CYP3A inducers, including carbamazepine, felbamate, nevirapine, phenobarbital, phenytoin, primidone, rifabutin, rifampin, rifapentin, and St. John's wort, are not allowed in the study. This medication list may also be found in Appendix B. Concomitant use of moderate CYP3A inducers and CYP3A substrates is allowable on study, however precaution should be exercised for use of any concomitant medication.

Proton pump inhibitors(PPI) may be taken while on study, however it is recommended that the PPI is taken 12 hours from the time of palbociclib administration. If needed, alternative antacid therapies may be used including H2-receptor antagonists and locally acting antacids. H2-receptor antagonists should be administered with a staggered dosing regimen (twice daily). The dosing of palbociclib should occur at least 10 hours after H2-receptor antagonist evening dose and 2 hours before the H2-receptor antagonist morning dose. Local antacid should be given at least 2 hours before or after palbociclib administration.

Appendix B, palbociclib package insert, and everolimus package insert present information on medications/substances that could potentially interact with the study agents.

5.9 Criteria for Taking a Participant Off Protocol Therapy

Duration of therapy will depend on individual response, evidence of disease progression and tolerance. In the absence of treatment delays due to adverse event(s), treatment may continue until one of the following criteria applies:

- Disease progression;
- Intercurrent illness that prevents further administration of treatment;
- Unacceptable adverse event(s);
- Participant demonstrates an inability or unwillingness to comply with the oral medication regimen and/or documentation requirements;
- Participant decides to withdraw from the protocol therapy;
- General or specific changes in the participant's condition render the participant unacceptable for further treatment in the judgment of the treating investigator.

Participants will be removed from the protocol therapy when any of these criteria apply. The reason for removal from protocol therapy, and the date the participant was removed, must be documented in the CRF. Alternative care options will be discussed with the participant.

Additionally the date and reason the patient was removed from protocol therapy will be documented in OnCore by the study staff.

5.10 Duration of Follow Up

Participants who are removed from study therapy for disease progression will be followed for 30 days or until death, whichever occurs first.

Participants who are alive and free of disease progression at the time of removal from protocol therapy will be followed until disease progression or until death, whichever occurs first. During the follow-up period, scans should be performed every 8 weeks +/- 2 weeks (if the participants is within 24 weeks of initiation of study treatment) or every 12 weeks +/- 2 weeks (if the participant is greater than 24 weeks from initiation of study treatment) to evaluate for disease progression.

Participants removed from protocol therapy for unacceptable adverse event(s) will be followed until resolution or stabilization of the adverse event.

5.11 Criteria for Taking a Participant Off Study

Participants will be removed from study when any of the following criteria apply:

- Lost to follow-up
- Withdrawal of consent for data submission
- Progressive Disease for participants removed from protocol therapy for reasons other than disease progression
- Death

The reason for taking a participant off study, and the date the participant was removed, must be documented in the case report form (CRF) and in OnCore by study staff.

In the event of unusual or life-threatening complications, treating investigators must immediately notify the Overall PI, Sara Tolaney, MD at 617-632-5743 or stolaney@partners.org.

6. DOSING DELAYS/DOSE MODIFICATIONS

If a participant experiences a DLT, then treatment with all drugs must be interrupted (palbociclib, everolimus and exemestane). For all toxicity grades and with the exceptions noted from Table 6-3 to Table 6-11, if the toxicity resolves to grade 1 or baseline within 1 week of onset, treatment may be resumed at the same or a lower dose level at the investigator's discretion and following discussion with the overall study PI. If dosing is delayed for toxicities on day 1 of a cycle, the cycle start and all assessments will be delayed. If dosing is interrupted mid-cycle, the next cycle and all assessments will stay on schedule. For toxicities that result in treatment delays of more than seven but not more than 21 days, treatment may be resumed at a lower dose level. If a participant requires a dose interruption of > 21 days from the intended day of the next scheduled dose, then the participant must be discontinued from the study. In this event, more frequent follow up may be appropriate. All participants will be followed for AEs and for SAEs for 30 days following the last dose of study drug.

Dose delays and modifications will be made as indicated in the following tables. The descriptions and grading scales found in the revised NCI Common Terminology Criteria for Adverse Events (CTCAE) version 4.0 will be utilized for dose delays and dose modifications. A copy of the CTCAE version 4.0 can be downloaded from the CTEP website
http://ctep.cancer.gov/protocolDevelopment/electronic_applications/ctc.htm.

Palbociclib

Each participant is allowed a maximum of two dose reductions for palbociclib (Available doses in Table 6-1). After this, the participant will be discontinued from the study treatment. For each participant, once a dose level reduction for palbociclib has occurred, the dose may not be re-escalated during subsequent treatment cycles unless approved by the overall study PI.

Table 6-1 – Available dose reductions of palbociclib

Dose Reduction Steps	Palbociclib Dose
0	125 mg, daily
-1	100 mg, daily
-2	75 mg, daily
	Discontinue study treatment

Everolimus

Management of severe or intolerable adverse reactions may require temporary dose reduction and/or interruption of everolimus therapy. If dose reduction is required, the suggested dose is approximately 50% lower than the daily dose previously administered.

Available doses of everolimus are presented in Table 6-2. Recommendations for dose reduction, interruption or discontinuation of everolimus in the management of adverse reactions are summarized from Table 6-3 to Table 6-11. Clinical judgment of the treating physician should guide the management plan of each participant based on individual benefit/risk assessment.

Table 6-2 – Available dose reductions of everolimus

Dose Reduction Steps	Everolimus Dose
0	10 mg, daily
-1	5 mg, daily
-2	2.5 mg daily
	Discontinue study treatment

Exemestane

No specific dose modifications are recommended for exemestane. Toxicities attributed to exemestane should be managed in a manner consistent with the investigator's usual clinical practice. Dose modification guidelines are presented in the following tables:

Table 6-3 – Management of nausea, vomiting and diarrhea with maximal prophylaxis

Nausea, vomiting and diarrhea	Management/Next Dose for palbociclib	Management/Next Dose for everolimus
Grade 1	No change in dose.	
Grade 2	No change in dose.	
Grade 3	Withhold palbociclib and everolimus until it resolves to ≤ Grade 1. Restart at a lower dose.	
Grade 4	Off protocol therapy.	
Recommended management: antiemetics and antidiarrheal agents. Loperamide antidiarrheal therapy dosage schedule: 4 mg at first onset, followed by 2 mg with each loose motion until diarrhea-free for 12 hours (maximum dosage: 16 mg/24 hours); Adjunct anti-diarrheal therapy is permitted and should be recorded when used.		

Table 6-4 – Management of neutropenia and thrombocytopenia

Neutropenia and thrombocytopenia	Management/Next Dose for palbociclib	Management/Next Dose for everolimus
Grade 1	No change in dose.	
Grade 2	No change in dose.	
Grade 3: ANC <1.0 x 10^9/L with and without fever (≥38.5°C)	Withhold* palbociclib and everolimus until ANC is ≥1.0 x 10^9/L and participant is afebrile	
Grade 4: < 500/mm3 or < 0.5 x 10^9 /L; without fever and/or platelets < 75 x 10^9/L	Withhold* palbociclib and everolimus until ANC is ≥1.0 x 10^9/L and the platelet count is ≥75 x 10^9/L.	
*If treatment delay is ≤7 days, restart at same dose for both. If treatment delay is > 7 days but ≤21 days, restart at lower dose for both. If treatment delay is > 21 days, discontinue study treatment.<br>Participants requiring > two dose reductions should go off protocol therapy.		

Table 6-5 – Management of non-infectious pneumonitis

Non-infectious pneumonitis	Management/Next Dose for palbociclib	Management/Next Dose for everolimus
Grade 1: Asymptomatic, radiographic findings only.	No change in dose. Initiate appropriate monitoring.	
Grade 2: Symptomatic, not interfering with ADL	Continue at assigned dose.	Consider interruption of therapy. Re-initiate everolimus at a lower dose.<br><br>Rule out infection and consider treatment with corticosteroids until symptoms improve to ≤grade 1.<br><br>Discontinue treatment if failure to recover within 4 wks.
Grade 3: Symptomatic, interfering with ADL; O_2 indicated	Continue at assigned dose.	Interrupt until symptoms resolve to ≤Grade 1.<br><br>Rule out infection, and consider treatment with corticosteroids.<br><br>Consider re-initiating everolimus at a lower dose. If toxicity recurs at grade 3, consider discontinuation.
Grade 4: Life-threatening; ventilatory support indicated	Discontinue study treatment, rule out infection, and consider treatment with corticosteroids.	
ADL - Activities of daily living.		

Table 6-6 – Management of stomatitis

Stomatitis	Management/Next Dose for palbociclib	Management/Next Dose for everolimus
Grade 1: Minimal symptoms, normal diet	No dose adjustment required.<br>Manage with non-alcoholic mouth wash, salt water (0.9%) mouth wash, or decadron	

Stomatitis	Management/Next Dose for palbociclib	Management/Next Dose for everolimus
	mouth rinse several times a day.	
Grade 2: Symptomatic but can eat and swallow modified diet	Interrupt palbociclib and everolimus until recovery to grade ≤1.<br>Re-initiate both drugs at the same dose. If stomatitis recurs, interrupt dose of both drugs until recovery to grade ≤1. Re-initiate both drugs at a lower dose.<br>Manage with decadron mouth rinse, topical analgesic mouth treatments (e.g. benzocaine, butyl minobenzoate, tetracaine hydrochloride, menthol or phenol) with or without topical corticosteroids (i.e. triamcinolone oral paste).	
Grade 3: Symptomatic and unable to adequately aliment or hydrate orally	Interrupt palbociclib and everolimus until recovery to grade ≤1.<br>Re-initiate both drugs at a reduced dose.<br>Manage with topical analgesic mouth treatments (i.e. benzocaine, butyl minobenzoate, tetracaine hydrochloride, menthol or phenol) with or without topical corticosteroids (i.e. triamcinolone oral paste).	
Grade 4: Symptoms associated with life-threatening consequences	Discontinue study treatment and provide appropriate medical therapy.	

Table 6-7 – Management of QTc prolongation

QTc prolongation	Management/Next Dose for palbociclib	Management/Next Dose for everolimus
Grade 1	No change in dose	
Grade 2	No change in dose	
Grade 3	Withhold palbociclib and perform frequent ECGs until the QTcF is < 500 msec. Address electrolyte, calcium and magnesium abnormalities. Restart palbociclib.<br><br>If findings recur, hold palbociclib until resolve to ≤Grade 1, and restart at the next lower dose level. If findings recur again, discontinue palbociclib.	Continue with assigned dose.
Grade 4	Stop palbociclib, institute emergency care, address electrolyte, calcium and magnesium abnormalities and perform frequent ECGs until the QTcF is < 500 msec.<br><br>Discontinue the participant from the study.	Discontinue the participant from the study.

Table 6-8 – Management of metabolic events

Metabolic events (e.g. hyperglycemia, dyslipidemia)	Management/Next Dose for palbociclib	Management/Next Dose for everolimus
Grade 1	No dose adjustment required.<br>Initiate appropriate medical therapy and monitor.	
Grade 2	No dose adjustment required.<br>Manage with appropriate medical therapy and monitor.	
Grade 3	Continue palbociclib at assigned dose.	Temporary dose interruption of

**Metabolic events (e.g. hyperglycemia, dyslipidemia)**	**Management/Next Dose for palbociclib**	**Management/Next Dose for everolimus**
		everolimus.<br><br>Re-initiate everolimus at a lower dose.<br><br>Manage with appropriate medical therapy and monitor.
Grade 4	Continue palbociclib at assigned dose.	Discontinue everolimus and treat with appropriate medical therapy.

Table 6-9 – Management of hepatobiliary toxicity for participants without liver metastases

**Hepatobiliary**	**Management/Next Dose for palbociclib**	**Management/Next Dose for everolimus**
≥ Grade 3	Withhold study treatment until toxicity improves to ≤Grade 1.<br>Restart at a lower dose for both compounds	

Table 6-10 – Management of hepatobiliary toxicity for participants with liver metastases

**Hepatobiliary**	**Management/Next Dose for palbociclib**	**Management/Next Dose for everolimus**
>7.5 x ULN for >72 hours	Withhold study treatment until toxicity improves to ≤5 × institutional upper limit of normal.<br>Restart at a lower dose for both compounds	

Table 6-11 – Management of all other toxicities

**All other toxicities**	**Management/Next Dose for palbociclib**	**Management/Next Dose for everolimus**
≥ Grade 3	Determine attribution of toxicity. Hold assigned therapy until resolved to ≤Grade 1 or baseline. If treatment delay is >7 days but ≤21 days, restart at lower dose level. If treatment delay >21 days, discontinue study treatment.	

7. ADVERSE EVENTS: LIST AND REPORTING REQUIREMENTS

Adverse event (AE) monitoring and reporting is a routine part of every clinical trial. The following list of reported and/or potential AEs (Section 7.1) and the characteristics of an observed AE (Section 7.2) will determine whether the event requires expedited reporting **in addition** to routine reporting.

7.1 Expected toxicities

7.1.1 Adverse Events for Palbociclib

The primary anticipated toxicity of palbociclib is neutropenia. In the phase I, dose-escalation trial of palbociclib alone in advanced cancers[88], neutropenia was the only dose-limiting toxicity (DLT). Grade 3 neutropenia during cycle 1 was observed in 3/22 participants receiving palbociclib 125 mg PO daily, with no grade 4 neutropenia events observed. Based on this result, 125 mg PO daily became the recommended phase 2 dose (RP2D). Other hematologic AEs of grade 3 or greater during cycle 1 were anemia and leukopenia, occurring in 1 and 4 of 41 participants, respectively. The most common non-hematologic AEs of grade 3 or greater during cycle 1 were fatigue, nausea, and abdominal pain (each occurring in 2 of 41 participants). Of note, there were no complicated hematologic AEs documented, and all hematologic AEs resolved during the off-drug period of a 3 week on/1 week off schedule, and were non-cumulative.

In a phase II trial of palbociclib alone for advanced breast cancer, the only toxicities ≥ grade 3 observed were transient neutropenia (50%) and thrombocytopenia (21%).[52] In a phase II trial of palbociclib plus letrozole for first-line therapy of hormone receptor positive breast cancer, the most common AEs reported were neutropenia, leukopenia, and fatigue.[53,89] The median time to first treatment delay for neutropenia was 58 days, and the median duration of treatment delay until recovery was 5 days (range 1-16 days). (Pfizer internal data). In general, hematologic abnormalities were adequately managed with standard supportive care, were not complicated, and resolved during the drug hold with no cumulative toxicity noted.

In the phase I, dose-escalation trial of palbociclib alone in advanced cancers[88], QT interval changes were also evaluated in detail. While 26 of 41 participants had a maximum increase of <30 msec from baseline QTc, zero participants had an on-treatment value exceeding 500 msec.

7.1.2 Adverse Events for Everolimus

The following adverse events are considered to be class-effects of mTOR inhibitors: stomatitis/oral mucositis/ulcers, infections and infestations, rash and similar events, cytopenia, hemorrhages, non-infectious pneumonitis, hyperglycemia/new-onset diabetes mellitus, renal events, and thromboembolism. The more common metabolic side effects reported with mTOR inhibitors result from inhibitory effects on mTOR-regulated lipid and glucose pathways, while infections stem from the immunosuppressive properties of these agents. Virtually all of the side effects associated with mTOR inhibitors can be managed effectively with dose modification and/or supportive intervention. The safety profile of everolimus observed in the phase III BOLERO-2 study is consistent with prior experience in the oncology setting; events are predominantly low grade (grade 1 or 2). An increased risk of non-infectious pneumonitis, infection, and stomatitis in the everolimus plus exemestane arm relative to the control arm [exemestane + placebo] was observed, although each of these events can be effectively managed in this setting. The most common adverse events (AEs) suspected to be related to treatment, with an incidence ≥10%, reported in association with everolimus plus exemestane therapy were consistent with what was previously reported: stomatitis, rash, fatigue, decreased appetite, diarrhea, dysgeusia, nausea, pneumonitis, weight loss, anemia, epistaxis, hyperglycemia, thrombocytopenia, and pruritus. The most common grade 3-4 AEs suspected to be related to

treatment with an incidence of ≥2% were: stomatitis, hyperglycemia, anemia, pneumonitis, fatigue, elevated alanine and aspartate transaminase concentrations, elevated γ-glutamyltransferase concentrations, dyspnea, neutropenia, and thrombocytopenia. No new safety concerns have emerged compared to previous experience with everolimus monotherapy or combination therapy.

Please refer to the Full Prescribing Information for everolimus for complete safety information: http://www.accessdata.fda.gov/scripts/cder/drugsatfda/.

7.1.3 Adverse Events for Exemestane

The most frequently reported adverse effects for exemestane are gastrointestinal disturbances, hot flashes, arthralgia, myalgia, sweating, fatigue, and dizziness. Other reported effects include headache, insomnia, somnolence, depression, skin rashes, alopecia, asthenia, and peripheral and leg edema. Thrombocytopenia and leukopenia have been reported occasionally. Reductions in bone mineral density can occur with long-term use of exemestane. A total of 1058 participants were treated with exemestane 25 mg once daily in the clinical trials program. Exemestane was generally well tolerated, and adverse events were usually mild to moderate. Adverse events occurring in greater than 10% of participants include hot flashes (14%), nausea (11.9%), insomnia, headache, increased sweating, joint and musculoskeletal pain, and fatigue (USPI Aromasin SmPC August 2008 [UK as RMS for EU MRP]). Androgenic effects were reported in a limited number of participants (4.3%).[67]

Please refer to the Full Prescribing Information for exemestane for complete safety information: http://www.accessdata.fda.gov/scripts/cder/drugsatfda/.

7.2 Adverse Event Characteristics

- **CTCAE term (AE description) and grade:** The descriptions and grading scales found in the revised NCI Common Terminology Criteria for Adverse Events (CTCAE) version 4.0 will be utilized for AE reporting. All appropriate treatment areas should have access to a copy of the CTCAE version 4.0.

  A copy of the CTCAE version 4.0 can be downloaded from the CTEP web site http://ctep.cancer.gov/protocolDevelopment/electronic_applications/ctc.htm.

- **For expedited reporting purposes only:**
  - AEs for the agent(s) that are listed above should be reported only if the adverse event varies in nature, intensity or frequency from the expected toxicity information which is provided.

- **Attribution** of the AE:
  - Definite – The AE is clearly related to the study treatment.
  - Probable – The AE is likely related to the study treatment.
  - Possible – The AE may be related to the study treatment.
  - Unlikely – The AE is doubtfully related to the study treatment.

- Unrelated – The AE is clearly NOT related to the study treatment.

7.3 Expedited Adverse Event Reporting

7.3.1 Investigators must report to the Overall PI any serious adverse event (SAE) that occurs after the initial dose of study treatment, during treatment, or within 30 days of the last dose of treatment on the local institutional SAE form.

7.3.2 DF/HCC Expedited Reporting Guidelines

Investigative sites within DF/HCC will report AEs directly to the DFCI Office for Human Research Studies (OHRS) per the DFCI IRB reporting policy.

7.4 Expedited Reporting to the Food and Drug Administration (FDA)

The Overall PI, as study sponsor, will be responsible for all communications with the FDA. The Overall PI will report to the FDA, regardless of the site of occurrence, any serious adverse event that meets the FDA's criteria for expedited reporting following the reporting requirements and timelines set by the FDA.

7.5 Expedited Reporting to Pfizer

Within 24 business hours of first awareness of the event the Overall PI will report to Pfizer by facsimile or email any Serious Adverse Event ("SAE," as defined below) for which reporting is required under this provision (as described below). Such SAEs are to be reported for study subjects or individuals otherwise exposed to the Pfizer Product as described below. The Overall PI should report SAEs as soon as they are determined to meet the definition, even if complete information is not yet available.

Principal Investigators will report SAEs using the provided Investigator-Initiated Research Serious Adverse Event Form (IIR SAE). The Reportable Event Fax Cover Sheet provided by Pfizer must also be included with each SAE submitted.

7.5.1 SAE Definition.
An SAE is any adverse event, without regard to causality, that is life-threatening (ie, causes an immediate risk of death) or that results in any of the following outcomes: death; in-patient hospitalization or prolongation of existing hospitalization; persistent or significant disability or incapacity (ie, substantial disruption of the ability to conduct normal life functions); or a congenital anomaly or birth defect. Any other medical event that, in the medical judgment of the Principal Investigator, may jeopardize the subject or may require medical or surgical intervention to prevent one of the outcomes listed above is also considered an SAE. A planned medical or surgical procedure is not, in itself, an SAE.

7.5.2 Exposure During Pregnancy, Exposure During Lactation, Occupational Exposure
Even though there may not be an associated SAE, exposure to the Palbociclib during pregnancy, exposure to the Palbociclib during lactation, and occupational exposure to the Palbociclib are reportable to Pfizer.

7.5.3 Hy's Law Cases
Cases of potential drug-induced liver injury as assessed by laboratory test values ("Hy's Law Cases") are also reportable to Pfizer. If a participant develops abnormal values in aspartate transaminase (AST) or alanine transaminase or both, concurrent with abnormal elevations in total bilirubin and no other known cause of liver injury, that event would be classified as a Hy's Law Case.

7.5.4 Exclusions from SAE Reporting Requirements
Specifically excluded from the reporting requirements is any SAE judged by the Overall Investigator to represent progression of the malignancy under study, unless it results in death within the SAE Reporting Period.

7.5.5 SAE Reporting Period

The SAEs that are subject to this reporting provision are those that occur from after the first dose of the Palbociclib through 30 calendar days after the last administration of the Palbociclib, or longer if so specified in the Protocol. In addition, if a Principal Investigator becomes aware of an SAE occurring any time after the administration of the last dose of the Palbociclib, the Principal Investigator should report that SAE to Pfizer if the Principal Investigator suspects a causal relationship between the Palbociclib and the SAE.

7.6 Expedited Reporting to Hospital Risk Management

Participating investigators will report to their local Risk Management office any participant safety reports or sentinel events that require reporting according to institutional policy.

7.7 Routine Adverse Event Reporting

All Grade 2 or greater Adverse Events **must** be reported in routine study data submissions to the Overall PI on the toxicity case report forms. **AEs reported through expedited processes (e.g., reported to the IRB, FDA, etc.) must also be reported in routine study data submissions.**

8. PHARMACEUTICAL INFORMATION

A list of the adverse events and potential risks associated with the investigational or other agents administered in this study can be found in Section 7.1.

8.1 Palbociclib

8.1.1 Description

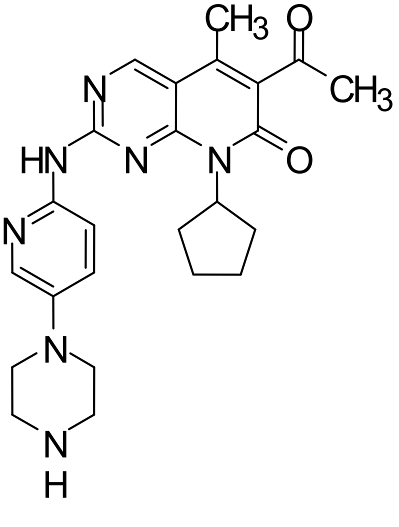

Chemical name: 6-acetyl-8-cyclopentyl-5-methyl-2-(5-(piperazin-1-yl)pyridin-2-

ylamino)pyrido[2,3-d]pyrimidin-7(8H)-one
Chemical formula: $C_{24}H_{29}N_7O_2$
Molecular weight: 447.53
Half life: ~27 hours
Plasma protein binding of palbociclib: ~85%
Plasma protein binding of PF-05089326 (the lactam of palbociclib, one of the main metabolites present in plasma): 95%

Palbociclib is metabolized to multiple metabolites in a qualitatively similar manner in rat, dog and human liver microsomes. In vitro, Palbociclib is primarily metabolized by CYP3A4 enzymes. Information on potential drug interactions can be found in Appendix B.

8.1.2 Form

Palbociclib will be supplied by Pfizer from commerical supply as capsules containing 75 mg, 100 mg, or 125 mg equivalents of palbociclib free base. Pfizer will supply the oral drug formulation to sites in High Density Polyethylene (HDPE) bottles containing 75 mg, 100 mg, or 125 mg capsules. The capsules can be differentiated by their size and color (see below).

Table 8-1 – Palbociclib capsule characteristics

Dosage	Capsule color	Capsule size
**75 mg**	Sunset Yellow/Sunset Yellow	2
**100 mg**	Caramel/Sunset Yellow	1
**125 mg**	Caramel/Caramel	0

8.1.3 Storage and Stability

Storage conditions stated in the Single Reference Safety Document (i.e., Investigator's Brochure (IB), United States Package Insert (USPI), Summary of Product Characteristics (SPC), or Local Product Document (LPD)) will be superseded by the label storage.

Palbociclib capsules should be stored at controlled room temperature (15-30°C, 59-86°F) in their original container.

Investigators and site staff are reminded to check temperatures daily (i.e. manually or by using alarm systems to alert of any excursions) and ensure that thermometers are working correctly as required for proper storage of investigational products. These include thermometers for both the room storage and refrigerator storage. Any temperature excursions must be reported to Pfizer. The investigational products must be stored as indicated. Deviations from the storage requirements, including any actions taken, must be documented and reported to Pfizer.

Once a deviation is identified, the investigational products must be quarantined and not used until Pfizer provides documentation of permission to use the investigational product.

Medication should be kept in a secured locked area at the study site in accordance with applicable regulatory requirements. Returned medication should be stored separately from

medication that needs to be dispensed.

8.1.4 Compatibility

No compatibility issues are known for co-administration of palbociclib and everolimus or exemestane.

8.1.5 Handling

Qualified personnel, familiar with procedures that minimize undue exposure to themselves and the environment, should undertake the preparation, handling, and safe disposal of the chemotherapeutic agent in a self-contained and protective environment.

8.1.6 Availability

Palbociclib will be supplied free-of-charge from Pfizer.

8.1.7 Administration

Palbociclib will be provided in non-participant-specific bottles containing 75 mg, 100 mg or 125 mg capsules.

The medical record number should be recorded on the bottle label in the spaces provided by site personnel at the time of assignment to participant. Site personnel must ensure that participants clearly understand the directions for self-medication. Participants should be given a sufficient supply to last until their next study visit. Unused drug and/or empty bottles should be returned to the site at the next study visit. Unused returned medication MUST NOT be re-dispensed to participants.

Palbociclib is an agent that must be handled and administered with care. Participants should be instructed to keep their medication in the bottles provided and not transfer it to any other container. Due to possible unknown hazards associated with topical and environmental exposure to experimental agents, capsules must not be opened and/or emptied into any vehicle for oral ingestion; capsules must be swallowed intact.

Only a single capsule strength will be dispensed to the participant at each dispensing visit. In the event of dose modification, request should be made of the participant to return all previously dispensed medication to the clinic and new capsules will be dispensed.

8.1.8 Ordering

Palbociclib will be provided to the Institutional pharmacy by Pfizer.

8.1.9 Accountability

To ensure adequate records, palbociclib capsules will be accounted for as instructed by Pfizer. Participants are requested to return previously dispensed containers as well as their completed drug diary to the clinic at each visit for accountability purposes even if they will not be issued with new medication at that visit.

The investigator, or a responsible party designated by the investigator, is responsible for maintaining a careful record of the inventory and disposition of the agent (investigational or free of charge) using the NCI Drug Accountability Record or another comparable drug accountability form. (See the CTEP website at http://ctep.cancer.gov/protocolDevelopment for the "Policy and Guidelines for Accountability and Storage of Investigational Agents" or to obtain a copy of the drug accountability form.)

8.1.10 Destruction and Return

Any undispensed/unopened investigational drug will be returned to the sponsor or destroyed onsite per institutional guidelines following the completion of research drug dosing by the last subject on the protocol. A member of the research team should notify the research pharmacy at all sites at which the trial is being conducted when the last subject on the protocol at that site has completed research drug dosing.

Returned supply by a participant will be destroyed per institutional guidelines following reconciliation by the pharmacy staff.

8.2 Everolimus

8.2.1 Description

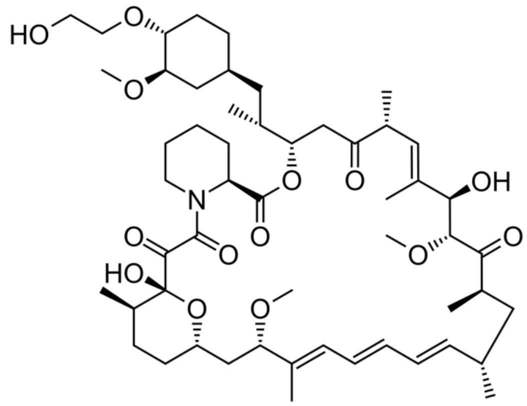

Chemical name: dihydroxy-12-[(2R)-1-[(1S,3R,4R)-4-(2-hydroxyethoxy)-3-methoxycyclohexyl]propan-2-yl]-19,30-dimethoxy-15,17,21,23,29,35-hexamethyl-11,36-dioxa-4-azatricyclo[30.3.1.0 hexatriaconta-16,24,26,28-tetraene-2,3,10,14,20-pentone

Chemical formula: $C_{53}H_{83}NO_{14}$
Molecular weight: 958.224
Half life: ~30 hours
Plasma protein binding of everolimus: ~74%

Everolimus is extensively metabolized in the liver via CYP3A4; forms 6 weak metabolites. Information on potential drug interactions can be found in Appendix B.

8.2.2 Form

Everolimus is commercially available as 2.5mg, 5mg, and 10 mg tablets.

8.2.3 Storage and Stability

Everolimus should be stored according to the package insert.

8.2.4 Compatibility

Everolimus was shown to increase exposure of exemestane. No compatibility issues are known for co-administration of everolimus and palbociclib.

8.2.5 Handling

Qualified personnel, familiar with procedures that minimize undue exposure to themselves and the environment, should undertake the preparation, handling, and safe disposal of the chemotherapeutic agent in a self-contained and protective environment.

8.2.6 Availability

Everolimus is commercially available as Afinitor®.

8.2.7 Administration

Everolimus will be dispensed in non-participant-specific bottles containing 2.5 mg, 5 mg or 10 mg tablets.

8.2.8 Ordering

Everolimus is commercially available.

8.2.9 Accountability

The investigator or designee will comply with all institutional commercial drug SOPs.

8.2.10 Destruction and Return

N/A

8.3 Exemestane

8.3.1 Description

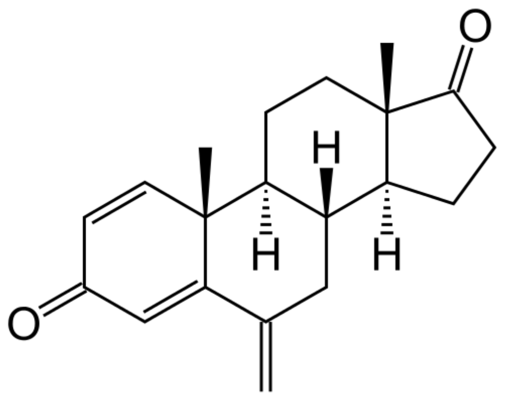

Chemical name: 6-Methylideneandrosta-1,4-diene-3,17-dione
Chemical formula: $C_{20}H_{24}O_2$
Molecular weight: 296.403
Half life: ~24 hours
Plasma protein binding of palbociclib: ~90%

In vitro, exemestane is primarily metabolized by CYP3A4 enzymes. Information on potential drug interactions can be found in Appendix B.

8.3.2 Form

Exemestane is commercially available as 25 mg of exemestane.

8.3.3 Storage and Stability

Storage conditions stated in the Single Reference Safety Document (i.e., United States Package Insert (USPI), Summary of Product Characteristics (SPC), or Local Product Document (LPD)) will be superseded by the label storage.

Investigators and site staff are reminded to check temperatures daily (i.e. manually or by using alarm systems to alert of any excursions) and ensure that thermometers are working correctly as required for proper storage of investigational products. These include thermometers for both the room storage and refrigerator storage. Any temperature excursions must be reported to the overall study PI. The investigational products must be stored as indicated. Deviations from the

storage requirements, including any actions taken, must be documented and reported to the overall study PI.

Once a deviation is identified, the investigational products must be quarantined and not used until the overall study PI provides documentation of permission to use the investigational product.

Medication should be kept in a secured locked area at the study site in accordance with applicable regulatory requirements. Returned medication should be stored separately from medication that needs to be dispensed.

8.3.4 Compatibility

Everolimus (that is given in the same regimen) was shown to increase exposure of exemestane. No other compatibility issues are known for co-administration with everolimus and palbociclib.

8.3.5 Handling

Only qualified personnel, familiar with institutional procedures should handle the agent.

8.3.6 Availability

Exemestane will be from commerical supply.

8.3.7 Administration

Exemestane will be provided in non-participant-specific bottles containing 25 mg tablets.

The participant number should be recorded on the bottle label in the spaces provided by site personnel at the time of assignment to participant. Site personnel must ensure that participants clearly understand the directions for self-medication. Participants should be given a sufficient supply to last until their next study visit. Unused drug and/or empty bottles should be returned to the site at the next study visit. Unused returned medication MUST NOT be re-dispensed to participants.

Exemestane is an agent that must be handled and administered with care. Participants should be instructed to keep their medication in the bottles provided and not transfer it to any other container. Due to possible unknown hazards associated with topical and environmental exposure to experimental agents, tablets must not be broken and must be swallowed intact.

8.3.8 Ordering

Exemestane will be provided to the Institutional Pharmacy by Pfizer.

8.3.9 Accountability

To ensure adequate records, exemestane tablets will be accounted for as instructed by Pfizer. Participants are requested to return previously dispensed containers as well as their completed drug diary to the clinic at each visit for accountability purposes even if they will not be issued with new medication at that visit.

The investigator, or a responsible party designated by the investigator, must maintain a careful record of the inventory and disposition of the agent (investigational or free of charge) using the NCI Drug Accountability Record or another comparable drug accountability form. (See the CTEP website at http://ctep.cancer.gov/protocolDevelopment for the "Policy and Guidelines for Accountability and Storage of Investigational Agents" or to obtain a copy of the drug accountability form.)

8.3.10 Destruction and Return

At the end of the trial, exemestane will be destroyed and documented as per institutional policies.

9. BIOMARKER, CORRELATIVE, AND SPECIAL STUDIES

9.1 cfDNA Collection

For a number of reasons that include discomfort to the participant, logistical challenges, and lack of clinical indication, serial biopsies in individual participants in the metastatic setting are infrequently performed. It has therefore been challenging to study the molecular evolution of tumors over time and through progression on serial treatment regimens. However, emerging technology now permits whole exome sequencing from cell-free DNA (cfDNA) isolated from fresh blood. We aim to examine the utility of this approach for detecting molecular changes in individual participants' tumor cell populations over time by performing serial cfDNA.

9.1.1 Objectives

- To collect blood specimens to characterize tumors, and bank for future studies including molecular evaluations of disease characteristics, genetic variability, and immunological function.

9.1.2 Specimen Collection and Handling

For cfDNA analyses, two 10 mL blood samples will be drawn into EDTA-containing "purple top" test tubes or Streck Tubes. Blood will be collected at baseline, restaging visits, and at time of progression/off study. Within two hours the cfDNA will be processed and banked in the DF/HCC Clinical Trials Core laboratory for future research purposes.

Given the time sensitive nature of processing these samples, please email the DF/HCC Clinical Trials Core laboratory (dfcibreastbank@partners.org) with the patient name, study ID, date of collection, approximate time of collection and study time point the day prior to collection.

It is anticipated that future studies will include evaluation of genetic, immunologic, hormonal, and/or other issues related to breast cancer. We will inform participants in the informed consent document that we will use their specimens for research related to breast cancer and other cancers.

9.2 Exome Analysis

9.2.1 Objectives

- To complete pharmacogenomic analyses related to drug response or adverse drug reactions
- To collect and perform germline exome sequencing as a control for the paired tumor biopsies.

9.2.2 Specimen Collection

One 10mL purple top tube and two CellSave tubes should be collected at Cycle 1 Day 1.

9.2.3 Specimen Handling/Shipping

The purple top tube will be brought/sent on the day of collection to:

The Wagle Lab
Nikhil Wagle, MD
Dana-Farber Cancer Institute
450 Brookline Avenue
Boston, MA 02215

The CellSave tubes will be sent ambient, same day to:

Broad Institute of MIT and Harvard
Greg Gydush
75 Ames St. Lab 75A-4045
Cambridge, MA 02142

9.2.4 Sites

Dana-Farber
Broad Institute of MIT and Harvard

9.3 Pharmacokinetic Studies

Sampling will be performed in all patients enrolled to the Phase I portion to define the whole blood concentration-time profile of everolimus over a single 24 hour dosing interval for the initial dose given on day 1and for the dose given on day 15 of cycle 1. Additional samples will be collected at each of the same times relative to the dose given on day 15 to define the plasma concentration-time profiles for exemestane and palbociclib. The sampling schedule has been devised to accommodate treatment on an outpatient basis.

Blood samples will be obtained from a peripheral arm vein shortly before dosing on days 1 and 15 and then at 0.5, 1.0, 2.0, 4.0, 6.0, 8.0, and 24.0 h after dosing. The 24.0 samples must be collected before the next daily doses of the three drugs are taken on days 2 and 16. It is very important that the patient is aware that the morning doses of all three drugs on days 2 and 16 must not be taken before arriving at the clinic and the 24.0 h pharmacokinetic samples have been collected.

The Vacutainer tubes used to collect the pharmacokinetic samples are as follows:

- Everolimus: 2 mL green top sodium heparin Vacutainer tube (Becton Dickinson cat. # 367671)
- Exemestane: 6 mL green top sodium heparin Vacutainer tube (Becton Dickinson cat. # 367878)
- Palbociclib: 4 mL lavender top K2EDTA Vacutainer tube (Becton Dickinson cat. # 367861)

Blood samples will be collected in all three types of collection tubes prior to giving the initial dose on day 1. Blood samples will only be collected in the 2 mL green top Vacutainer tubes for the 7 time points after dosing on day 1 and day 2 (24 hours after the day 1 dose, before the day 2 dose). Blood samples will be collected in all three tubes shortly before dosing on day 15 and at each of the 7 time points after dosing on day 15 and day 16 (24 hours after the day 15 dose, before the day 16 dose). The total volume of blood collected for pharmacokinetic studies will be 122 mL over a time interval of 16 days.

A Laboratory Manual will be prepared by the DF/HCC Cancer Pharmacology Core with detailed instructions for sample collection and handling, processing the samples for storage, storage conditions, packaging, and shipment. In addition, the Core will prepare a Pharmacokinetic Dosing and Sample Collection Time Form and a template for printing labels for the blood collections tubes and sample transport vials. These materials will be made available to the study sites in advance enrolling patients into the study.

9.4 Tissue Collection: The molecular underpinnings of CDK 4/6 inhibitors response and resistance

A baseline research biopsy prior to study therapy initiation is required in the phase IIa part of this study, and optional in the phase Ib part. Additionally, a second research biopsy will be requested at the time of disease progression in the case that participants have not started on a new treatment regimen. We plan to use this paired information to perform a number of studies characterizing disease response and resistance to therapy. Background information on the rationale for these investigations is discussed in Section 2.6. Paired evaluations at the DNA, RNA and protein levels, respectively by whole exome sequencing, RNA Seq and immunohistochemistry will be performed. Moreover, we will bank specimens for possible future studies.

9.4.1 Objectives

To characterize the landscape of genomic changes to provide insight about determinants of

response and resistance to CDK 4/6 inhibitors. To seek for IHC correlates of genomic changes. To bank samples for future studies, including molecular evaluations of disease characteristics, genetic variability and immunological function.

9.4.2 Hypothesis

We hypothesize that molecular analysis of tumor samples at baseline and at disease progression will allow for the identification of molecular determinants of response and resistance to CDK 4/6 inhibitors, namely concerning p16-cyclin D/CDK-Rb-E2F and p53 pathways aberrations. We further hypothesize that some of these findings can be assessed using IHC techniques.

9.4.3 Collection, Handling and Shipping of Specimens

Tissue specimens will be collected from metastatic lesions using standard institutional procedures. Core biopsies are required and fine needle aspirates cannot be used. All samples will be anonymized by assigning a unique sample ID number prior to use.

Following research biopsy of a specimen, 1 tissue core will be placed in formalin and sent to the pathology department at BWH for H&E staining, IHC for receptor testing as well as specific Rb pathway investigation under Dr. Scott J. Rodig supervision. The other cores will be frozen in OCT and sent to the lab of Dr Nikhil Wagle at the Dana-Farber Cancer Institute/Broad Institute of Harvard and MIT for whole exome/transcriptome sequencing. The tissue not used will be stored for future research.

The amount of tissue collected will follow the guidelines listed below:

- Breast: A goal of 3-6 core biopsy specimens will be obtained using standard institutional guidelines for a diagnostic core biopsy of a breast mass.
- Skin/chest wall: A goal of 1-2 5-mm punch biopsies will be obtained.
- Lymph node: A goal of 3-6 core biopsy specimens will be obtained using an 18-gauge needle.
- Liver: A goal of 3-6 core biopsy specimens will be obtained using an 18-gauge needle.
- Lung: Because of the risk of pneumothorax associated with core needle biopsies of lung nodules, no core biopsies of lung nodules will be performed on this protocol; unless if they are clinically indicated.
- Bone: Because the yield of malignant tissue from bone biopsies tends to be relatively low, if a participant has another accessible site of disease (i.e. skin, lymph node, liver), that site should be biopsied preferentially. If bone is the only biopsy-accessible site, then a goal of 3-6 core biopsy specimens will be obtained using an 11-13 gauge needle.
- Pleural Fluid: A goal of 500 cc of pleural fluid will be obtained with a standard thoracentesis procedure, with or without image guidance, according to the clinical judgment of the treating physician and clinician performing the procedure. Less than the goal amount is acceptable, and should be based upon the clinical judgment of the Investigator and the clinician performing the procedure. If more than the goal amount of fluid is obtained, then the entire specimen (with the exception of what is needed for clinical purposes, if applicable) will be stored in the tissue bank.

- Ascites fluid: A goal of 500 cc of ascites fluid will be obtained with a standard paracentesis procedure, with or without image guidance, according to the clinical judgment of the treating physician and clinician performing the procedure. Less than the goal amount is acceptable, and should be based upon the clinical judgment of the Investigator and the clinician performing the procedure. If more than the goal amount of fluid is obtained, then the entire specimen (with the exception of what is needed for clinical purposes, if applicable) will be stored in the tissue bank.

Of note, the above indications are guidelines for the amount of tissue to be obtained, and are not meant to replace clinical judgment at the time the procedure is performed. Less than the goal quantity of tissue is accepted for each type of biopsy, and will be left to the clinical judgment of the physician performing the procedure. If ascites or pleural fluid is to be used as the investigational biopsy specimen, consideration should be given to confirming the malignant nature of the ascites or pleural fluid prior to study entry.

If a participant is undergoing resection of a lesion for clinical reasons (i.e. wedge resection of a new lung lesion for confirmation of diagnosis or re-testing of hormone receptor or HER2 status; or, resection of a chest wall lesion; or, resection of a lymph node), then the participant may opt to have a portion of that tissue (roughly equivalent to the goal amount of tissue listed in the guidelines above, i.e. the equivalent of two 5-mm punch biopsies of the skin, or 3-6 18-gauge core biopsies) stored for research at the time of the procedure, in which case, the participant would not be required to undergo a separate research biopsy for entry into this protocol.

If a participant has more than one site of disease, only one site needs to be biopsied, and the site is left to the discretion of the participant and her treating physician. Participants who undergo an attempted research biopsy procedure for the purpose of this protocol, and in whom inadequate tissue is obtained, are not required to undergo a repeat biopsy in order to continue on protocol.

For archival tissue collection, tissue blocks of primary and/or metastatic tumor samples should be sent to Dana-Farber Cancer Institute. If blocks cannot be submitted, 20 unstained slides are acceptable.

9.4.4 Site(s) Performing Correlative Study

Wagle Lab
Nikhil Wagle, MD, PhD
Dana-Farber Cancer Institute
450 Brookline Avenue
Boston, MA 02215

9.4.5 Tissue banking

All leftover tissue will be banked in the Dana-Farber Cancer Institute Breast Cancer Tissue Bank under the supervision of Dr. Deborah A. Dillon as per standard lab protocol, such that it can be used for additional or future analyses as needed.

9.4.6 Additional Analysis

The above-mentioned analyses may be altered based on novel developments in the field of genomic analysis at the time of correlative science. Additional markers or alternative technologies (based on scientific developments and/or novel technologies) may also be used, to explore potential prognostic or predictive candidate markers/panels or markers related to treatment benefit and/or safety, to improve diagnostic tests, or to understand breast cancer biology.

10. STUDY CALENDAR

Table 10-1 lists all of the assessments and indicates with an "X", the visits when they are performed. Assessments must be performed prior to administration of any study agent. Study assessments and agents should be administered within ± 3 days of the protocol-specified date, unless otherwise noted. Laboratory assessments that were completed within 3 days prior to Day 1 of each cycle (e.g. cycle 1, day 1) do not need to be repeated. For efficacy assessments there is a ±7 days window. Baseline evaluations are to be conducted within 21 days (14 days for labs) week prior to start of protocol therapy. Screening CT/MRI scans and pre-treatment tumor biopsy must be completed within 28 days before the first dose of study treatment. In the event that the participant's condition is deteriorating, laboratory evaluations should be repeated within 48 hours prior to initiation of the next cycle of therapy.

Table 10-1 – Study calendar

		Screening	Treatment phase									End of therapy	30 Day F/U	Disease Progression F/U
			Cycle 1						Cycle 2		Subsequent cycles			
Day of cycle		-28 to -1	1	2	8	15	16	21	1	15	1	≤14 days after decision to discontinue		Phase II only
Informed consent/Demographics		X												
Inclusion /exclusion criteria		X												
Medical and surgical history/prior cancer history		X												
Concurrent meds		X	X		X	X		X	X	X	X	X		
Physical exam[a]		X	X		X	X		X	X	X	X	X		
Height		X												
Vital Signs[b]		X	X		X	X		X	X	X	X	X		
Performance status		X	X						X		X	X		
Single, 12-lead ECG		X			X[c]				X					
Blood collection	Hematology	X	X		X	X		X	X	X	X	X		
	Chemistry[d]	X	X		X	X		X	X	X	X	X		
	Fasting glucose	X												
	PK Sampling Phase Ib[e]		X	X		X	X							
	cfDNA Collection		X								X[i]	X[i]	X[i]	X[i]
	Exome Sequencing		X											
Adverse event evaluation			X		X	X		X	X	X	X	X		
CT/MRI tumor assessment		X									X[f]	X		X[g]
Bone scan		X												
Research tumor biopsy		X[j]											X[h, j]	X[h, j]

	Screening	Treatment phase									End of therapy	30 Day F/U	Disease Progression F/U
		Cycle 1						Cycle 2		Subsequent cycles			
Day of cycle	-28 to -1	1	2	8	15	16	21	1	15		≤14 days after decision to discontinue	1	Phase II only
Archival tumor sample (Phase IIa part only)	X												
Antineoplastic therapies since discontinuation													X
Phone calls for disease progression F/U evaluation													X

a. A complete physical examination that evaluates all major organ systems will be performed at Screening/baseline. Subsequent physical exams may be limited and should be focused on sites of disease to explore clinical signs and symptoms.
b. Vital signs to include: diastolic and systolic blood pressure, heart rate, temperature, and weight
c. ECG on C1D8 is only required for participants enrolled in the phase Ib part of the study.
d. Chemistries to include: Albumin, alkaline phosphatase, total bilirubin, bicarbonate, BUN, calcium, chloride, creatinine, glucose, potassium, total protein, SGOT [AST], SGPT [ALT], and sodium.
e. PK samples will be collected on days 1 and 15 immediately before dosing and at 30min, 1hr, 2hr, 4hr, 6hr, and 8hr after dosing with a +/- 5 minute window allowed for each time point. The 24hr PK samples will be collected within a +/- 60minute window on days 2 and 16 prior to dosing.
f. Radiologic measurements should be performed every 8 weeks. Tumor evaluations will be performed within days prior to Day 1 of cycles 3, 5 and 7. Subsequent evaluations will be performed within 7 days prior to day 1 of every 4th cycle, or sooner if there is clinical evidence of disease progression. Radiologic documentation must be provided for participants removed from study for progressive disease.
g. Tumor evaluations and follow-up will be performed every 8 weeks (if the participants is within 24 weeks of initiation of study treatment) or every 12 weeks (if the participant is greater than 24 weeks from initiation of study treatment); time counting since last tumor evaluation and performed within ±2 weeks.
h. Performed at within 30 days of disease progression and prior to participant initiating another anti-tumor treatment.
i. Sample collected for cfDNA at baseline, each restaging visit, end of study, and time of disease progression.
j. Baseline research biopsy is required for participants enrolled to the Phase II portion and optional for patients enrolled to the Phase I portion. A research biopsy is optional at time of progression for Phase Iband Phase II participants.

11. MEASUREMENT OF EFFECT

Although response is not the primary endpoint of the phase Ib part of this trial, participants with measurable disease will be assessed by standard criteria. As for the phase IIa part, the measure of efficacy will be CBR, defined as the proportion of participants with complete response, partial response or stable disease for more than 6 months.

11.1 Antitumor Effect – Solid Tumors

For the purposes of this study, participants should be re-evaluated for response every 8 weeks. After the end of therapy visit, tumor evaluations will be performed every 8 weeks (if the participants are within 24 weeks of initiation of study treatment) or every 12 weeks (if the participant is greater than 24 weeks from initiation of study treatment). In addition to a baseline scan, confirmatory scans should also be obtained 4 weeks following initial documentation of objective response.

Response and progression will be evaluated in this study using the new international criteria proposed by the Response Evaluation Criteria in Solid Tumors (RECIST) guideline (version 1.1) [*Eur J Ca* 45:228-247, 2009]. Changes in the largest diameter (unidimensional measurement) of the tumor lesions and the shortest diameter in the case of malignant lymph nodes are used in the RECIST criteria.

11.1.1 Definitions

Evaluable for Target Disease response. Only those participants who have measurable disease present at baseline, have received at least one cycle of therapy, and have had their disease re-evaluated will be considered evaluable for target disease response. These participants will have their response classified according to the definitions stated below. (Note: Participants who exhibit objective disease progression prior to the end of cycle 1 will also be considered evaluable.)

Evaluable Non-Target Disease Response. Participants who have lesions present at baseline that are evaluable but do not meet the definitions of measurable disease, have received at least one cycle of therapy, and have had their disease re-evaluated will be considered evaluable for non-target disease. The response assessment is based on the presence, absence, or unequivocal progression of the lesions.

11.1.2 Disease Parameters

Measurable disease. Measurable lesions are defined as those that can be accurately measured in at least one dimension (longest diameter to be recorded) as ≥ 20 mm by chest x-ray or ≥10 mm with CT scan, MRI, or calipers by clinical exam. All tumor measurements must be recorded in millimeters (or decimal fractions of centimeters).

Note: Tumor lesions that are situated in a previously irradiated area might or might not be considered measurable. Overall, such lesions will not be considered measurable in this study.

Malignant lymph nodes. To be considered pathologically enlarged and measurable, a lymph node must be ≥15 mm in short axis when assessed by CT scan (CT scan slice thickness recommended to be no greater than 5 mm). At baseline and in follow-up, only the short axis will be measured and followed.

Non-measurable disease. All other lesions (or sites of disease), including small lesions (longest diameter <10 mm or pathological lymph nodes with ≥10 to <15 mm short axis), are considered non-measurable disease. Bone lesions, leptomeningeal disease, ascites, pleural/pericardial effusions, lymphangitis cutis/pulmonitis, inflammatory breast disease, abdominal masses (not followed by CT or MRI), and cystic lesions are all considered non-measurable.

Note: Cystic lesions that meet the criteria for radiographically defined simple cysts should not be considered as malignant lesions (neither measurable nor non-measurable) since they are, by definition, simple cysts.

'Cystic lesions' thought to represent cystic metastases can be considered as measurable lesions, if they meet the definition of measurability described above. However, if non-cystic lesions are present in the same participant, these are preferred for selection as target lesions.

Target lesions. All measurable lesions up to a maximum of 2 lesions per organ and 5 lesions in total, representative of all involved organs, should be identified as **target lesions** and recorded and measured at baseline. Target lesions should be selected on the basis of their size (lesions with the longest diameter), be representative of all involved organs, but in addition should be those that lend themselves to reproducible repeated measurements. It may be the case that, on occasion, the largest lesion does not lend itself to reproducible measurement in which circumstance the next largest lesion which can be measured reproducibly should be selected. A sum of the diameters (longest for non-nodal lesions, short axis for nodal lesions) for all target lesions will be calculated and reported as the baseline sum diameters. If lymph nodes are to be included in the sum, then only the short axis is added into the sum. The baseline sum diameters will be used as reference to further characterize any objective tumor regression in the measurable dimension of the disease.

Non-target lesions. All other lesions (or sites of disease) including any measurable lesions over and above the 5 target lesions should be identified as **non-target lesions** and should also be recorded at baseline. Measurements of these lesions are not required, but the presence, absence, or in rare cases unequivocal progression of each should be noted throughout follow up.

11.1.3 Methods for Evaluation of Disease

All measurements should be taken and recorded in metric notation using a ruler, calipers, or a digital measurement tool. All baseline evaluations should be performed as closely as

possible to the beginning of treatment and never more than 4 weeks before the beginning of the treatment.

The same method of assessment and the same technique should be used to characterize each identified and reported lesion at baseline and during follow-up. Imaging-based evaluation is preferred to evaluation by clinical examination unless the lesion(s) being followed cannot be imaged but are assessable by clinical exam.

Clinical lesions. Clinical lesions will only be considered measurable when they are superficial (*e.g.*, skin nodules and palpable lymph nodes) and ≥10 mm in diameter as assessed using calipers (*e.g.*, skin nodules). In the case of skin lesions, documentation by color photography, including a ruler to estimate the size of the lesion, is recommended.

Chest x-ray. Lesions on chest x-ray are acceptable as measurable lesions when they are clearly defined and surrounded by aerated lung; however, CT is preferable.

Conventional CT and MRI. This guideline has defined measurability of lesions on CT scan based on the assumption that CT thickness is 5mm or less. If CT scans have slice thickness greater than 5 mm, the minimum size of a measurable lesion should be twice the slice thickness. MRI is also acceptable in certain situations (*e.g.* for body scans).

Use of MRI remains a complex issue. MRI has excellent contrast, spatial, and temporal resolution; however, there are many image acquisition variables involved in MRI, which greatly impact image quality, lesion conspicuity, and measurement. Furthermore, the availability of MRI is variable globally. As with CT, if an MRI is performed, the technical specifications of the scanning sequences used should be optimized for the evaluation of the type and site of disease. Furthermore, as with CT, the modality used at follow-up should be the same as was used at baseline and the lesions should be measured/assessed on the same pulse sequence. It is beyond the scope of the RECIST guidelines to prescribe specific MRI pulse sequence parameters for all scanners, body parts, and diseases. Ideally, the same type of scanner should be used and the image acquisition protocol should be followed as closely as possible to prior scans. Body scans should be performed with breath-hold scanning techniques, if possible.

FDG-PET. While FDG-PET response assessments need additional study, it is sometimes reasonable to incorporate the use of FDG-PET scanning to complement CT scanning in assessment of progression (particularly possible 'new' disease). New lesions on the basis of FDG-PET imaging can be identified according to the following algorithm:

(a) Negative FDG-PET at baseline, with a positive FDG-PET at follow-up is a sign of PD based on a new lesion.

(b) No FDG-PET at baseline and a positive FDG-PET at follow-up: If the positive FDG-PET at follow-up corresponds to a new site of disease confirmed by CT, this is PD. If the positive FDG-PET at follow-up is not confirmed as a new site of disease on CT, additional follow-up CT scans are needed to determine if there is truly progression occurring at that site (if so, the date of PD will be the date of the initial abnormal FDG-PET scan). If the positive FDG-PET at follow-up corresponds to a

pre-existing site of disease on CT that is not progressing on the basis of the anatomic images, this is not PD.

(c) FDG-PET may be used to upgrade a response to a CR in a manner similar to a biopsy in cases where a residual radiographic abnormality is thought to represent fibrosis or scarring. The use of FDG-PET in this circumstance should be prospectively described in the protocol and supported by disease-specific medical literature for the indication. However, it must be acknowledged that both approaches may lead to false positive CR due to limitations of FDG-PET and biopsy resolution/sensitivity.

Note: A 'positive' FDG-PET scan lesion means one which is FDG avid with an uptake greater than twice that of the surrounding tissue on the attenuation corrected image.

PET-CT. At present, the low dose or attenuation correction CT portion of a combined PET-CT is not always of optimal diagnostic CT quality for use with RECIST measurements. However, if the site can document that the CT performed as part of a PET-CT is of identical diagnostic quality to a diagnostic CT (with IV and oral contrast), then the CT portion of the PET-CT can be used for RECIST measurements and can be used interchangeably with conventional CT in accurately measuring cancer lesions over time. Note, however, that the PET portion of the CT introduces additional data which may bias an investigator if it is not routinely or serially performed.

MIBG (meta-iodobenzylguanidine).The following is recommended, to assure high quality images are obtained.

Participant preparation: Iodides, usually SSKI (saturated solution of potassium iodide), are administered to reduce thyroidal accumulation of free radioiodine, preferably beginning the day prior to injection and continuing for 3 additional days (4 days total). For infants and children, one drop t.i.d. is sufficient, for adolescents 2 drops t.i.d., and for adults 3 drops t.i.d. Participants and/or parents are asked about exposure to potential interfering agents. If none is noted, an indwelling intravenous line is established. The dose of MIBG is administered by slow intravenous injection over 90 seconds.

Images from the head to the distal lower extremities should be obtained.

I-123MIBG scintigraphy is performed to obtain both planar and tomographic images.

Planar: Anterior and posterior views from the top of the head to the proximal lower extremities are obtained for 10 minutes at 24 hours and occasionally at 48 hours following injection of 10 mCi/1.7 square meters of body surface area (~150 μCi/kg, maximum 10 mCi). Anterior views of the distal lower extremities are adequate. A large field of view dual head gamma camera with low energy collimators is preferred.

SPECT: Most participants receiving I-123 MIBG also undergo SPECT at 24 hours,

using a single or multi-headed camera with a low energy collimator. The camera is rotated through 360 degrees, 120 projections at 25 seconds per stop. Data are reconstructed using filtered back projections with a Butterworth filter and a cut off frequency of 0.2-0.5. SPECT/CT may be performed at institutions with this capacity.

Ultrasound. Ultrasound is not useful in assessment of lesion size and should not be used as a method of measurement. Ultrasound examinations cannot be reproduced in their entirety for independent review at a later data and, because they are operator dependent, it cannot be guaranteed that the same technique and measurements will be taken from one assessment to the next. If new lesions are identified by ultrasound in the course of the study, confirmation by CT or MRI is advised. If there is concern about radiation exposure from CT, MRI may be used instead of CT in selected instances.

Endoscopy, Laparoscopy. The utilization of these techniques for objective tumor evaluation is not advised. However, such techniques may be useful to confirm complete pathological response when biopsies are obtained or to determine relapse in trials where recurrence following complete response (CR) or surgical resection is an endpoint.

Tumor markers. Tumor markers alone cannot be used to assess response. If markers are initially above the upper normal limit, they must normalize for a participant to be considered in complete clinical response. Specific guidelines for both CA-125 response (in recurrent ovarian cancer) and PSA response (in recurrent prostate cancer) have been published [*JNCI* 96:487-488, 2004; *J Clin Oncol* 17, 3461-3467, 1999; *J Clin Oncol* 26:1148-1159, 2008]. In addition, the Gynecologic Cancer Intergroup has developed CA-125 progression criteria which are to be integrated with objective tumor assessment for use in first-line trials in ovarian cancer [*JNCI* 92:1534-1535, 2000].

Cytology, Histology. These techniques can be used to differentiate between partial responses (PR) and complete responses (CR) in rare cases (*e.g.*, residual lesions in tumor types, such as germ cell tumors, where known residual benign tumors can remain).

The cytological confirmation of the neoplastic origin of any effusion that appears or worsens during treatment when the measurable tumor has met criteria for response or stable disease is mandatory to differentiate between response or stable disease (an effusion may be a side effect of the treatment) and progressive disease.

11.1.3.1 Evaluation of Target Lesions

Complete Response (CR): Disappearance of all target lesions. Any pathological lymph nodes (whether target or non-target) must have reduction in short axis to <10 mm.

Partial Response (PR): At least a 30% decrease in the sum of the diameters of target lesions, taking as reference the baseline sum diameters.

Progressive Disease (PD): At least a 20% increase in the sum of the diameters of

target lesions, taking as reference the smallest sum on study (this includes the baseline sum if that is the smallest on study). In addition to the relative increase of 20%, the sum must also demonstrate an absolute increase of at least 5 mm. (Note: the appearance of one or more new lesions is also considered progressions).

Stable Disease (SD): Neither sufficient shrinkage to qualify for PR nor sufficient increase to qualify for PD, taking as reference the smallest sum diameters while on study.

11.1.3.2 Evaluation of Non-Target Lesions

Complete Response (CR): Disappearance of all non-target lesions and normalization of tumor marker level. All lymph nodes must be non-pathological in size (<10 mm short axis).

Note: If tumor markers are initially above the upper normal limit, they must normalize for a participant to be considered in complete clinical response.

Non-CR/Non-PD: Persistence of one or more non-target lesion(s) and/or maintenance of tumor marker level above the normal limits.

Progressive Disease (PD): Appearance of one or more new lesions and/or *unequivocal progression* of existing non-target lesions. *Unequivocal progression* should not normally trump target lesion status. It must be representative of overall disease status change, not a single lesion increase.

Although a clear progression of "non-target" lesions only is exceptional, the opinion of the treating physician should prevail in such circumstances, and the progression status should be confirmed at a later time by the review panel (or Principal Investigator).

11.1.3.3 Evaluation of New Lesions

The finding of a new lesion should be unequivocal (i.e. not due to difference in scanning technique, imaging modality, or findings thought to represent something other than tumor (for example, some 'new' bone lesions may be simply healing or flare of pre-existing lesions). However, a lesion identified on a follow-up scan in an anatomical location that was not scanned at baseline is considered new and will indicate PD. If a new lesion is equivocal (because of small size etc.), follow-up evaluation will clarify if it truly represents new disease and if PD is confirmed, progression should be declared using the date of the initial scan on which the lesion was discovered.

11.1.3.4 Evaluation of Best Overall Response

The best overall response is the best response recorded from the start of the treatment until disease progression/recurrence (taking as reference for progressive disease the

smallest measurements recorded since the treatment started). The participant's best response assignment will depend on the achievement of both measurement and confirmation criteria.

For Participants with Measurable Disease (*i.e.*, Target Disease)

<table>
<tr><th>Target Lesions</th><th>Non-Target Lesions</th><th>New Lesions</th><th>Overall Response</th><th>Best Overall Response when Confirmation is Required*</th></tr>
<tr><td>CR</td><td>CR</td><td>No</td><td>CR</td><td>≥4 wks Confirmation**</td></tr>
<tr><td>CR</td><td>Non-CR/Non-PD</td><td>No</td><td>PR</td><td rowspan="3">≥4 wks Confirmation**</td></tr>
<tr><td>CR</td><td>Not evaluated</td><td>No</td><td>PR</td></tr>
<tr><td>PR</td><td>Non-CR/Non-PD/not evaluated</td><td>No</td><td>PR</td></tr>
<tr><td>SD</td><td>Non-CR/Non-PD/not evaluated</td><td>No</td><td>SD</td><td>Documented at least once ≥4 wks from baseline**</td></tr>
<tr><td>PD</td><td>Any</td><td>Yes or No</td><td>PD</td><td rowspan="3">no prior SD, PR or CR</td></tr>
<tr><td>Any</td><td>PD***</td><td>Yes or No</td><td>PD</td></tr>
<tr><td>Any</td><td>Any</td><td>Yes</td><td>PD</td></tr>
<tr><td colspan="5">* See RECIST 1.1 manuscript for further details on what is evidence of a new lesion.
** Only for non-randomized trials with response as primary endpoint.
*** In exceptional circumstances, unequivocal progression in non-target lesions may be accepted as disease progression.

<u>Note</u>: Participants with a global deterioration of health status requiring discontinuation of treatment without objective evidence of disease progression at that time should be reported as "symptomatic deterioration." Every effort should be made to document the objective progression even after discontinuation of treatment.</td></tr>
</table>

For Participants with Non-Measurable Disease (*i.e.*, Non-Target Disease)

Non-Target Lesions	New Lesions	Overall Response
CR	No	CR
Non-CR/non-PD	No	Non-CR/non-PD*
Not all evaluated	No	not evaluated
Unequivocal PD	Yes or No	PD
Any	Yes	PD

* 'Non-CR/non-PD' is preferred over 'stable disease' for non-target disease since SD is increasingly used as an endpoint for assessment of efficacy in some trials so to assign this category when no lesions can be measured is not advised

11.1.4 Duration of Response

Duration of overall response: The duration of overall response is measured from the time measurement criteria are met for CR or PR (whichever is first recorded) until the first date that recurrent or progressive disease is objectively documented (taking as reference for progressive disease the smallest measurements recorded since the treatment started, or death due to any cause. Participants without events reported are censored at the last disease evaluation).

Duration of overall complete response: The duration of overall CR is measured from the time measurement criteria are first met for CR until the first date that progressive disease is objectively documented, or death due to any cause. Participants without events reported are censored at the last disease evaluation.

Duration of stable disease: Stable disease is measured from the start of the treatment until the criteria for progression are met, taking as reference the smallest measurements recorded since the treatment started, including the baseline measurements.

11.1.5 Clinical benefit rate

Clinical Benefit Rate: Clinical Benefit Rate (CBR) is defined as the proportion of participants achieving complete response, partial response or stable disease for more than 6 months taking as reference the smallest measurements recorded since the treatment started, including the baseline measurements.

11.1.6 Progression-Free Survival

Progression-Free Survival: Progression-Free Survival (PFS) is defined as the time from randomization (or registration) to the earlier of progression or death due to any cause. Participants alive without disease progression are censored at date of last disease evaluation.

Time to Progression: Time to Progression (TTP) is defined as the time from randomization (or registration) to progression, or censored at date of last disease evaluation for those without progression reported.

11.1.7 Response Review

All responses will be reviewed by an expert, independent of the study, at the study's completion with simultaneous review of the participants' files and radiological images.

12. DATA REPORTING / REGULATORY REQUIREMENTS

Adverse event lists, guidelines, and instructions for AE reporting can be found in Section 7.0 (Adverse Events: List and Reporting Requirements).

12.1 Data Reporting

12.1.1 Method

The ODQ will collect, manage, and perform quality checks on the data for this study.

12.1.2 Responsibility for Data Submission

Investigative sites within DF/HCC or DF/PCC are responsible for submitting data and/or data forms to the ODQ according to the schedule set by the ODQ.

12.2 Data Safety Monitoring

The DF/HCC Data and Safety Monitoring Committee (DSMC) will review and monitor toxicity and accrual data from this study. The committee is composed of clinical specialists with experience in oncology and who have no direct relationship with the study. Information that raises any questions about participant safety will be addressed with the Overall PI and study team.

The DSMC will review each protocol up to four times a year or more often if required to review toxicity and accrual data. Information to be provided to the committee may include: up-to-date participant accrual; current dose level information; DLT information; all grade 2 or higher unexpected adverse events that have been reported; summary of all deaths occurring with 30 days of intervention for Phase I or II protocols; for gene therapy protocols, summary of all deaths while being treated and during active follow-up; any response information; audit results, and a summary provided by the study team. Other information (e.g. scans, laboratory values) will be provided upon request.

12.3 Multicenter Guidelines

N/A.

12.4 Collaborative Agreements Language

N/A.

13. STATISTICAL CONSIDERATIONS

13.1 Study Design/Endpoints

This is a single center, single arm, open-label, phase Ib/ IIa study. We will include participants with ER+/Her2− advanced breast cancer that progressed on a CDK 4/6 inhibitor regimen and a NSAI for the treatment with palbociclib plus everolimus plus exemestane. Participants will be accrued in a single-stage fashion. For the phase Ib part of the study, after registration, participants will proceed in independent groups for dose-escalation using a classic 3+3 rule. Dose levels are detailed in Table 5-2 and escalation will proceed as detailed in Table 5-3.

Table 13-1 – Operating characteristics of the 3+3 dose escalation design under different true rates of dose limiting toxicity (DLT).

	**Dose Escalation using 3+3 rule**						
True DLT rate	0.1	0.2	0.3	0.4	0.5	0.6	0.7
Probability	91%	71%	49%	31%	17%	8%	3%

For the phase IIa of the study, participants will be enrolled using the MTD or RP2D obtained from phase Ib part. Two research biopsies are required over the course of study (collection is not mandatory during phase Ib): one at pre-treatment baseline (biopsy 1) and a second at disease progression (biopsy 2).

Toxicity will be graded and reported as per NCI CTCAE version 4.03. All participants who receive any amount of the study drug will be evaluable for toxicity. DLTs are defined in Section 5.2 and listed in Table 5-4. MTD is defined in section 5.3 and either the MTD or the maximum dose of palbociclib 125 mg plus everolimus 10 mg plus exemestane 25 mg will be the doses used for further testing in phase IIa part of the study.

Primary endpoints:

- Phase Ib part: To describe safety, tolerability and MTD/RP2D of the combination of palbociclib plus everolimus plus exemestane;
- Phase IIa part: To estimate efficacy, as defined by CBR, of the combination of palbociclib plus everolimus plus exemestane.

Secondary endpoints:

Secondary clinical endpoints

- Phase IIa part: To estimate the activity of the combination of palbociclib plus everolimus plus exemestane, as defined by overall response rate (ORR), disease control rate (DCR), duration of response (DOR) and progression free-survival (PFS) according to investigator assessment;
- To describe the PK profile of everolimus and palbociclib in the triple combination and evaluate the DDI potential (effect of palbociclib on the PK profile of everolimus).

Correlative science endpoints

- To investigate biomarkers of sensitivity and resistance to CDK 4/6 inhibitors via whole exome analysis and RNA Seq of paired tumor biopsies;

13.2 Sample Size, Accrual Rate and Study Duration

Phase IIa study part

A total of 32 participants will be accrued within one year (Table 13-1). Up to an additional 6 months of follow-up will be required on the last participant accrued to observe the participant's response after the 6th cycle of protocol therapy, for a total study duration of 1.5 years.

In this study participants will receive the triplet combination of palbociclib plus everolimus and exemestane after progression to a CDK4/6 inhibitor regimen and to an NSAI. Despite the fact that palbociclib was not available at the time of the approval of the combination of everolimus plus exemestane (BOLERO-2[19,90]), everolimus plus exemestane is still considered a standard regimen in this setting. Using this combination, the observed CBR (as per central assessment) was of 49.9% in the everolimus + exemestane arm and of 22.2% in the exemestane plus placebo arm.[90] Provided that in the population of the current trial participants were further exposed to a CDK4/6 inhibitor regimen, we estimate that the true CBR in this population with the treatment with everolimus plus exemestane is of 40%. Therefore, we will use 40% as the estimated true CBR. We consider that a CBR equal or superior to 65% with the combination of palbociclib plus everolimus and exemestane would be worthy of further study.

Table 13-2 – Sample size using the exact binomial method as a function of one sided alpha and power.

One-sided alpha	Power	Sample size	min # of positive to reject null	Exact alpha
0.1	80%	19	11	0.09
0.1	90%	29	16	0.07
0.05	80%	28	16	0.05
0.05	90%	34	19	0.04

Note: Exact binomial method was used with a one-sided alpha of 0.1 and type II error of 0.1 (90% power). The study needs to enroll 29 evaluable participants to test CBR 65% against a null hypothesis of 40% (exact alpha=0.07). In this setting, if at least 16 out of 29 participants have clinical benefit, the regimen is worthy further investigation. Admitting a 10% drop out rate, we will recruit 32 participants.

Mandatory biopsies for correlative studies

We anticipate that 90% of pairs of tumor specimens will be evaluable for resistance mutation. Mutation seen in pre- and post-treatment samples will be summarized using contingency tables. With 26 pairs of pre- and post-treatment samples, there are 41% and 74% probabilities of observing at least one resistance mutation if the true frequency of resistance mutation is 2% and 5%, respectively; and 90% power to detect 25% resistance mutation (e.g. mutation prevalence increases from 5% to 30% or from 25% to 50% relative to pre-treatment samples), assuming that 1% of participants unexpectedly show mutations in only the pre-treatment biopsy (Table 13-2; McNemar's test, one-sided α=0.1).

Table 13-3 – Sample size using McNemar's method as a function of one sided alpha and power.

Total participants enrolled	Paired samples evaluable for mutation	Prob. of obs. at least one resistance mutation if freq. is 2%	Prob. of obs. at least one resistance mutation if freq. is 5%	Min. detectable change in prevalence of mutation post-

				treatment
19	17	29%	58%	36%
29	26	41%	74%	25%
28	25	40%	72%	26%
34	30	45%	79%	22%

Note: McNemar's test with one sided alpha of 0.1 and type II error of 0.1 (90% power) was used to calculate the minimal detectable change in prevalence of mutation post-treatment, assuming that 1% of participants unexpectedly show mutations in only the pre-treatment biopsy.

13.3 Stratification Factors

No stratifications or interim monitoring determinations will be performed.

13.4 Interim Monitoring Plan

N/A.

13.5 Analysis of Primary Endpoints

Phase Ib
The determination of the MTD under the dose de-escalation scheme is defined in Table 5-3. Treatment-related toxicities will be summarized by maximum grade and by term using CTCAE v4.0 and reported with 90% binomial exact confidence intervals.

Phase IIa
CBR is defined in Section 11. The primary endpoint will be analyzed in all the participants eligible for the phase IIa part of the study. Descriptive statistics will be used to summarize CBR (proportion). The 95% CI will also be calculated for the proportion of participants reaching the definition of CBR. We consider that a CBR equal or superior to 65% with the combination of palbociclib plus everolimus and exemestane would be worthy of further study.

13.6 Analysis of Secondary Endpoints

Overall response rate (ORR), disease control rate (DCR) and duration of response (DOR) are defined in Section 11. Response will be assessed among participants eligible for the phase IIa part of the study who received at least one dose of the study drugs at the MTD/RP2D and have measurable disease at screening. Radiographic response will be assessed using RECIST 1.1 criteria as defined in section Section 11. The ORR, DCR and DOR will be reported with 95% confidence intervals.

Progression free-survival (PFS) is defined in Section 11. PFS will be described using the method of Kaplan-Meier, and it will be presented with a 95% confidence interval. Participants alive without disease progression are censored at the date of last disease evaluation.

Correlative objectives will be presented as descriptive statistics, using summary statistics (such as mean, SD, median, IQR and range). Correlation to responses will be reported as odds-ratios with 95% confidence intervals. Specifically, we plan to measure Rb and p53 status by immunohistochemistry in baseline biopsies, and correlate baseline Rb and p53 status with response (by RECIST) as an exploratory analysis.

13.7 Reporting and Exclusions

13.7.1 Evaluation of Toxicity

All participants will be evaluable for toxicity from the time of their first treatment. Participants who never start protocol therapy will be considered inevaluable.

13.7.2 Evaluation of the Primary Efficacy Endpoint

Analyses will be performed as intent-to-treat and restrict to those participants eligible for the phase IIa part of the study. Participants who never start protocol therapy will be considered inevaluable.

14. PUBLICATION PLAN

The results should be made public within 24 months of reaching the end of the study. The end of the study is the time point at which the last data items are to be reported, or after the outcome data are sufficiently mature for analysis, as defined in the section on Sample Size, Accrual Rate and Study Duration. If a report is planned to be published in a peer-reviewed journal, then that initial release may be an abstract that meets the requirements of the International Committee of Medical Journal Editors. A full report of the outcomes should be made public no later than three (3) years after the end of the study. The Primary Investigator will be the final arbiter of the manuscript content.

REFERENCES

1. Jemal A, Bray F, Center MM, Ferlay J, Ward E, Forman D. Global cancer statistics. *CA Cancer J Clin*. 2011;61(2):69-90. doi:10.3322/caac.20107.

2. Siegel R, Ma J, Zou Z, Jemal A. Cancer statistics, 2014. *CA Cancer J Clin*. 2014;64(1):9-29. doi:10.3322/caac.21208.

3. Clarke M, Collins R, Darby S, Davies C, Evans V, Godwin J, Gray R, McGale P, Peto R, Wang Y.Clarke M, Collins R, Darby S, Davies C, Evans V, Godwin J, Gray R, McGale P, Peto R WY. Effects of chemotherapy and hormonal therapy for early breast cancer on recurrence and 15-year survival: an overview of the randomised trials. *Lancet*. 2005;365(9472):1687-717. doi:10.1016/S0140-6736(05)66544-0.

4. Seah DSE, Luis IV, Macrae E, et al. Use and duration of chemotherapy in participants with metastatic breast cancer according to tumor subtype and line of therapy. *J Natl Compr Canc Netw*. 2014;12(1):71-80. Available at: http://www.ncbi.nlm.nih.gov/pubmed/24453294.

5. Anderson WF, Rosenberg PS, Prat A, Perou CM, Sherman ME. How many etiological subtypes of breast cancer: two, three, four, or more? *J Natl Cancer Inst*. 2014;106(8):1-11. doi:10.1093/jnci/dju165.

6. Clarke CA, Keegan THM, Yang J, et al. Age-Specific Incidence of Breast Cancer Subtypes: Understanding the Black–White Crossover. *J Natl Cancer Inst*. 2012;104(14):1094-1101. doi:10.1093/jnci/djs264.

7. Davies C, Godwin J, Gray R, et al. Relevance of breast cancer hormone receptors and other factors to the efficacy of adjuvant tamoxifen: participant-level meta-analysis of randomised trials. *Lancet*. 2011;378(9793):771-84. doi:10.1016/S0140-6736(11)60993-8.

8. Burstein HJ, Temin S, Anderson H, et al. Adjuvant Endocrine Therapy for Women With Hormone Receptor-Positive Breast Cancer: American Society of Clinical Oncology Clinical Practice Guideline Focused Update. *J Clin Oncol*. 2014;32(21):2255-2269. doi:10.1200/JCO.2013.54.2258.

9. National Comprehensive Cancer Network. NCCN Clinical Practice Guidelines in Oncology (NCCN Guidelines): breast cancer. Version 2.2015. March 11, 2015. 2015. Available at: http://www.nccn.org/professionals/physician_gls/pdf/breast.pdf. Accessed June 9, 2015.

10. Coates AS, Winer EP, Goldhirsch A, et al. Tailoring therapies—improving the management of early breast cancer: St Gallen International Expert Consensus on the Primary Therapy of Early Breast Cancer 2015. *Ann Oncol*. 2015;26(8):1533-1546. doi:10.1093/annonc/mdv221.

11. Early Breast Cancer Trialists' Collaborative Group (EBCTCG). Aromatase inhibitors versus tamoxifen in early breast cancer: participant-level meta-analysis of the randomised trials. *Lancet*. 2015. doi:10.1016/S0140-6736(15)61074-1.

12. Johnston SRD. Targeting downstream effectors of epidermal growth factor receptor/HER2 in breast cancer with either farnesyltransferase inhibitors or mTOR antagonists. In: *International Journal of Gynecological Cancer*.Vol 16.; 2006:543-548. doi:10.1111/j.1525-1438.2006.00692.x.

13. Schiff R, Massarweh SA, Shou J, et al. Cross-Talk between Estrogen Receptor and Growth Factor Pathways As A Molecular Target for Overcoming Endocrine Resistance. In: *Clinical Cancer Research*.Vol 10.; 2004. doi:10.1158/1078-0432.CCR-031212.

14. Miller WR. Aromatase inhibitors: prediction of response and nature of resistance. *Expert Opin Pharmacother*. 2010;11(11):1873-1887. doi:10.1517/14656566.2010.487863.

15. Yamnik RL, Digilova A, Davis DC, Brodt ZN, Murphy CJ, Holz MK. S6 kinase 1 regulates estrogen receptor alpha in control of breast cancer cell proliferation. *J Biol Chem*. 2009;284(10):6361-6369. doi:10.1074/jbc.M807532200.

16. Yamnik RL, Holz MK. mTOR/S6K1 and MAPK/RSK signaling pathways coordinately regulate estrogen receptor alpha serine 167 phosphorylation. *FEBS Lett*. 2010;584(1):124-128. doi:10.1016/j.febslet.2009.11.041.

17. Efeyan A, Sabatini DM. MTOR and cancer: Many loops in one pathway. *Curr Opin Cell Biol*. 2010;22(2):169-176. doi:10.1016/j.ceb.2009.10.007.

18. Boulay A, Rudloff J, Ye J, et al. Dual inhibition of mTOR and estrogen receptor signaling in vitro induces cell death in models of breast cancer. *Clin Cancer Res*. 2005;11(14):5319-5328. doi:10.1158/1078-0432.CCR-04-2402.

19. Baselga J, Campone M, Piccart M, et al. Everolimus in Postmenopausal Hormone-Receptor–Positive Advanced Breast Cancer. *N Engl J Med*. 2012;366(6):520-529. doi:10.1056/NEJMoa1109653.

20. O'Shaughnessy JA, Kaufmann M, Siedentopf F, et al. Capecitabine Monotherapy: Review of Studies in First-Line HER-2-Negative Metastatic Breast Cancer. *Oncologist*. 2012;17(4):476-484. doi:10.1634/theoncologist.2011-0281.

21. Jager E, Al-Batran S, Saupe S, et al. A randomized phase III study evaluating pegylated liposomal doxorubicin (PLD) versus capecitabine (CAP) as first-line therapy for metastatic breast cancer (MBC): Results of the PELICAN study. *J Clin Oncol*. 2010;28(15).

22. Kaufmann M, Maass N, Costa SD, et al. First-line therapy with moderate dose capecitabine in metastatic breast cancer is safe and active: Results of the MONICA trial. *Eur J Cancer*. 2010;46(18):3184-3191. doi:10.1016/j.ejca.2010.07.009.

23. Stockler M, Sourjina T, Grimison P, et al. A randomized trial of capecitabine (C) given intermittently (IC) rather than continuously (CC) compared to classical CMF as first-line chemotherapy for advanced breast cancer (ABC). *ASCO Meet Abstr*. 2007;25(18_suppl):1031.

24. Robert NJ, Dieras V, Glaspy J, et al. RIBBON-1: Randomized, double-blind, placebo-controlled, phase III trial of chemotherapy with or without bevacizumab (B) for first-line treatment of HER2-negative locally recurrent or metastatic breast cancer (MBC). *J Clin Oncol*. 2009;27(15):1005.

25. Choi YJ, Anders L. Signaling through cyclin D-dependent kinases. *Oncogene*. 2014;33(15):1890-903. doi:10.1038/onc.2013.137.

26. Malumbres M, Barbacid M. Cell cycle, CDKs and cancer: a changing paradigm. *Nat Rev Cancer*. 2009;9(3):153-66. doi:10.1038/nrc2602.

27. Burkhart DL, Sage J. Cellular mechanisms of tumour suppression by the retinoblastoma gene. *Nat Rev Cancer*. 2008;8(9):671-82. doi:10.1038/nrc2399.

28. Fry DW, Harvey PJ, Keller PR, et al. Specific inhibition of cyclin-dependent kinase 4/6 by PD 0332991 and associated antitumor activity in human tumor xenografts. *Mol Cancer Ther*. 2004;3(11):1427-38. Available at: http://www.ncbi.nlm.nih.gov/pubmed/15542782. Accessed May 14, 2015.

29. Finn RS, Dering J, Conklin D, et al. PD 0332991, a selective cyclin D kinase 4/6 inhibitor, preferentially inhibits proliferation of luminal estrogen receptor-positive human breast cancer cell lines in vitro. *Breast Cancer Res*. 2009;11(5):R77. doi:10.1186/bcr2419.

30. Finn R, Hurvitz S, Allison M, et al. Phase I Study of PD 0332991, a Novel, Oral, Cyclin-D Kinase (CDK) 4/6 Inhibitor in Combination with Letrozole, for First-Line Treatment of Metastatic Post-Menopausal, Estrogen Receptor-Positive (ER+), Human Epidermal Growth Factor Receptor 2 (HER2)-Negati. *Cancer Res*. 2014;69(24 Supplement):5069-5069. doi:10.1158/0008-5472.SABCS-09-5069.

31. Finn RS, Crown JP, Lang I, et al. The cyclin-dependent kinase 4/6 inhibitor palbociclib in combination with letrozole versus letrozole alone as first-line treatment of oestrogen receptor-positive, HER2-negative, advanced breast cancer (PALOMA-1/TRIO-18): a randomised phase 2 study. *Lancet Oncol*. 2014;16(1):25-35. doi:10.1016/S1470-2045(14)71159-3.

32. DeMichele A, Clark AS, Tan KS, et al. CDK 4/6 Inhibitor Palbociclib (PD0332991) in Rb+ Advanced Breast Cancer: Phase II Activity, Safety, and Predictive Biomarker

Assessment. *Clin Cancer Res*. 2015;21(5):995-1001. doi:10.1158/1078-0432.CCR-14-2258.

33. Beroukhim R, Mermel CH, Porter D, et al. The landscape of somatic copy-number alteration across human cancers. *Nature*. 2010;463(7283):899-905. doi:10.1038/nature08822.

34. Sherr CJ, Roberts JM. CDK inhibitors: positive and negative regulators of G1-phase progression. *Genes Dev*. 1999;13(12):1501-1512. doi:10.1101/gad.13.12.1501.

35. Van den Heuvel S, Harlow E. Distinct roles for cyclin-dependent kinases in cell cycle control. *Science (80-)*. 1993;262(5142):2050-2054. doi:10.1126/science.8266103.

36. Weinberg RA. The retinoblastoma protein and cell cycle control. *Cell*. 1995;81(3):323-330. doi:10.1016/0092-8674(95)90385-2.

37. Harper JW, Adami GR, Wei N, Keyomarsi K, Elledge SJ. The p21 Cdk-interacting protein Cip1 is a potent inhibitor of G1 cyclin-dependent kinases. *Cell*. 1993;75(4):805-816. doi:10.1016/0092-8674(93)90499-G.

38. Koff A, Ohtsuki M, Polyak K, Roberts JM, Massague J. Negative regulation of G1 in mammalian cells: inhibition of cyclin E- dependent kinase by TGF-beta. *Science (80-)*. 1993;260(5107):536-539.

39. Polyak K, Kato JY, Solomon MJ, et al. p27Kip1, a cyclin-Cdk inhibitor, links transforming growth factor-beta and contact inhibition to cell cycle arrest. *Genes Dev*. 1994;8(1):9-22. doi:10.1101/gad.8.1.9.

40. Sherr CJ. D-type cyclins. *Trends Biochem Sci*. 1995;20(5):187-190. doi:10.1016/S0968-0004(00)89005-2.

41. Toyoshima H, Hunter T. p27, A novel inhibitor of G1 cyclin-Cdk protein kinase activity, is related to p21. *Cell*. 1994;78(1):67-74. doi:10.1016/0092-8674(94)90573-8.

42. Lukas J, Parry D, Aagaard L, et al. Retinoblastoma-protein-dependent cell-cycle inhibition by the tumour suppressor p16. *Nature*. 1995;375(6531):503-506. doi:10.1038/375503a0.

43. Medema RH, Herrera RE, Lam F, Weinberg RA. Growth suppression by p16ink4 requires functional retinoblastoma protein. *Proc Natl Acad Sci U S A*. 1995;92(14):6289-6293. doi:10.1073/pnas.92.14.6289.

44. Butt AJ, McNeil CM, Musgrove EA, Sutherland RL. Downstream targets of growth factor and oestrogen signalling and endocrine resistance: The potential roles of c-Myc, cyclin D1 and cyclin E. In: *Endocrine-Related Cancer*.Vol 12.; 2005. doi:10.1677/erc.1.00993.

45. Altucci L, Addeo R, Cicatiello L, et al. Estrogen induces early and timed activation of cyclin-dependent kinases 4, 5, and 6 and increases cyclin messenger ribonucleic acid expression in rat uterus. *Endocrinology*. 1997;138(3):978-984. doi:10.1210/en.138.3.978.

46. Geum D, Sun W, Paik SK, Lee CC, Kim K. Estrogen-induced cyclin D1 and D3 gene expressions during mouse uterine cell proliferation in vivo: Differential induction mechanism of cyclin D1 and D3. *Mol Reprod Dev*. 1997;46(4):450-458. doi:10.1002/(SICI)1098-2795(199704)46:4<450::AID-MRD2>3.0.CO;2-N.

47. Watts CK, Brady A, Sarcevic B, deFazio A, Musgrove EA, Sutherland RL. Antiestrogen inhibition of cell cycle progression in breast cancer cells in associated with inhibition of cyclin-dependent kinase activity and decreased retinoblastoma protein phosphorylation. *Mol Endocrinol*. 1995;9(12):1804-1813. doi:10.1210/me.9.12.1804.

48. Thangavel C, Dean JL, Ertel A, et al. Therapeutically activating RB: Reestablishing cell cycle control in endocrine therapy-resistant breast cancer. *Endocr Relat Cancer*. 2011;18(3):333-345. doi:10.1530/ERC-10-0262.

49. Yu Q, Sicinska E, Geng Y, et al. Requirement for CDK4 kinase function in breast cancer. *Cancer Cell*. 2006;9(1):23-32. doi:10.1016/j.ccr.2005.12.012.

50. Konecny GE, Winterhoff B, Kolarova T, et al. Expression of p16 and retinoblastoma determines response to CDK4/6 inhibition in ovarian cancer. *Clin Cancer Res*. 2011;17(6):1591-1602. doi:10.1158/1078-0432.CCR-10-2307.

51. Cen L, Carlson BL, Schroeder MA, et al. P16-Cdk4-Rb axis controls sensitivity to a cyclin-dependent kinase inhibitor PD0332991 in glioblastoma xenograft cells. *Neuro Oncol*. 2012;14(7):870-881. doi:10.1093/neuonc/nos114.

52. Angela DeMichele, Amy Sanders Clark, Daniel Heitjan, Sophia Randolph, Maryann Gallagher, Priti Lal, Michael D Feldman, Paul J. Zhang, Allison Schnader, Kelly Zafman, Susan M. Domchek, Keerthi Gogineni, Stephen Michael Keefe, Kevin R. Fox PJO. A phase II trial of an oral CDK 4/6 inhibitor, PD0332991, in advanced breast cancer. *J Clin Oncol*. 2013;31(suppl; abstr 519):Suppl.

53. Finn R, Crown J, Boer K, et al. P1-17-05: Preliminary Results of a Randomized Phase 2 Study of PD 0332991, a Cyclin-Dependent Kinase (CDK) 4/6 Inhibitor, in Combination with Letrozole for First-Line Treatment of Participants (pts) with Post-Menopausal, ER+, HER2-Negative (HER2-) Advanced Br. *Cancer Res*. 2011;71(24 Supplement):P1-17-05-P1-17-05. doi:10.1158/0008-5472.SABCS11-P1-17-05.

54. Yu Y, Sato JD. MAP kinases, phosphatidylinositol 3-kinase, and p70 S6 kinase mediate the mitogenic response of human endothelial cells to vascular endothelial growth factor. *J Cell Physiol*. 1999;178(2):235-246. doi:10.1002/(SICI)1097-4652(199902)178:2<235::AID-JCP13>3.0.CO;2-S.

55. Lane HA, Wood JM, McSheehy PMJ, et al. mTOR inhibitor RAD001 (Everolimus) has antiangiogenic/vascular properties distinct from a VEGFR tyrosine kinase inhibitor. *Clin Cancer Res*. 2009;15(5):1612-1622. doi:10.1158/1078-0432.CCR-08-2057.

56. Guba M, von Breitenbuch P, Steinbauer M, et al. Rapamycin inhibits primary and metastatic tumor growth by antiangiogenesis: involvement of vascular endothelial growth factor. *Nat Med*. 2002;8(2):128-135. doi:10.1038/nm0202-128.

57. Tsutsumi N, Yonemitsu Y, Shikada Y, et al. Essential role of PDGFRalpha-p70S6K signaling in mesenchymal cells during therapeutic and tumor angiogenesis in vivo: role of PDGFRalpha during angiogenesis. *Circ Res*. 2004;94(9):1186-1194. doi:10.1161/01.RES.0000126925.66005.39.

58. Mabuchi S, Altomare DA, Connolly DC, et al. RAD001 (Everolimus) delays tumor onset and progression in a transgenic mouse model of ovarian cancer. *Cancer Res*. 2007;67(6):2408-2413. doi:10.1158/0008-5472.CAN-06-4490.

59. Marangoni E, Vincent-Salomon A, Auger N, et al. A new model of participant tumor-derived breast cancer xenografts for preclinical assays. *Clin Cancer Res*. 2007;13(13):3989-3998. doi:10.1158/1078-0432.CCR-07-0078.

60. Kuhn B, Jacobsen W, Christians U, Benet LZ, Kollman PA. Metabolism of sirolimus and its derivative everolimus by cytochrome P450 3A4: Insights from docking, molecular dynamics, and quantum chemical calculations. *J Med Chem*. 2001;44(12):2027-2034. doi:10.1021/jm010079y.

61. Crowe A, Lemaire M. In vitro and in situ absorption of SDZ-RAD using a human intestinal cell line (Caco-2) and a single pass perfusion model in rats: Comparison with rapamycin. *Pharm Res*. 1998;15(11):1666-1672. doi:10.1023/A:1011940108365.

62. Laplante A, Demeule M, Murphy GF, Béliveau R. Interaction of immunosuppressive agents rapamycin and its analogue SDZ-RAD with endothelial P-gp. *Transplant Proc*. 2002;34(8):3393-5. Available at: http://www.ncbi.nlm.nih.gov/pubmed/12493480. Accessed August 17, 2015.

63. Ellard SL, Clemons M, Gelmon KA, et al. Randomized phase II study comparing two schedules of everolimus in participants with recurrent/metastatic breast cancer: NCIC clinical trials group IND.163. *J Clin Oncol*. 2009;27(27):4536-4541. doi:10.1200/JCO.2008.21.3033.

64. Baselga J, Semiglazov V, Van Dam P, et al. Phase II randomized study of neoadjuvant everolimus plus letrozole compared with placebo plus letrozole in participants with estrogen receptor-positive breast cancer. *J Clin Oncol*. 2009;27(16):2630-2637. doi:10.1200/JCO.2008.18.8391.

65. Demers LM, Lipton A, Harvey HA, et al. *The Efficacy of CGS 20267 in Suppressing Estrogen Biosynthesis in Participants with Advanced Stage Breast Cancer.*; 1993. doi:10.1016/0960-0760(93)90283-3.

66. Plourde P V, Dyroff M, Dowsett M, Demers L, Yates R, Webster A. *ARIMIDEX: A New Oral, Once-a-Day Aromatase Inhibitor.*; 1995. doi:10.1016/0960-0760(95)00045-2.

67. Buzdar AU. Pharmacology and pharmacokinetics of the newer generation aromatase inhibitors. In: *Clinical Cancer Research*.Vol 9.; 2003.

68. Hutson PR, Love RR, Havighurst TC, Rogers E, Cleary JF. Effect of exemestane on tamoxifen pharmacokinetics in postmenopausal women treated for breast cancer. *Clin Cancer Res*. 2005;11(24):8722-8727. doi:10.1158/1078-0432.CCR-05-0915.

69. Cardoso F, Costa a., Norton L, et al. ESO-ESMO 2nd international consensus guidelines for advanced breast cancer (ABC2). *Ann Oncol*. 2014;25(10):1871-1888. doi:10.1093/annonc/mdu385.

70. Partridge AH, Rumble RB, Carey LA, et al. Chemotherapy and Targeted Therapy for Women With Human Epidermal Growth Factor Receptor 2-Negative (or unknown) Advanced Breast Cancer: American Society of Clinical Oncology Clinical Practice Guideline. *J Clin Oncol*. 2014;32(29):3307-3329. doi:10.1200/JCO.2014.56.7479.

71. Yuan TL, Cantley LC. PI3K pathway alterations in cancer: variations on a theme. *Oncogene*. 2008;27(41):5497-510. doi:10.1038/onc.2008.245.

72. Miller TW, Hennessy BT, González-Angulo AM, et al. Hyperactivation of phosphatidylinositol-3 kinase promotes escape from hormone dependence in estrogen receptor-positive human breast cancer. *J Clin Invest*. 2010;120(7):2406-13. doi:10.1172/JCI41680.

73. Herrera-Abreu MT, Asghar US, Elliot R, et al. Abstract 86O: PI3 kinase/mTOR inhibition increases sensitivity of ER positive breast cancers to CDK4/6 inhibition by blocking cell cycle re-entry driven by cyclinD1 and inducing apoptosis. *Ann Oncol*. 2015;26(suppl 3):iii29-iii29. doi:10.1093/annonc/mdv120.01.

74. Vora SR, Juric D, Kim N, et al. CDK 4/6 Inhibitors Sensitize PIK3CA Mutant Breast Cancer to PI3K Inhibitors. *Cancer Cell*. 2014;26(1):136-149. doi:10.1016/j.ccr.2014.05.020.

75. Musgrove EA, Caldon CE, Barraclough J, Stone A, Sutherland RL. Cyclin D as a therapeutic target in cancer. *Nat Rev Cancer*. 2011;11(8):558-572. doi:10.1038/nrc3090.

76. Turner NC, Ro J, André F, et al. Palbociclib in Hormone-Receptor-Positive Advanced Breast Cancer. *N Engl J Med*. 2015;373(3):209-19. doi:10.1056/NEJMoa1505270.

77. Gauthier ML, Berman HK, Miller C, et al. Abrogated response to cellular stress identifies DCIS associated with subsequent tumor events and defines basal-like breast tumors. *Cancer Cell*. 2007;12(5):479-91. doi:10.1016/j.ccr.2007.10.017.

78. Lukas J, Bartkova J, Rohde M, Strauss M, Bartek J. Cyclin D1 is dispensable for G1 control in retinoblastoma gene-deficient cells independently of cdk4 activity. *Mol Cell Biol*. 1995;15(5):2600-11. Available at: http://www.pubmedcentral.nih.gov/articlerender.fcgi?artid=230490&tool=pmcentrez&rendertype=abstract. Accessed May 17, 2015.

79. Dean JL, Thangavel C, McClendon AK, Reed CA, Knudsen ES. Therapeutic CDK4/6 inhibition in breast cancer: key mechanisms of response and failure. *Oncogene*. 2010;29(28):4018-32. doi:10.1038/onc.2010.154.

80. Ewen ME, Oliver CJ, Sluss HK, Miller SJ, Peeper DS. p53-dependent repression of CDK4 translation in TGF-beta-induced G1 cell-cycle arrest. *Genes Dev*. 1995;9(2):204-217. doi:10.1101/gad.9.2.204.

81. Miller SJ, Suthiphongchai T, Zambetti GP, Ewen ME. p53 Binds Selectively to the 5' Untranslated Region of cdk4, an RNA Element Necessary and Sufficient for Transforming Growth Factor beta - and p53-Mediated Translational Inhibition of cdk4. *Mol Cell Biol*. 2000;20(22):8420-8431. doi:10.1128/MCB.20.22.8420-8431.2000.

82. Konecny GE, Winterhoff B, Kolarova T, et al. Expression of p16 and retinoblastoma determines response to CDK4/6 inhibition in ovarian cancer. *Clin Cancer Res*. 2011;17(6):1591-602. doi:10.1158/1078-0432.CCR-10-2307.

83. Barton KL, Misuraca K, Cordero F, et al. PD-0332991, a CDK4/6 inhibitor, significantly prolongs survival in a genetically engineered mouse model of brainstem glioma. *PLoS One*. 2013;8(10):e77639. doi:10.1371/journal.pone.0077639.

84. Wagle N, Grabiner BC, Van Allen EM, et al. Response and Acquired Resistance to Everolimus in Anaplastic Thyroid Cancer. *N Engl J Med*. 2014;371(15):1426-1433. doi:10.1056/NEJMoa1403352.

85. Wagle N, Van Allen EM, Treacy DJ, et al. MAP kinase pathway alterations in BRAF-mutant melanoma participants with acquired resistance to combined RAF/MEK inhibition. *Cancer Discov*. 2014;4(1):61-8. doi:10.1158/2159-8290.CD-13-0631.

86. Van Allen EM, Wagle N, Sucker A, et al. The genetic landscape of clinical resistance to RAF inhibition in metastatic melanoma. *Cancer Discov*. 2014;4(1):94-109. doi:10.1158/2159-8290.cd-13-0617.

87. Wagle N, Emery C, Berger MF, et al. Dissecting therapeutic resistance to RAF inhibition in melanoma by tumor genomic profiling. *J Clin Oncol*. 2011;29(22):3085-3096. doi:10.1200/JCO.2010.33.2312.

88. Flaherty KT, Lorusso PM, Demichele A, et al. Phase I, dose-escalation trial of the oral cyclin-dependent kinase 4/6 inhibitor PD 0332991, administered using a 21-day schedule in participants with advanced cancer. *Clin Cancer Res*. 2012;18(2):568-76. doi:10.1158/1078-0432.CCR-11-0509.

89. Slamon DJ, Hurvitz S a., Applebaum S, et al. Phase I study of PD 0332991, cyclin-D kinase (CDK) 4/6 inhibitor in combination with letrozole for first-line treatment of participants with ER-positive, HER2-negative breast cancer. *J Clin Oncol 2815s, 2010 (suppl; abstr 3060)*. Available at: http://meetinglibrary.asco.org/content/50680-74. Accessed August 20, 2015.

90. Yardley DA, Noguchi S, Pritchard KI, et al. Everolimus plus exemestane in postmenopausal participants with HR(+) breast cancer: BOLERO-2 final progression-free survival analysis. *Adv Ther*. 2013;30(10):870-84. doi:10.1007/s12325-013-0060-1.

APPENDIX A PERFORMANCE STATUS CRITERIA

**ECOG Performance Status Scale**		**Karnofsky Performance Scale**	
Grade	Descriptions	Percent	Description
0	Normal activity. Fully active, able to carry on all pre-disease performance without restriction.	100	Normal, no complaints, no evidence of disease.
		90	Able to carry on normal activity; minor signs or symptoms of disease.
1	Symptoms, but ambulatory. Restricted in physically strenuous activity, but ambulatory and able to carry out work of a light or sedentary nature (*e.g.*, light housework, office work).	80	Normal activity with effort; some signs or symptoms of disease.
		70	Cares for self, unable to carry on normal activity or to do active work.
2	In bed <50% of the time. Ambulatory and capable of all self-care, but unable to carry out any work activities. Up and about more than 50% of waking hours.	60	Requires occasional assistance, but is able to care for most of his/her needs.
		50	Requires considerable assistance and frequent medical care.
3	In bed >50% of the time. Capable of only limited self-care, confined to bed or chair more than 50% of waking hours.	40	Disabled, requires special care and assistance.
		30	Severely disabled, hospitalization indicated. Death not imminent.
4	100% bedridden. Completely disabled. Cannot carry on any self-care. Totally confined to bed or chair.	20	Very sick, hospitalization indicated. Death not imminent.
		10	Moribund, fatal processes progressing rapidly.
5	Dead.	0	Dead.

APPENDIX B INFORMATION ON POSSIBLE DRUG INTERACTIONS

Information on Possible Interactions with Other Agents for Participants and Their Caregivers and Non-Study Healthcare Team

The participant ______________________________ is enrolled on a clinical trial using the experimental agents palbociclib (Ibrance®), everolimus (Afinitor®) and exemestane (Aromasin®). This form is addressed to the participant, but includes important information for others who care for this participant.

Both palbociclib and everolimus may interact with many drugs that are processed by your liver. Because of this, it is very important to tell your study doctors about all of your medicine before you start this study. It is also very important to tell them if you stop taking any regular medicine, or if you start taking a new medicine while you take part in this study. When you talk about your medicine with your study doctor, include medicine you buy without a prescription at the drug store (over-the-counter remedy), or herbal supplements such as St. John's wort.

Many health care prescribers can write prescriptions. You must also tell your other prescribers (doctors, physicians' assistants or nurse practitioners) that you are taking part in a clinical trial. **Bring this paper with you and keep the attached information card in your wallet**. These are the things that you and they need to know:

Palbociclib and everolimus interact with a certain specific enzyme in your liver.

- The enzyme in question is CYP3A, and both palbociclib and everolimus are broken down by this enzyme in order to be cleared from your system.
- Palbociclib and everolimus must be used carefully with other medicines that need these liver enzymes to be effective or to be cleared from your system.
- Other medicines may also affect the activity of the enzyme.
    - Substances that increase the enzyme's activity ("inducers") could reduce the effectiveness of the drug, while substances that decrease the enzyme's activity ("inhibitors") could result in high levels of the active drug, increasing the chance of harmful side effects.
    - Moreover, both palbociclib and everolimus are considered inhibitors of the enzyme, meaning that it can affect the levels of other drugs that are processed by that enzyme. This can lead to harmful side effects and/or reduce the effectiveness of those medications.
- You and healthcare providers who prescribe drugs for you must be careful about adding or removing any drug in this category.
- Before you start the study, your study doctor will work with your regular prescriber to switch any medicines that are considered "moderate or strong inducers/inhibitors or substrates of ***CYP3A***."
- Your prescribers should look at this web site http://medicine.iupui.edu/clinpharm/ddis/table.aspx or consult a medical reference to see if any medicine they want to prescribe is on a list of drugs to avoid.
- Please be very careful! Over-the-counter drugs have a brand name on the label—it's usually big and catches your eye. They also have a generic name—it's usually small and

located above or below the brand name, and printed in the ingredient list. Find the generic name and determine, with the pharmacist's help, whether there could be an adverse interaction.

- Be careful:
  - If you take acetaminophen regularly: You should not take more than 4 grams a day if you are an adult or 2.4 grams a day if you are older than 65 years of age. Read labels carefully, acetaminophen is an ingredient in many medicines for pain, flu, and cold.
  - If you drink grapefruit juice or eat grapefruit: Avoid these until the study is over.
  - If you take herbal medicine regularly: You should not take St. John's wort until the study is over.

Proton pump inhibitors(PPI) may be taken while on study, however it is recommended that the PPI is taken 12 hours from the time of palbociclib administration. If needed, alternative antacid therapies may be used including H2-receptor antagonists and locally acting antacids. H2-receptor antagonists should be administered with a staggered dosing regimen (twice daily). The dosing of palbociclib should occur at least 10 hours after H2-receptor antagonist evening dose and 2 hours before the H2-receptor antagonist morning dose. Local antacid should be given at least 2 hours before or after palbociclib administration.

Other medicines can be a problem with your study drugs.

- You should check with your doctor or pharmacist whenever you need to use an over-the-counter medicine or herbal supplement.

- Your regular prescriber should check a medical reference or call your study doctor before prescribing any new medicine for you. Your study doctor's name is

____

and he or she can be contacted at

____

INFORMATION ON POSSIBLE DRUG INTERACTIONS

You are enrolled on a clinical trial using the experimental agents **palbociclib**, **everolimus** and **exemestane. Palbociclib** and **everolimus** interact with drugs that are processed by your liver. Because of this, it is very important to:

- Tell your doctors if you stop taking regular medicine or if you start taking a new medicine.
- Tell all of your prescribers (doctor, physicians' assistant, nurse practitioner, and pharmacist) that you are taking part in a clinical trial.
- Check with your doctor or pharmacist whenever you need to use an over-the-counter medicine or herbal supplement.

Palbociclib and **everolimus** interact with a specific liver enzyme called **CYP3A**, and must be used very carefully with other medicines that interact with this enzyme.

- Before you start the study, your study doctor will work with your regular prescriber to switch any medicines that are considered "strong inducers/inhibitors or substrates of **CYP3A**."
- Before prescribing new medicines, your regular prescribers should go to http://medicine.iupui.edu/clinpharm/ddis/table.aspx for a list of drugs to avoid, or contact your study doctor.
- Your study doctor's name is ______________________________

  and can be contacted at __________________________________.

Table B-1 – Medications that can moderately or strongly inhibit or strongly induce CYP3A

Medications that inhibit CYP3A	Medications that induce CYP3A
Amprenavir<br>Aprepitant<br>Atazanavir<br>Boceprevir<br>Clarithromycin<br>Conivaptan<br>Delavirdine<br>Diltiazem<br>Erythromycin<br>Fluconazole<br>Fosamprenavir<br>Indinavir<br>Itraconazole<br>Ketoconazole<br>Lopinavir<br>Mibefradil<br>Miconazole<br>Nefazodone<br>Nelfinavir<br>Posaconazole<br>Ritonavir<br>Saquinavir<br>Telaprevir<br>Telithromycin<br>Verapamil<br>Voriconazole<br>Grapefruit, grapefruit juice, or any product containing grapefruit	Carbamazepine<br>Felbamate<br>Nevirapine<br>Phenobarbital<br>Phenytoin<br>Primidone<br>Rifabutin<br>Rifampin<br>Rifapentin<br>St. John's wort
The list provided below is not exhaustive. For a more comprehensive, frequently updated list, please visit: http://medicine.iupui.edu/clinpharm/ddis/table.aspx.	

APPENDIX C DRUG DIARY

Study Participant
Self-Administration

Patients Drug Diary
DF/HCC

For study team use only

Staff initials:	
Date dispensed:	Date returned:
# pills/caps/tabs dispensed:	# pills/caps/tabs returned:
# pills/caps/tabs that should have been taken:	
Discrepancy note:	

Participant Identifier: ______________________________
Protocol number: **16-177**
Your MD: ______________________ Phone: ______________________
Your RN: ______________________ Phone: ______________________

Study drug instructions

Study drug A: Palbociclib
Study drug B: Everolimus
Study drug C: Exemestane

Drug A (Palbociclib)
How much: Your dose is ______________________
How often: You will take each dose once per day for 21 consecutive days.
When: You should take your dose within 1 hour of a meal at the same time each day.

Drug B (Everolimus)
How much: Your dose is ______________________
How often: You will take each dose once per day.
When: You should take your dose within 1 hour of a meal at the same time each day.

Drug C (Exemestane)
How much: Your dose is ______________________
How often: You will take each dose once per day.
When: You should take your dose within 1 hour of a meal at the same time each day.

Special Instructions

You should take all three drugs at the same time each day.
If it is more than 4 hours after your regularly scheduled time, do not take that days dose.
If you inadvertently take 1 extra dose during a day, you must skip the next days dose.
If you vomit your dose, do not retake the dose.
The study drugs are to be swallowed whole, and not to be chewed, crushed or opened.
Please avoid eating grapefruit.
Rememeber to bring any unused study drug, all empty containers, and this diary with you to your next visit.
During cycle 1, you should take your dose of all three drugs in the morning. You should not take your C1D2 or C1D16 dose at home.

Dosing log

Cycle:______

For each dose days 1-21 take:
1 capsule of palbociclib
1 tablet of everolimus
1 tablet of exemestane

For each dose days 21-28 take:
1 tablet of everolimus
1 tablet of exemestane

Please, indicate the date, dose taken and any comments.

	Date	Amount taken			Time Taken	Taken within 1 hr of a meal
		Palbociclib	Everolimus	Exemestane	hh:mm am/pm	Y/N
Ex.:	1/6/2014	1	2	1	8:00 AM	Y
Day 1						
Day 2						
Day 3						
Day 4						
Day 5						
Day 6						
Day 7						
Day 8						
Day 9						
Day 10						
Day 11						
Day 12						
Day 13						
Day 14						
Day 15						
Day 16						
Day 17						
Day 18						
Day 19						
Day 20						
Day 21						
Day 22						
Day 23						
Day 24						
Day 25						
Day 26						
Day 27						
Day 28						
