# Supplementary material for: Exemestane plus everolimus and palbociclib in metastatic breast cancer: clinical response and genomic/transcriptomic determinants of resistance in a phase I/II trial
Source: Nat Commun. 2024 Mar 19;15:2446. doi: 10.1038/s41467-024-45835-6 (PMC10951222; doi:10.1038/s41467-024-45835-6)
Supplement: Supplementary file 11 — Reporting Summary [file 41467_2024_45835_MOESM11_ESM.pdf]

nature portfolio

Corresponding author(s): Nikhil Wagle, MD

Last updated by author(s): Feb 1, 2024

Reporting Summary

Nature Portfolio wishes to improve the reproducibility of the work that we publish. This form provides structure for consistency and transparency in reporting. For further information on Nature Portfolio policies, see our Editorial Policies and the Editorial Policy Checklist.

Statistics

For all statistical analyses, confirm that the following items are present in the figure legend, table legend, main text, or Methods section.

n/a	Confirmed	
☐	☒	The exact sample size (n) for each experimental group/condition, given as a discrete number and unit of measurement
☐	☒	A statement on whether measurements were taken from distinct samples or whether the same sample was measured repeatedly
☐	☒	The statistical test(s) used AND whether they are one- or two-sided<br>*Only common tests should be described solely by name; describe more complex techniques in the Methods section.*
☐	☒	A description of all covariates tested
☐	☒	A description of any assumptions or corrections, such as tests of normality and adjustment for multiple comparisons
☐	☒	A full description of the statistical parameters including central tendency (e.g. means) or other basic estimates (e.g. regression coefficient) AND variation (e.g. standard deviation) or associated estimates of uncertainty (e.g. confidence intervals)
☐	☒	For null hypothesis testing, the test statistic (e.g. F, t, r) with confidence intervals, effect sizes, degrees of freedom and P value noted<br>*Give P values as exact values whenever suitable.*
☒	☐	For Bayesian analysis, information on the choice of priors and Markov chain Monte Carlo settings
☒	☐	For hierarchical and complex designs, identification of the appropriate level for tests and full reporting of outcomes
☒	☐	Estimates of effect sizes (e.g. Cohen's d, Pearson's r), indicating how they were calculated

Our web collection on statistics for biologists contains articles on many of the points above.

Software and code

Policy information about availability of computer code

Data collection	All software and methods for genomic data generation is described in detail in the Methods or Supplemental Text. All genomics data collection software and tools used in this study are published and/or publicly available.
Data analysis	All software and pipelines for genomic analysis are described in detail in the Methods or Supplemental Text. All genomics analysis software and tools used in this study are published and publicly available.<br><br>A custom made cancer genomics analysis pipeline was used to identify somatic alterations using the Terra platform (https://app.terra.bio/). We have utilized the CGA WES Characterization pipeline developed at the Broad Institute to call, filter and annotate somatic mutations and copy number variation (available in the Terra platform public workspace broad-fc-getzlab-workflows/CGA_WES_Characterization_OpenAccess) (Documentation available in https://docs.google.com/document/d/1VO2kX_fgfUd0x3mBS9NjLUWGZu794WbTepBel3cBg08). The pipeline employs the following tools:<br><br>- MuTect version 1.1.6-0-g6fe4f4c<br>- MuTect2 - GATK v4.0.5.1<br>- ContEst - GATK v3.5-0-g36282e4<br>- Strelka version 1.0.11<br>- Orientation Bias Filter - GATK v1.5-260-g9dc161b<br>- DeTiN version v1.8.5<br>- AllelicCapSeg (Terra pipeline version)<br>- MAFPoNFilter (Terra pipeline version)

- BLAT realignment filter (Terra pipeline version)
- ABSOLUTE (Terra pipeline version)
- GATK CNV (Terra pipeline version)
- Picard Tools CrosscheckFingerprints and CollectMultipleMetrics - GATK v4.0.5.1
- Variant Effect Predictor (GATK v3.5-0-g36282e4)
- Oncotator version 1.9.9.0

To annotate known oncogenic mutations, the OncoKB annotator was used (https://github.com/oncokb/oncokb-annotator). MSigDB was used to obtain the Hallmark gene sets (version 7.2, https://www.gsea-msigdb.org/gsea/msigdb/).

Statistical significance of the association between signature activities and oncogenic mutations was measured using a one-sided Mann–Whitney test, and is based on whether the AUC ROC score outperforms a random classifier. Statistical significance of the association between the Hallmark signature scores in baseline tumors and clinical benefit was measured using a two-sided Welch's t-test. Statistical significance of the enrichment of upper or lower quartile Hallmark signature scores in the baseline tumors of patients that derived clinical benefit was measured using a two-sided Fisher exact test. A P value of < 0.05 was considered to be statistically significant. All statistical analysis was performed using R (version 4.0.3). Plots were generated using R (version 4.0.3), ggplot2 (version 3.3.5), ggpubr (version 0.4.0), and ComplexHeatmap (version 2.6.2).

For manuscripts utilizing custom algorithms or software that are central to the research but not yet described in published literature, software must be made available to editors and reviewers. We strongly encourage code deposition in a community repository (e.g. GitHub). See the Nature Portfolio guidelines for submitting code & software for further information.

Data

Policy information about availability of data

All manuscripts must include a data availability statement. This statement should provide the following information, where applicable:

- Accession codes, unique identifiers, or web links for publicly available datasets
- A description of any restrictions on data availability
- For clinical datasets or third party data, please ensure that the statement adheres to our policy

Tumor and germline whole-exome sequencing data and RNA sequencing data generated and analyzed for this study have been deposited in the database of Genotypes and Phenotypes (dbGaP) under study accession phs001285.v2.p1. These data are available under controlled access to protect individual's privacy. Access can be requested through dbGAP and use restrictions are specified by the Health/Medical/Biomedical and Disease-Specific (Breast Cancer) consent groups. Processed de-identified data generated in this study including clinical trial protocol and data, patient metadata, and tumor exome and transcriptome analysis are available in Supplementary Data 1-8. posted online. Data for each figure panel is available in Source Data. Four patients (patients 7, 26, 36, and 37) did not co-consent to the additional DF/HCC Protocols 05-246/09-204, and their genomic data is not included in Supplementary Data 2 and was not be deposited in dbGaP. Reasonable requests for additional raw and processed data and materials will be reviewed by the senior authors to determine whether the request is subject to any intellectual property or confidentiality obligations. These additional data and materials may be subject to patient confidentiality and might require a material transfer agreement.

Human research participants

Policy information about studies involving human research participants and Sex and Gender in Research.

Reporting on sex and gender	Male and female participants were eligible to participate in the study. The sex of all participants in the study was female (N=9 in the phase Ib portion, N=32 in the phase IIa portion), as expected from the higher prevalence of breast cancer in women when compared to men. Thus, all analyses included only female participants.
Population characteristics	Eligible patients had been diagnosed with HR+/HER2- metastatic breast cancer and had progressed on a prior CDK4/6i and a prior endocrine therapy (a nonsteroidal aromatase inhibitor). Any number of prior endocrine therapies were allowed, as long as none were exemestane-based. Up to one prior line of chemotherapy was allowed. The sex of all patients was female. In the phase II portion of the trial the median age (range) was 55.5 years (36-73), 37.5% (12/32) had received one line of prior chemotherapy, 96.9% (31/32) had received one prior line of endocrine therapy, and 53.1% (17/32) had received two prior lines of endocrine therapy.
Recruitment	Physicians within the Dana-Farber Cancer Institute Breast Oncology group use a web-based application (http://dfcibreastclinicaltrials.org/) that has all active clinical trials within it to help them review trial options for their patients. This allows physicians to be aware of all available trials to help prevent bias. Patients were consented and treated by study investigators at Dana-Farber Cancer Institute. Compared to other clinical trials evaluating the use of a CDK4/6i after progression of a CDK4/6i (e.g. TRINITI-1), the population was more heavily pretreated in terms of prior lines of endocrine therapy and chemotherapy, which could have resulted in a lower CBR for this trial.
Ethics oversight	The study was conducted in accordance with the International Conference on Harmonization Good Clinical Practice Standards and the Declaration of Helsinki. Institutional review board (IRB) approval was obtained at Dana-Farber/Harvard Cancer Center (DF/HCC). The DF/HCC Data and Safety Monitoring Committee (DSMC), which is composed of clinical specialists with experience in oncology and who had no direct relationship with the study, reviewed and monitored toxicity and accrual data from the study. Information that raised questions or concerns was addressed with the overall PI and study team. Participants provided written informed consent prior to the performance of any protocol specific procedures or assessments.

Note that full information on the approval of the study protocol must also be provided in the manuscript.

Field-specific reporting

Please select the one below that is the best fit for your research. If you are not sure, read the appropriate sections before making your selection.

[x] Life sciences [ ] Behavioural & social sciences [ ] Ecological, evolutionary & environmental sciences

For a reference copy of the document with all sections, see nature.com/documents/nr-reporting-summary-flat.pdf

Life sciences study design

All studies must disclose on these points even when the disclosure is negative.

Sample size	For the phase Ib portion of the clinical trial, the number of participants (N=9) was not pre-planned and was chosen to be the minimum number of patients sufficient to obtain the MTD/RP2D, the primary objective of phase Ib, using a 3+3 dose escalation design. At the starting dose of palbociclib (100 mg), 1 out of 3 patients experienced a dose-limiting toxicity. Subsequently, 3 additional patients were initiated at 100 mg, and none experienced a dose-limiting toxicity. Palbociclib was increased to 125 mg, and all 3 patients had a dose-limiting toxicity. Thus, following the 3+3 dose escalation algorithm, 100 mg palbociclib was declared the MTD and RP2D, and N=9 patients was the total number of participants.<br><br>For the phase IIa portion of the clinical trial, the number of participants (N=32) was pre-planned and determined from the minimum of 29 participants needed to test the hypothesis of a CBR>=65% with 90% power and a one-sided alpha of 0.1, and a drop out rate of 10%. No formal sample size calculation was performed for the genomic analyses. All available samples from the phase II portion of the trial that passed quality control filters were included in the genomic analyses.
Data exclusions	All available samples from the phase II portion of the trial that passed quality control filters were included in the genomic analyses. Samples from the phase I portion of the trial were not analyzed or included in the genomic analyses because only 1 patient had sequenced data generated and because of the distinct doses used in the phase I compared to the phase II portion of the trial. 2 tumor samples with a Normal PAM50 subtype were excluded from the transcriptomic analyses, since it is indicative of the low tumor content. The data from these 2 tumor Normal PAM50 tumor samples was included in Supplemental Data 2 and, for completeness, in Supplemental Fig. S4.<br><br>As detailed in the Supplemental Text, in almost all the transcriptomic analyses, the log2(TPM+1) expression values that correct for the differences between frozen and FFPE tissue were used. The exception was the Hallmark signature analysis in Fig. 7B and Tab 1 in Supplemental Table S11, where we only considered frozen tissue (n = 15 tumor samples, which excludes 2 samples with a Normal PAM50, and 1 FFPE tissue sample) and used the uncorrected log2(TPM+1) expression values to avoid any potential biases introduced in the tissue-type corrected expression values.
Replication	No replication was performed in this study because of the limited tumor tissue available from patient biopsies.
Randomization	This study was an open-label, single-arm phase Ia/IIb clinical trial. There was no randomization because the study had a single treatment arm.
Blinding	This study was an open-label, single-arm phase Ia/IIb clinical trial. Investigators were not blinded because the study had a single treatment arm and was open-label.

Reporting for specific materials, systems and methods

We require information from authors about some types of materials, experimental systems and methods used in many studies. Here, indicate whether each material, system or method listed is relevant to your study. If you are not sure if a list item applies to your research, read the appropriate section before selecting a response.

Materials & experimental systems

n/a	Involved in the study	
[x]	[ ]	Antibodies
[x]	[ ]	Eukaryotic cell lines
[x]	[ ]	Palaeontology and archaeology
[x]	[ ]	Animals and other organisms
[ ]	[x]	Clinical data
[x]	[ ]	Dual use research of concern

Methods

n/a	Involved in the study	
[x]	[ ]	ChIP-seq
[x]	[ ]	Flow cytometry
[x]	[ ]	MRI-based neuroimaging

Clinical data

Policy information about clinical studies

All manuscripts should comply with the ICMJEguidelines for publication of clinical research and a completedCONSORT checklist must be included with all submissions.

Clinical trial registration	NCT02871791
Study protocol	Initial and final study protocols are available in Supplemental Data 1
Data collection	A total of 41 eligible participants were recruited from September 12, 2016 to June 26, 2019. A total of 9 patients were recruited into the phase Ib portion of the study, and a total of 32 were recruited into the phase IIa portion of the study. Relevant clinical data was collected during each patients visit to Dana-Farber Cancer Institute in Boston, Massachusetts. Tumor samples were collected retrospectively (archival primary or metastatic tumors) and prospectively (baseline tumor and blood samples prior to initiating protocol therapy, and blood samples during protocol therapy).
Outcomes	The primary objective of phase Ib was to determine the safety and tolerability of the trial's therapy, and to define the maximum tolerated dose (MTD) and recommended phase II dose (RP2D). The secondary objectives of this phase were to describe the pharmacokinetics (PK) profile of everolimus and exemestane in the protocol therapy and evaluate the potential effect of palbociclib on the PK profile of everolimus. To determine MTD/RP2D, participants were treated with increasing/decreasing doses of palbociclib and everolimus to establish the MTD/RP2D for both drugs in the context of the trial's therapy. Participants proceeded in dose escalation following the 3 + 3 rule. Treatment-related toxicities were summarized by maximum grade and by term using CTCAE version 4.0. The starting dose of palbociclib was 100 mg, which was increased to 125 mg. The starting dose of everolimus was 5 mg, which was increased to 10 mg. Only one of the two study drugs was escalated/de-escalated at a time. If patients developed toxicity to 5 mg everolimus, de-escalation to 2.5 mg was allowed. Palbociclib doses below 100 mg were not explored. Exemestane was maintained at 25 mg.<br><br>The primary endpoint of phase IIa was to determine the Clinical Benefit Rate (CBR) of the trial's therapy. The primary endpoints of phase IIa were the Overall Response Rate (ORR), the Disease Control Rate (DCR), the Duration of Response (DOR), and the Median Progression Free Survival (PFS). Patients were evaluated for response every 8 weeks according to RECIST 1.1 criteria. Response was assessed among participants eligible for the phase IIa part of the study who received at least one dose of the study drugs at the MTD/RP2D and have measurable disease at screening. CBR is defined as the proportion of participants achieving complete response, partial response or stable disease for more than 6 months (CR+PR+SD ≥ 24 weeks) taking as reference the smallest measurements recorded since the treatment started, including the baseline measurements. ORR was defined as the proportion of participants achieving CR or PR. DCR was defined as the proportion of patient that has CR+PR+SD>=12 weeks. DOR was defined based on the duration of stable disease. PFS based on the Kaplan-Meier method is defined as the duration of time from study entry to documented disease progression (PD) or death.
